# Supplementary material for: Synthesis and Biological Evaluation of Lipophilic Nucleoside Analogues as Inhibitors of Aminoacyl-tRNA Synthetases
Source: Antibiotics (Basel). 2019 Oct 9;8(4):180. doi: 10.3390/antibiotics8040180 (PMC6963541; doi:10.3390/antibiotics8040180)
Supplement: Supplementary file 1 [file antibiotics-08-00180-s001.pdf]

Supplementary Information

Synthesis and Biological Evaluation of Lipophilic Nucleoside Analogues as Inhibitors of Aminoacyl-tRNA Synthetases

By Manesh Nautiyal[1], Bharat Gadakh[1], Steff De Graef[2], Luping Pang[1, 2], Masroor Khan[1], Yi Xun[1], Jef Rozenski[1], Arthur Van Aerschot[1],*.

[1] Medicinal Chemistry, Rega Institute for Medical Research, Herestraat 49 box 1041, B-3000 Leuven, Belgium
[2] Laboratory for Biocrystallography, Department of Pharmaceutical and Pharmacological Sciences, Herestraat 49 box 822, B-3000 Leuven, Belgium

* To whom correspondence should be addressed. Tel: +32 16 37 26 24; Fax: +32 16 3 37340; Email: arthur.vanaerschot@kuleuven.be

Contents

Supplementary reaction scheme S1..1

C6 purine derivatives - intermediate & final compounds - NMR & MS 3

Prodrugs intermediate & final compounds - NMR & MS.................................32

Supplementary reaction scheme S1.

Following **reaction scheme 1**, the plan was to make a general scaffold (**5**) where various substitutions could be introduced using **reaction (iv)**. This reaction scheme proved to be useful for the introduction of N-substituted aliphatic sidechains, albeit with low yields due to degradation of the scaffold (due to the cumulative negative effect of base and high temperature). Unfortunately,

nucleophilic substitution did not work out for the less nucleophilic anilines. Different base catalysts substituting for potassium carbonate like DIPEA and DMAP were also tried combined with heating but to no avail.

Initially proposed plan for the synthesis of alkylated compounds: -

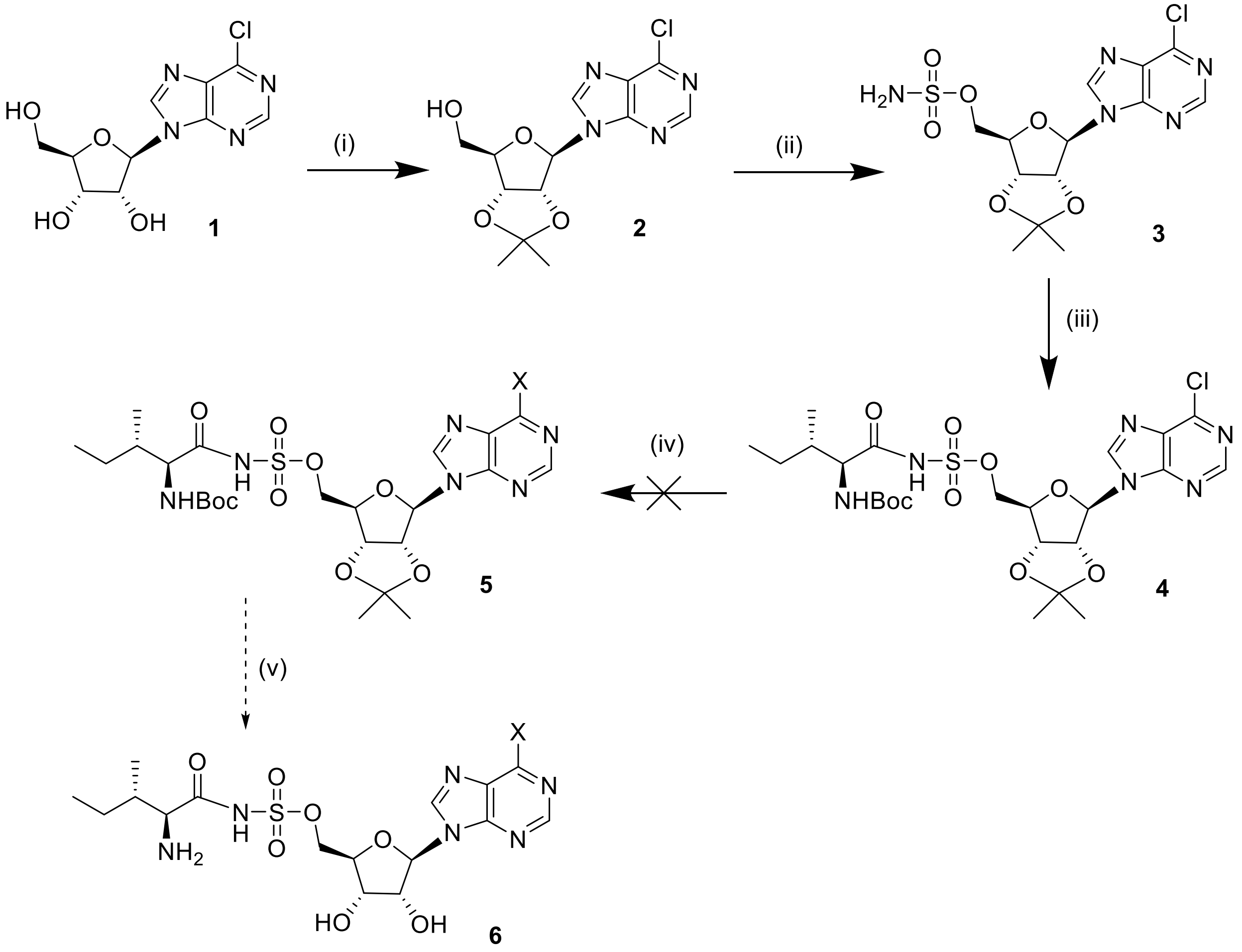

Supplementary reaction scheme S1. **Reagents and conditions:** (i) Acetone, DMP, PTSA, RT, overnight, rt; (ii) (a) CSI, HCOOH, 0 °C, 15 min; (b) ACN, 4-6h, rt; (c) DMA, overnight, rt; (iii) DBU, Boc-Ile-Osu, DMF, overnight, rt; (iv) K_2CO_3, R-NH_2, DMF, 80 °C, overnight; (v) TFA/H_2O (1:1 v/v), 3 hours, RT.

C6 purine derivatives - intermediate & final compounds - NMR & MS

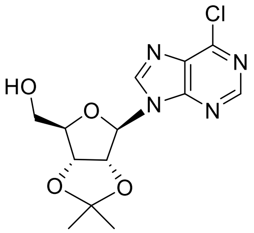

Exact Mass: 326.0782

2, 3'-isopropylidene 6-chloropurine riboside (2)

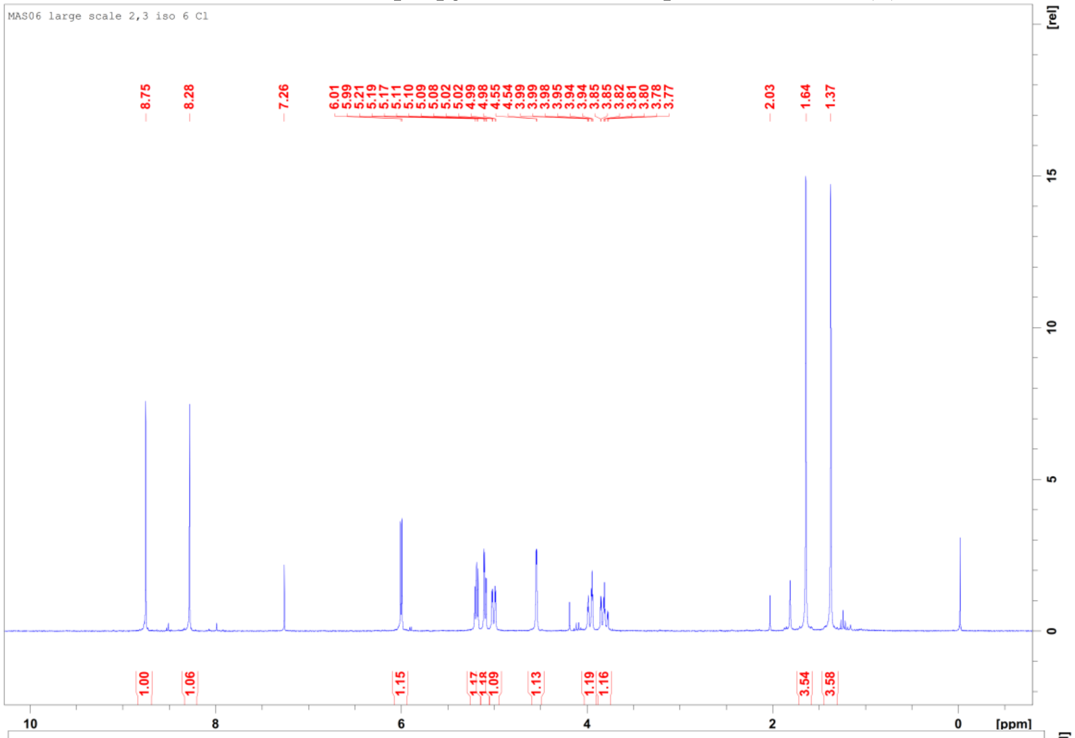

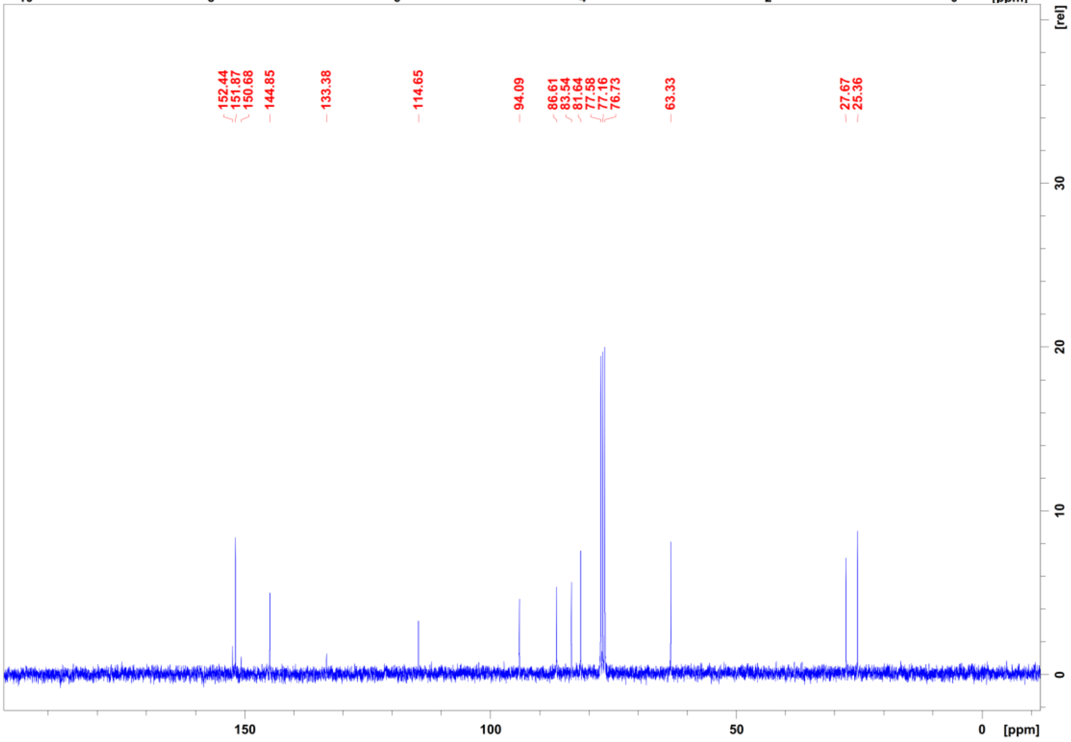

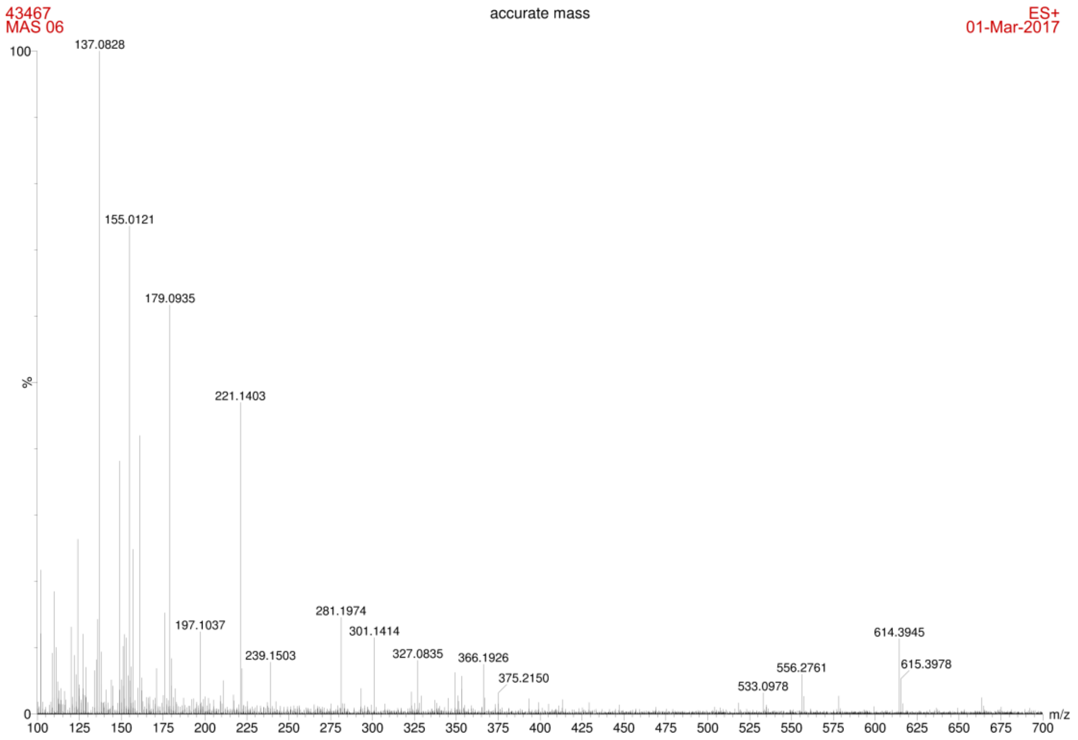

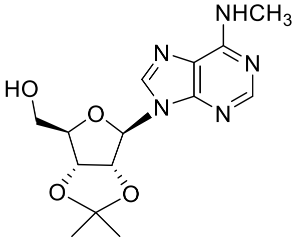

Exact Mass: 321.1437

2’,3’-isopropylidene-N6-(methyl)-adenosine (3a)

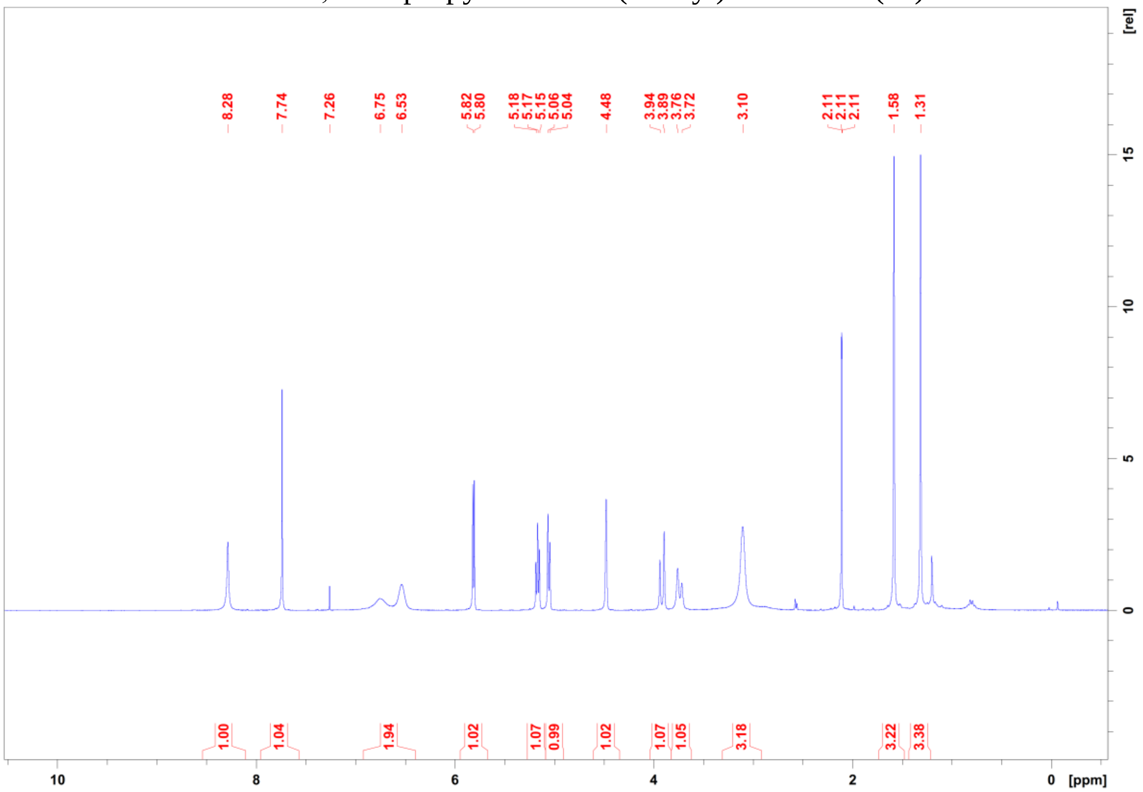

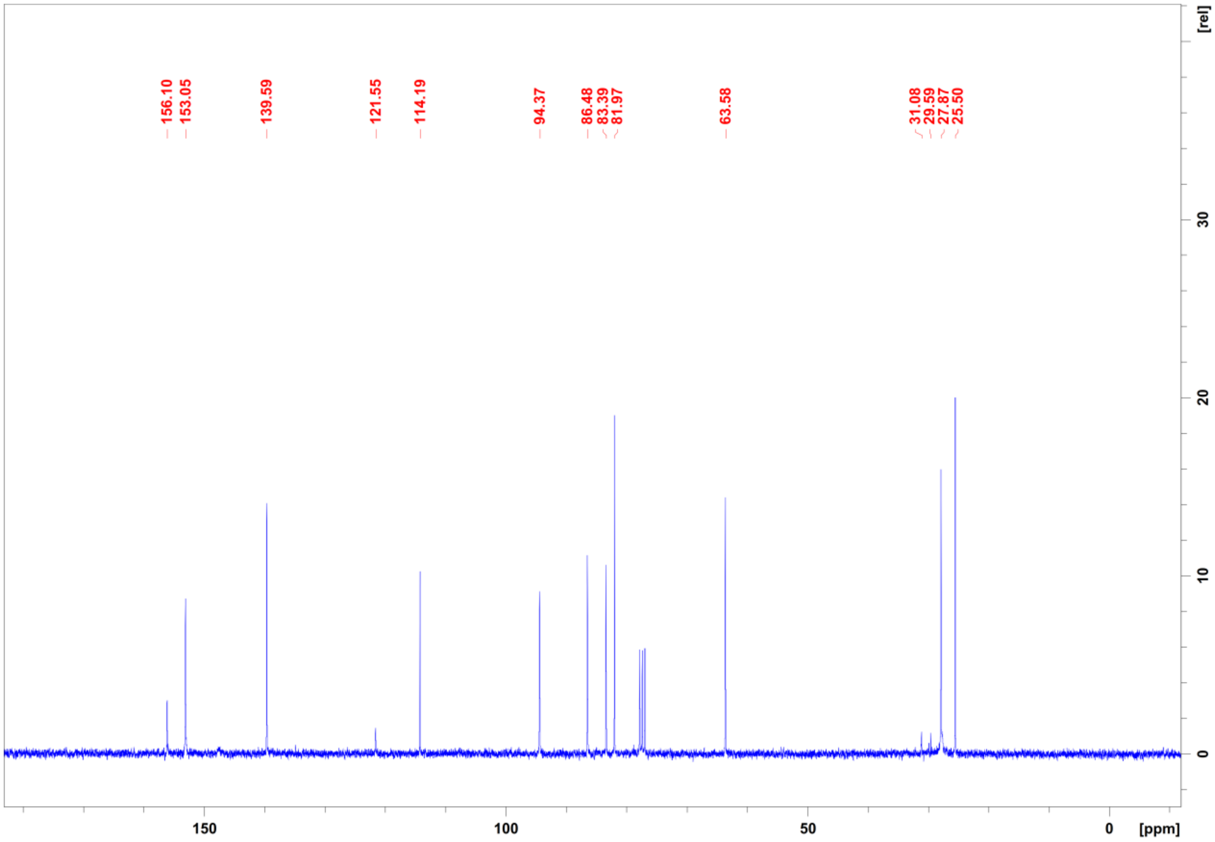
156.10
153.05
139.59
121.55
114.19
94.37
86.48
83.39
81.97
63.58
31.08
29.59
27.87
25.50
[rel]
30
20
10
0
150
100
50
0
[ppm]

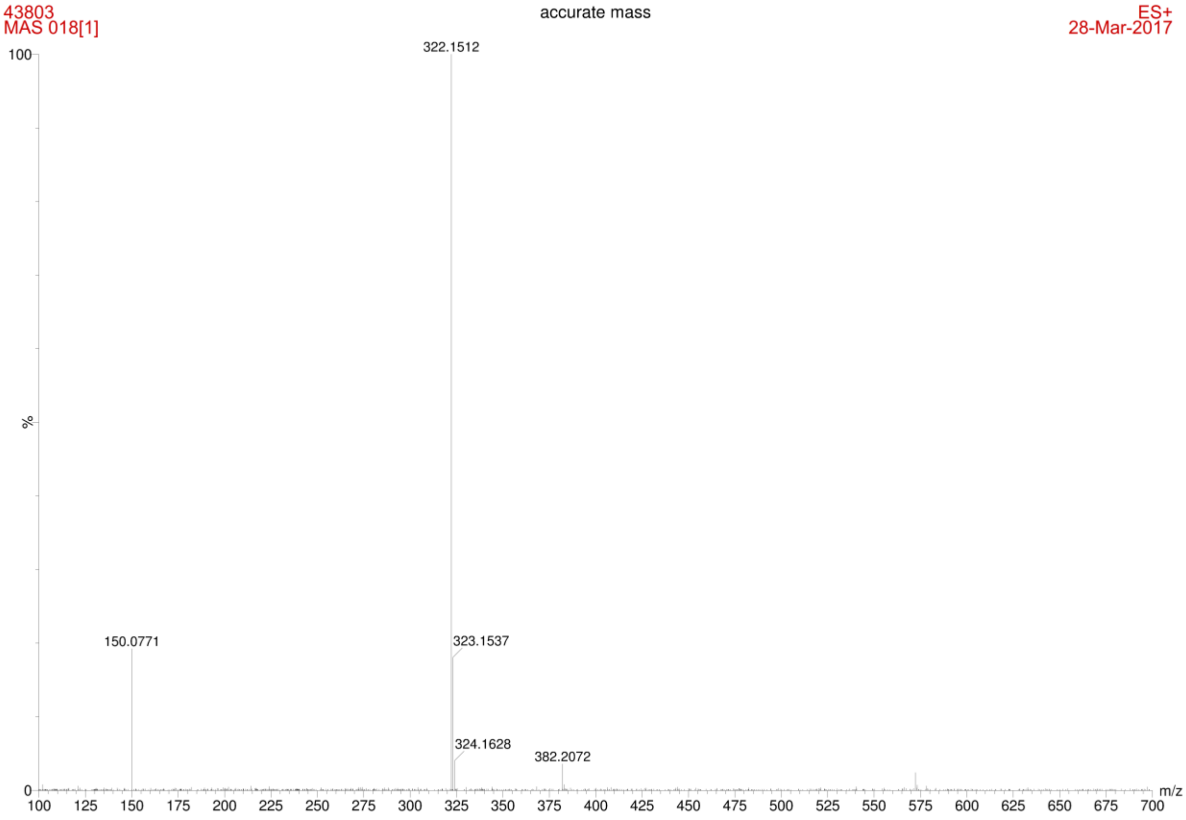
43803
MAS 018[1]
accurate mass
ES+
28-Mar-2017
100
%
0
322.1512
150.0771
323.1537
324.1628
382.2072
m/z
100 125 150 175 200 225 250 275 300 325 350 375 400 425 450 475 500 525 550 575 600 625 650 675 700

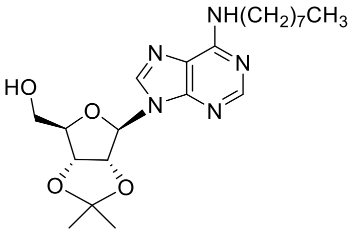

Exact Mass: 419.2533

2’,3’-isopropylidene-N6-(octyl)-adenosine (3b)

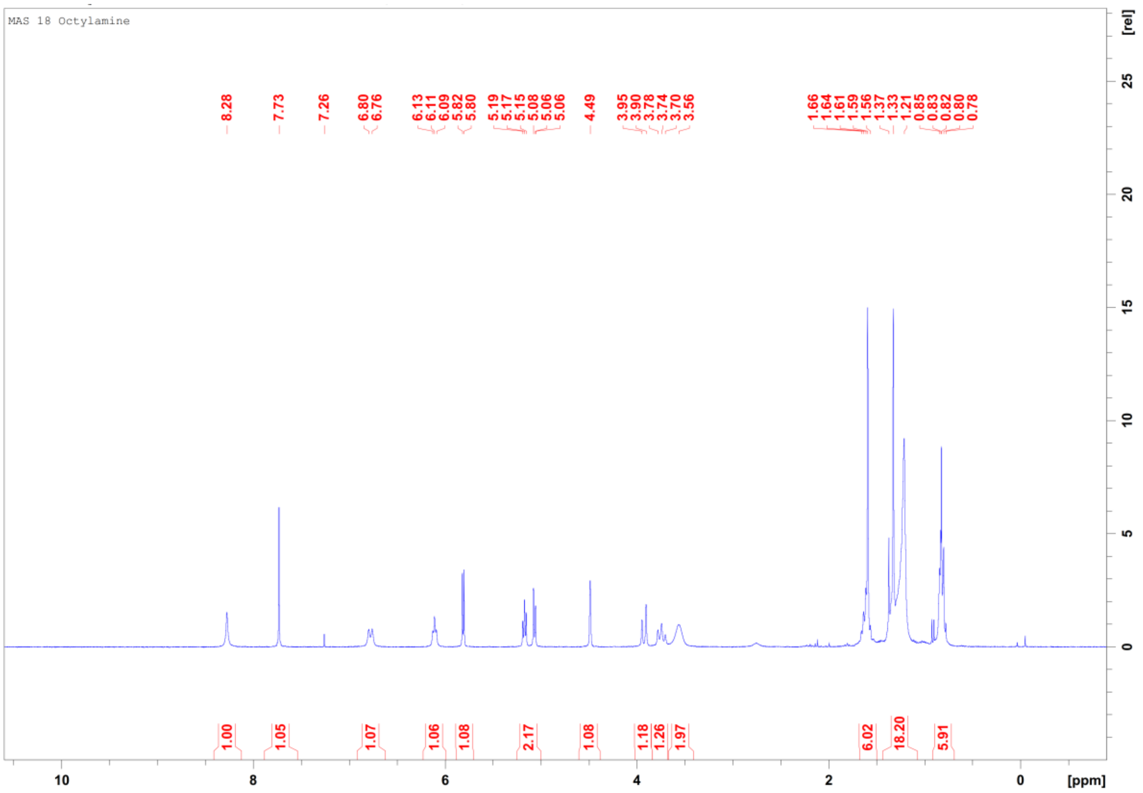

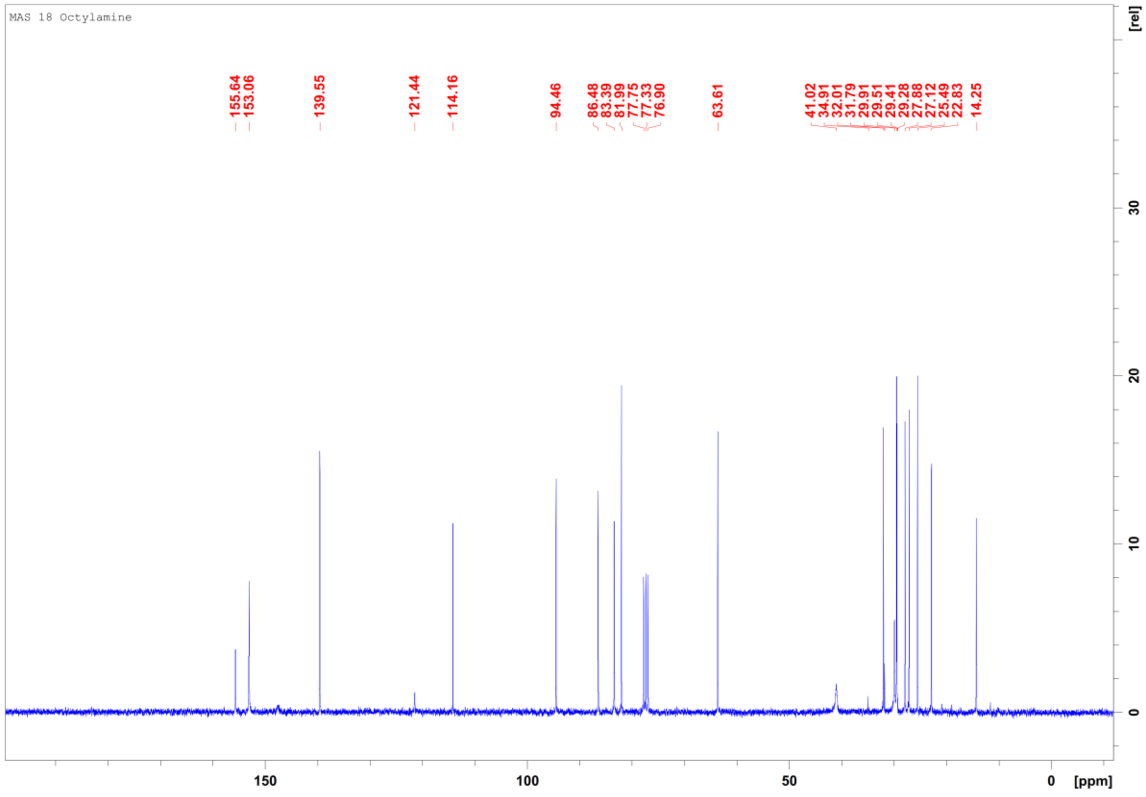

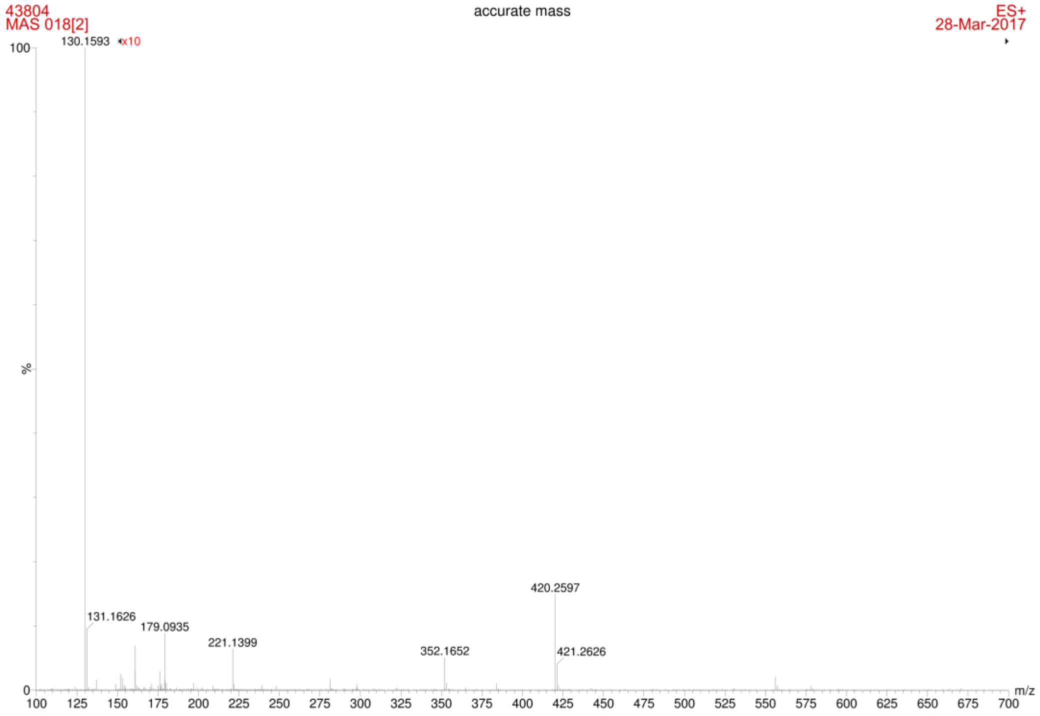

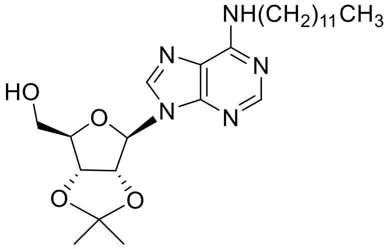

Exact Mass: 475.3159

2’,3’-isopropylidene-N6-(dodeceyl)-adenosine (3c)

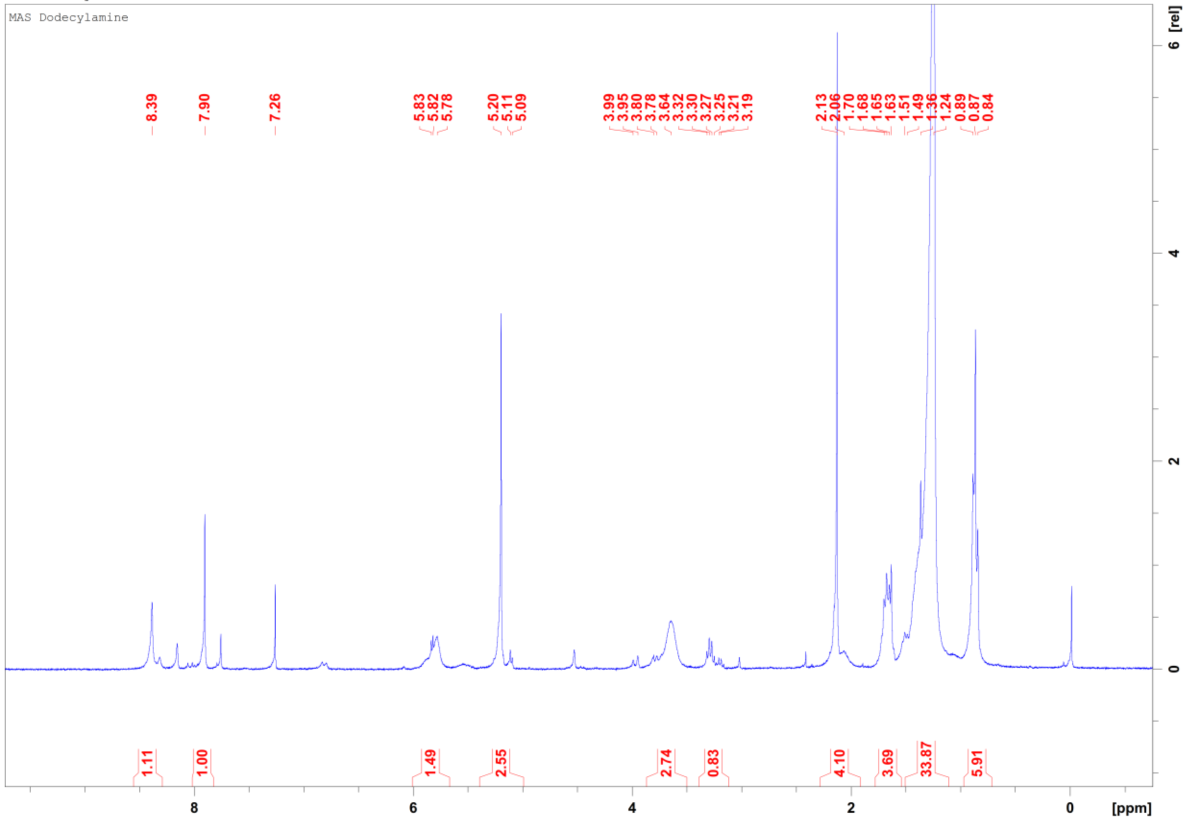

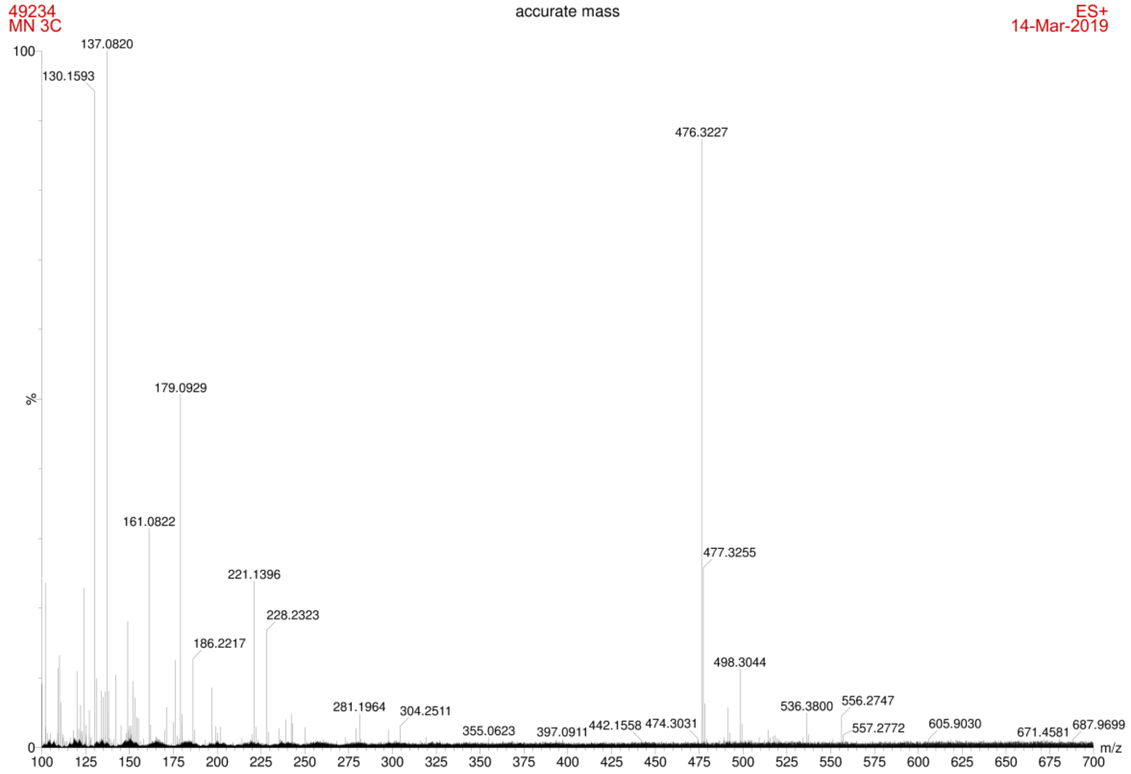

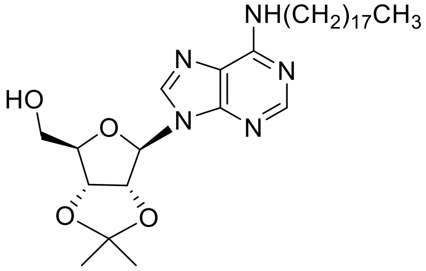

Exact Mass: 559.4098

2’,3’-isopropylidene-N6-(octadecyl)-adenosine (3d)

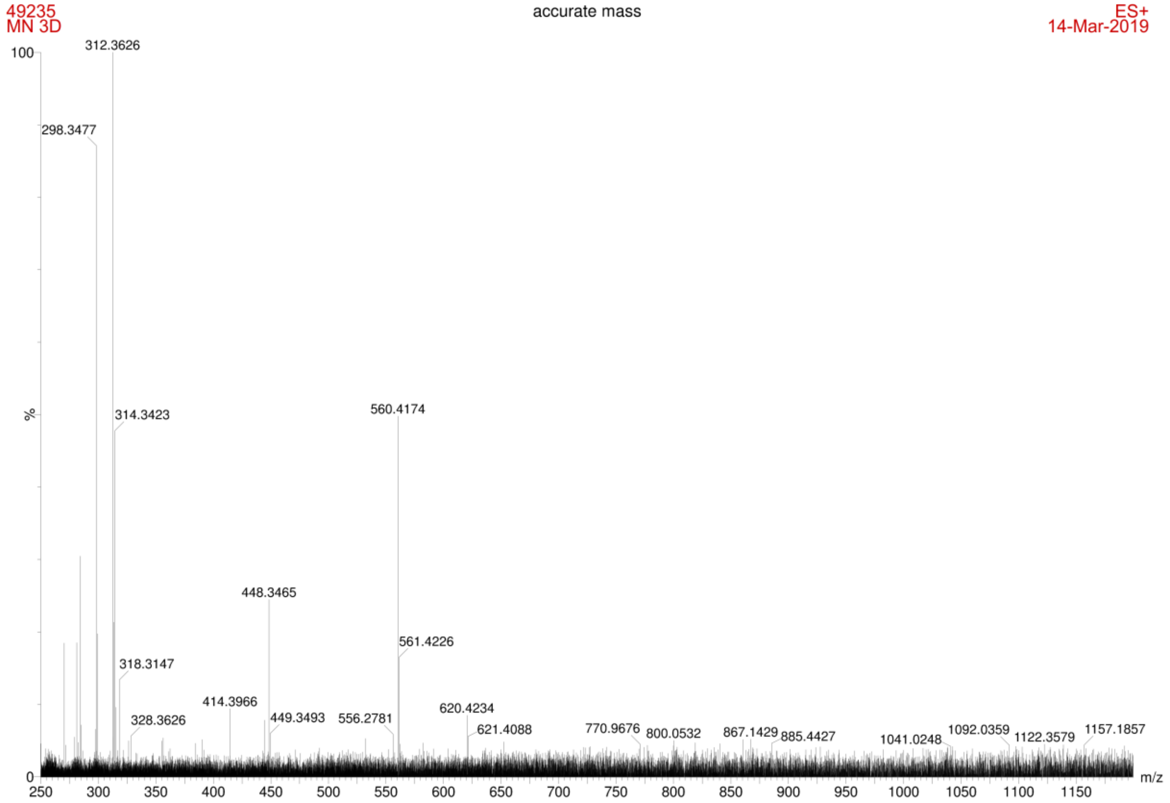

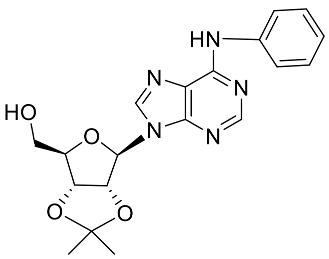

Exact Mass: 383.1594

2’,3’-isopropylidene-N6-(phenyl)-adenosine (3e)

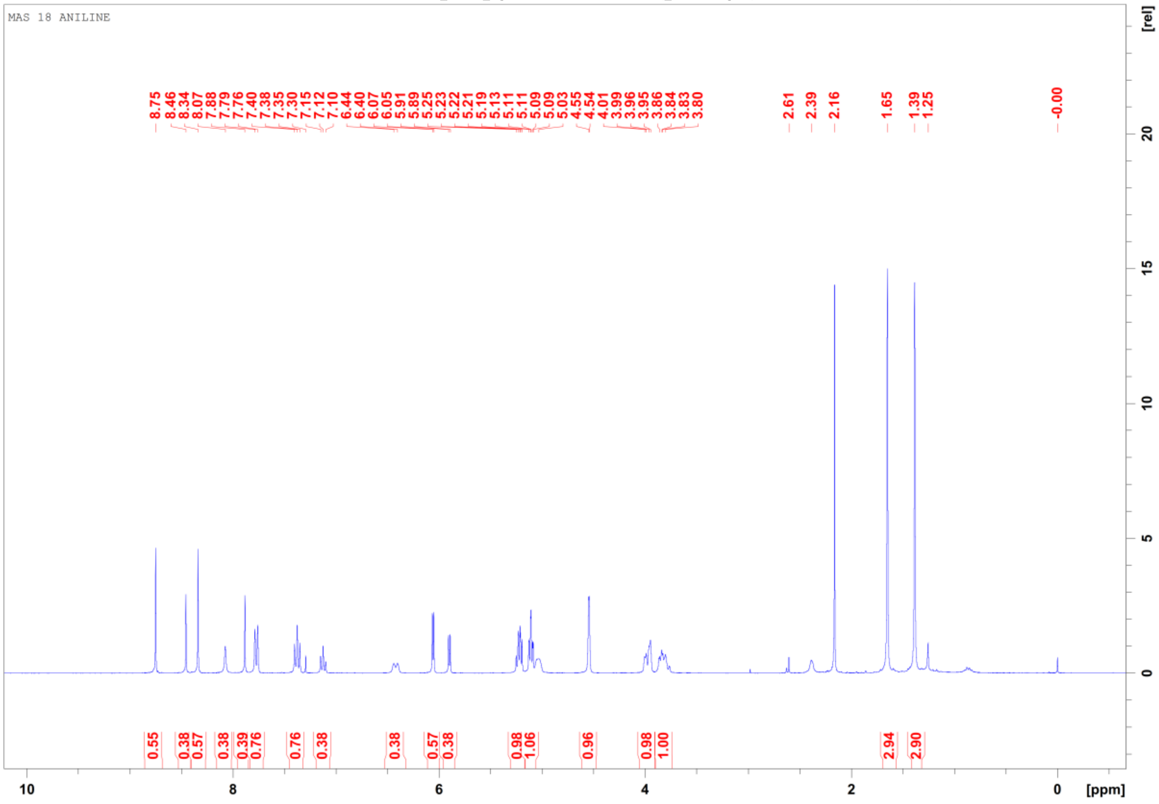

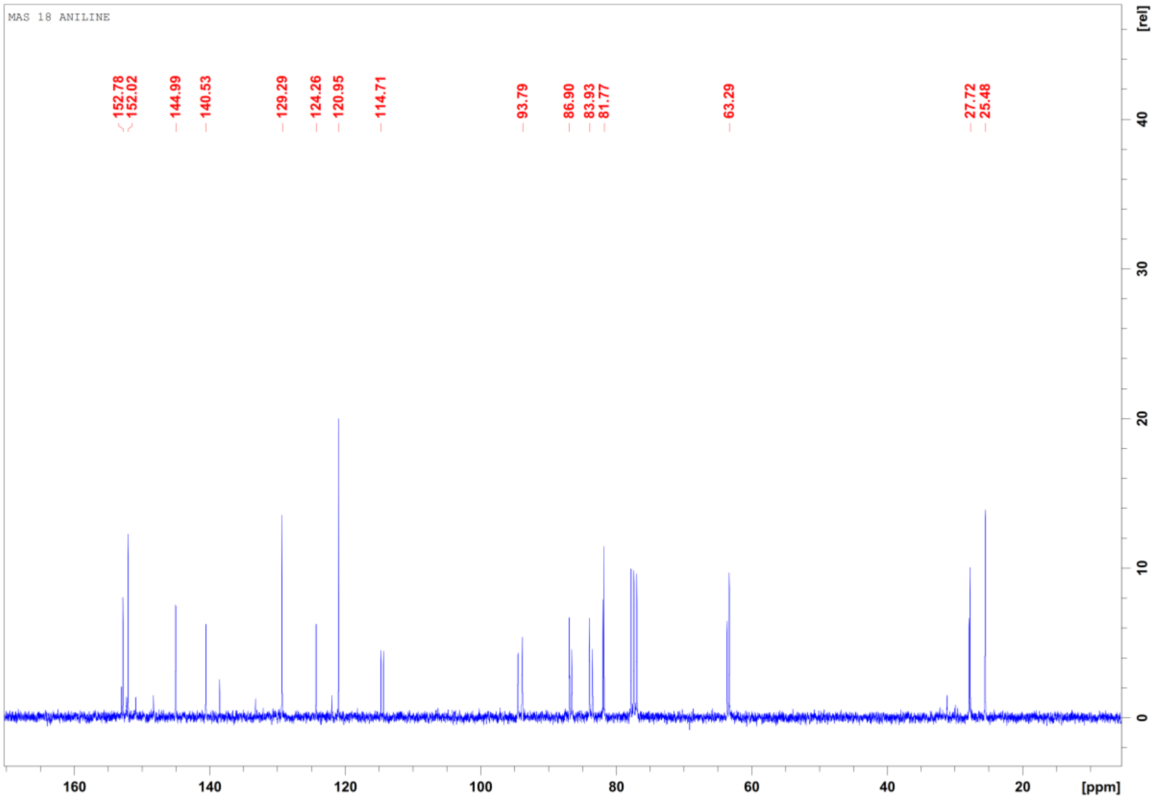

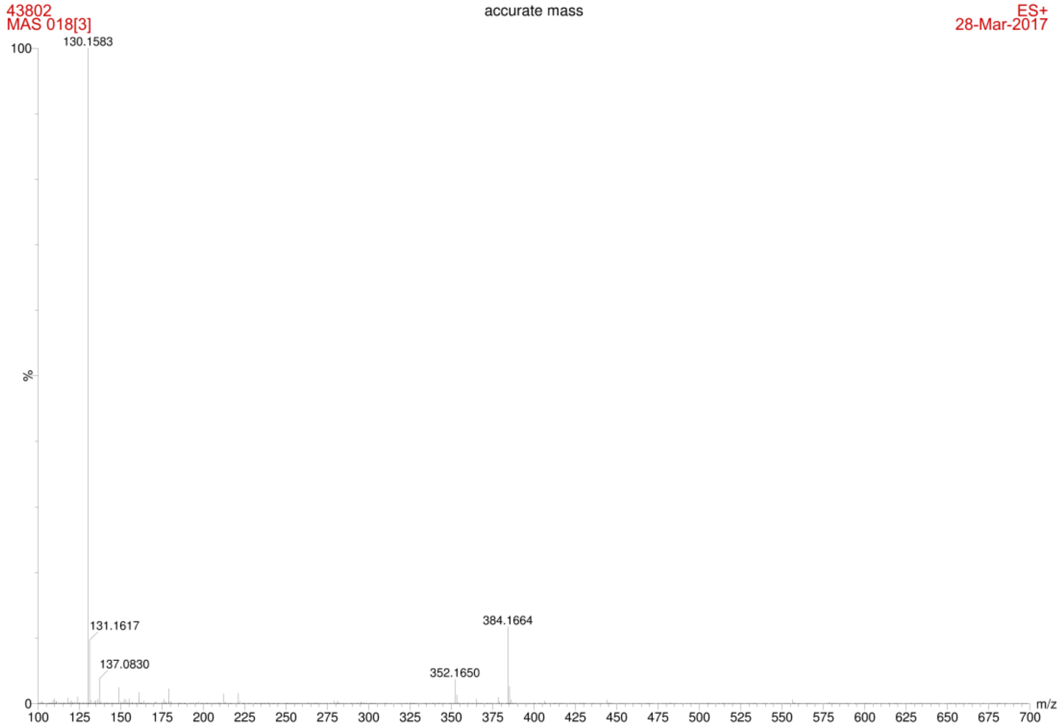

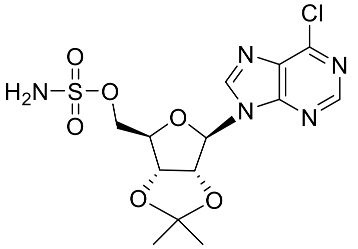

Exact Mass: 405.0510

2’, 3’-isopropylidene-5’-O-sulfamoyl-6-chloropurine riboside (3f)

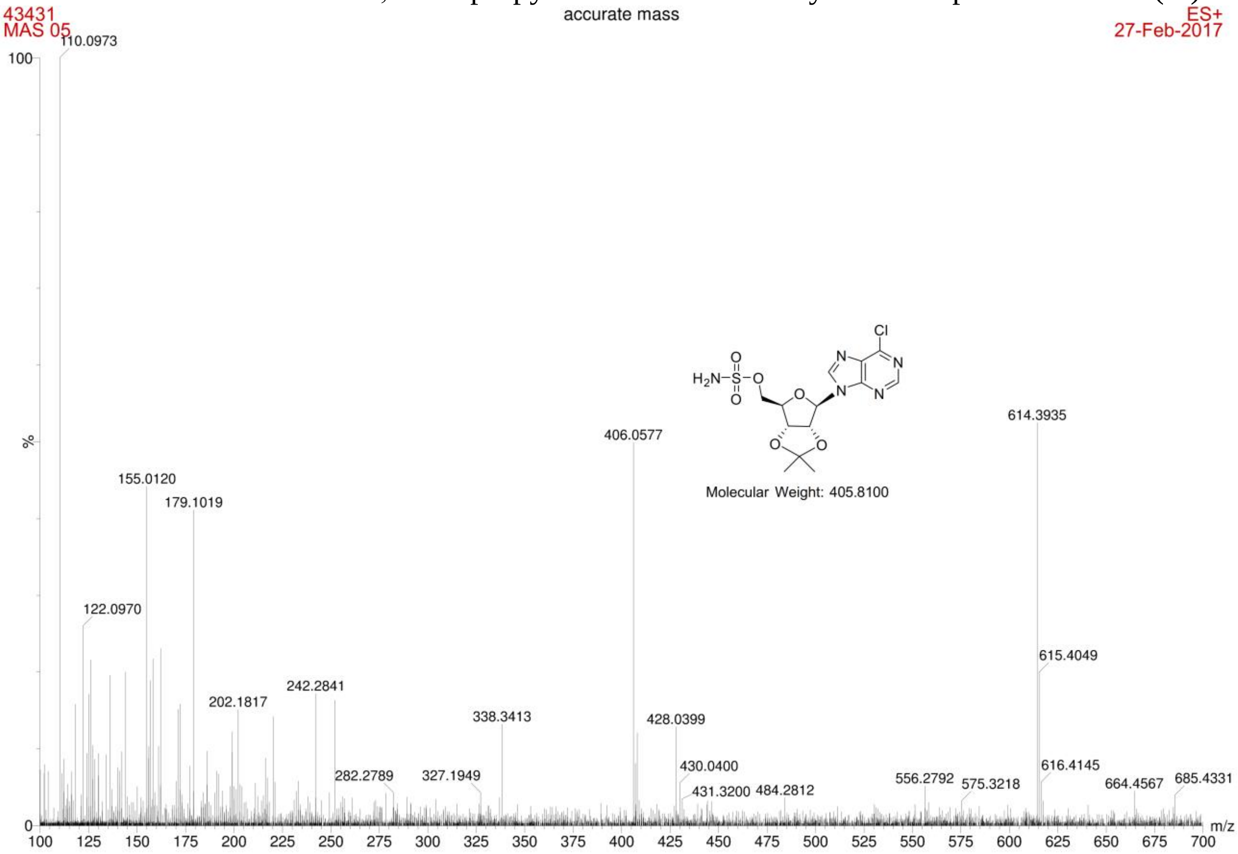

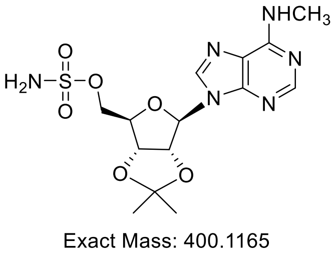

2’,3’-isopropylidene-5’-O-sulfamoyl-N6-methyl-adenosine (4a)

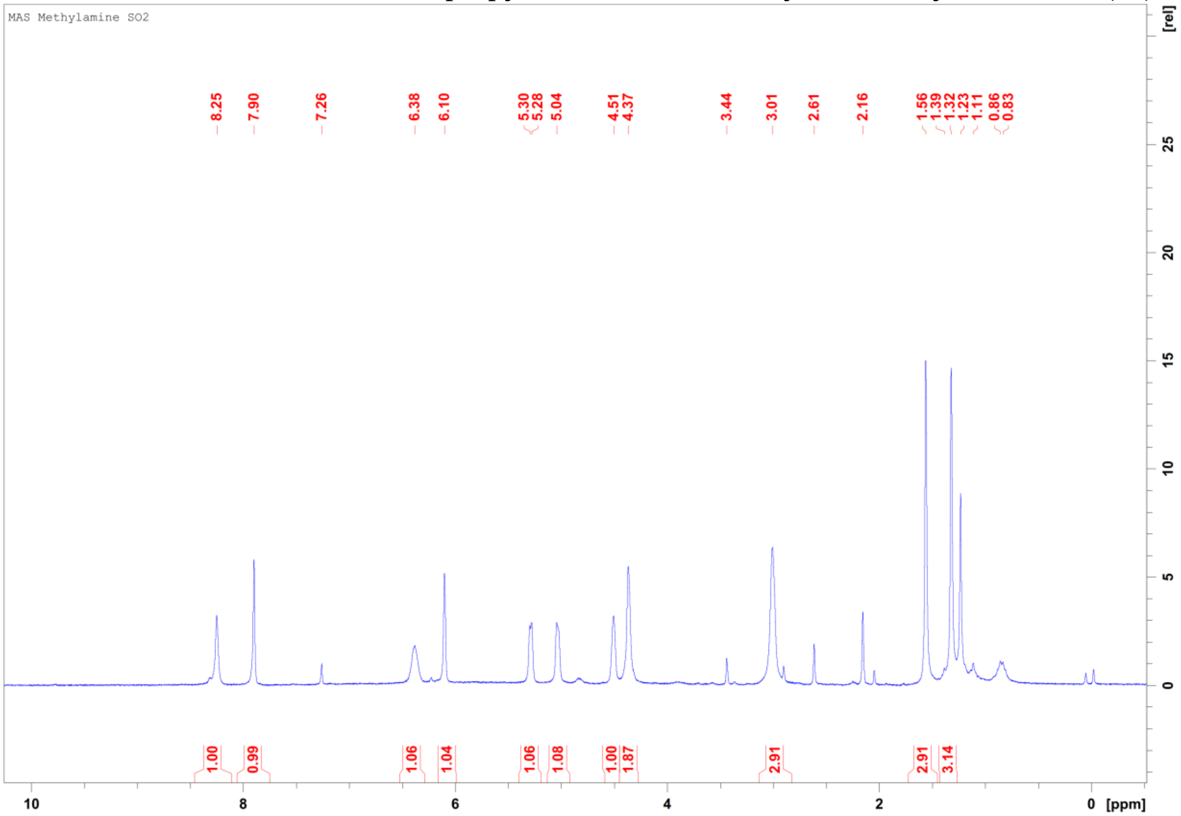

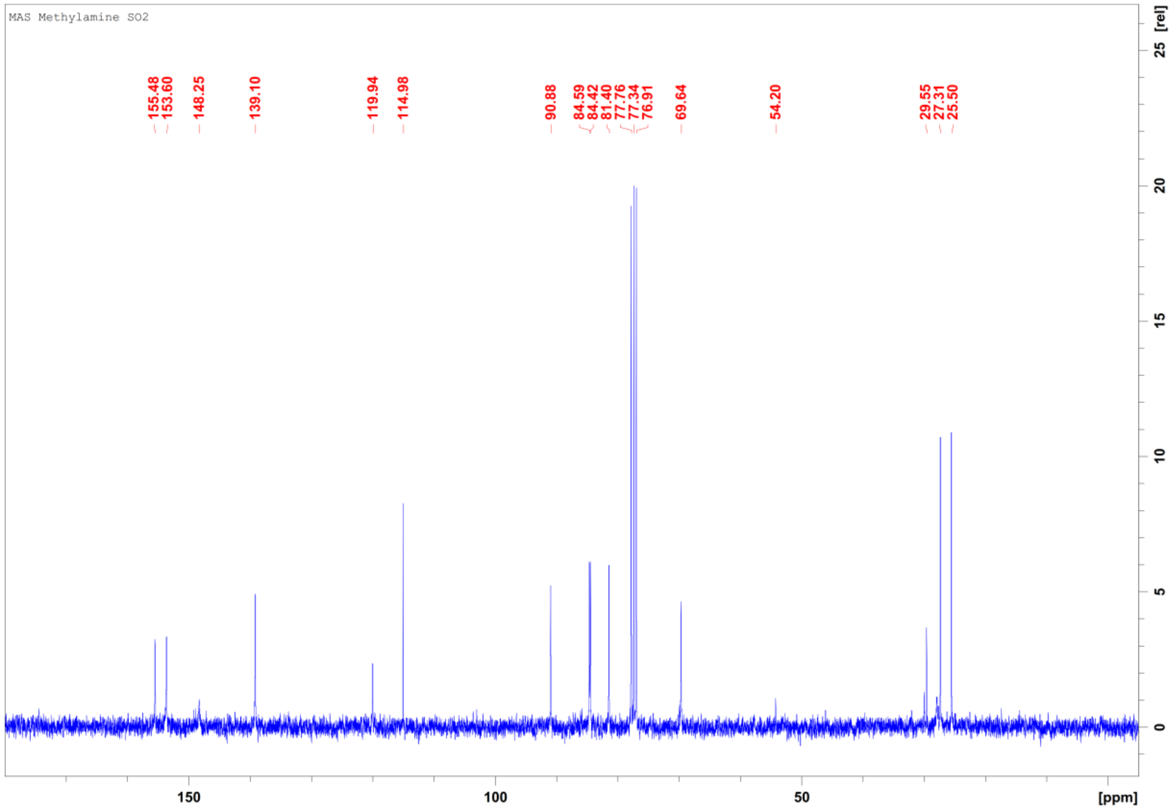

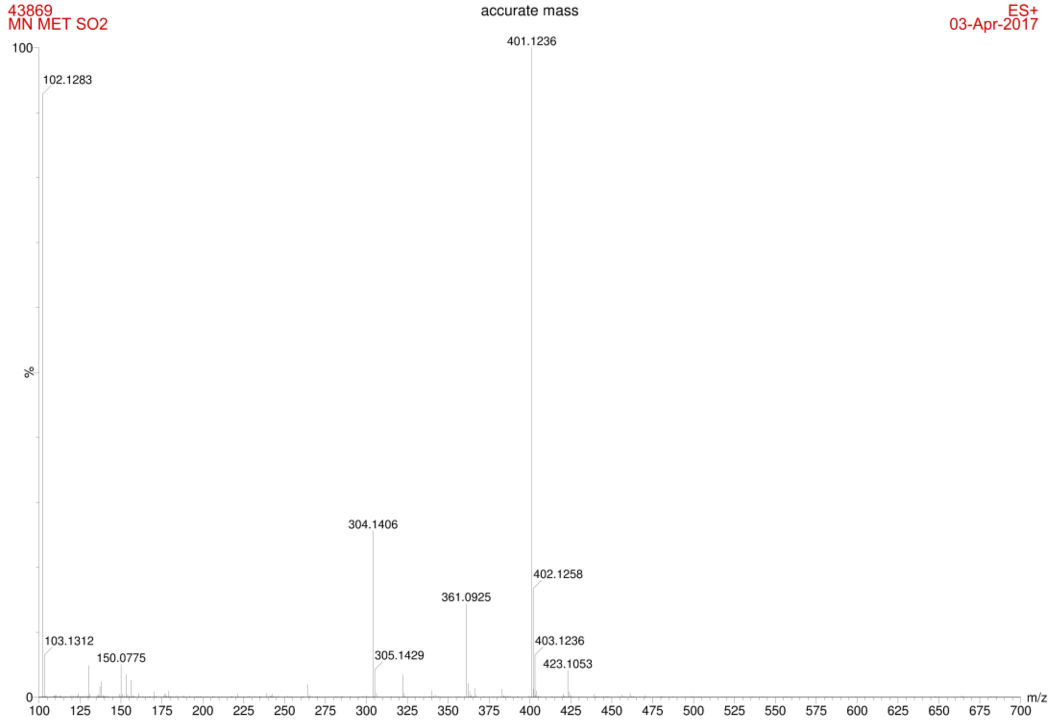

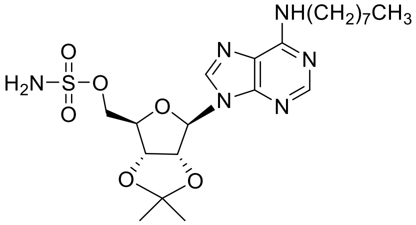

Exact Mass: 498.2261

2’,3’-isopropylidene-5’-O-sulfamoyl-N6-octyl-adenosine (4b)

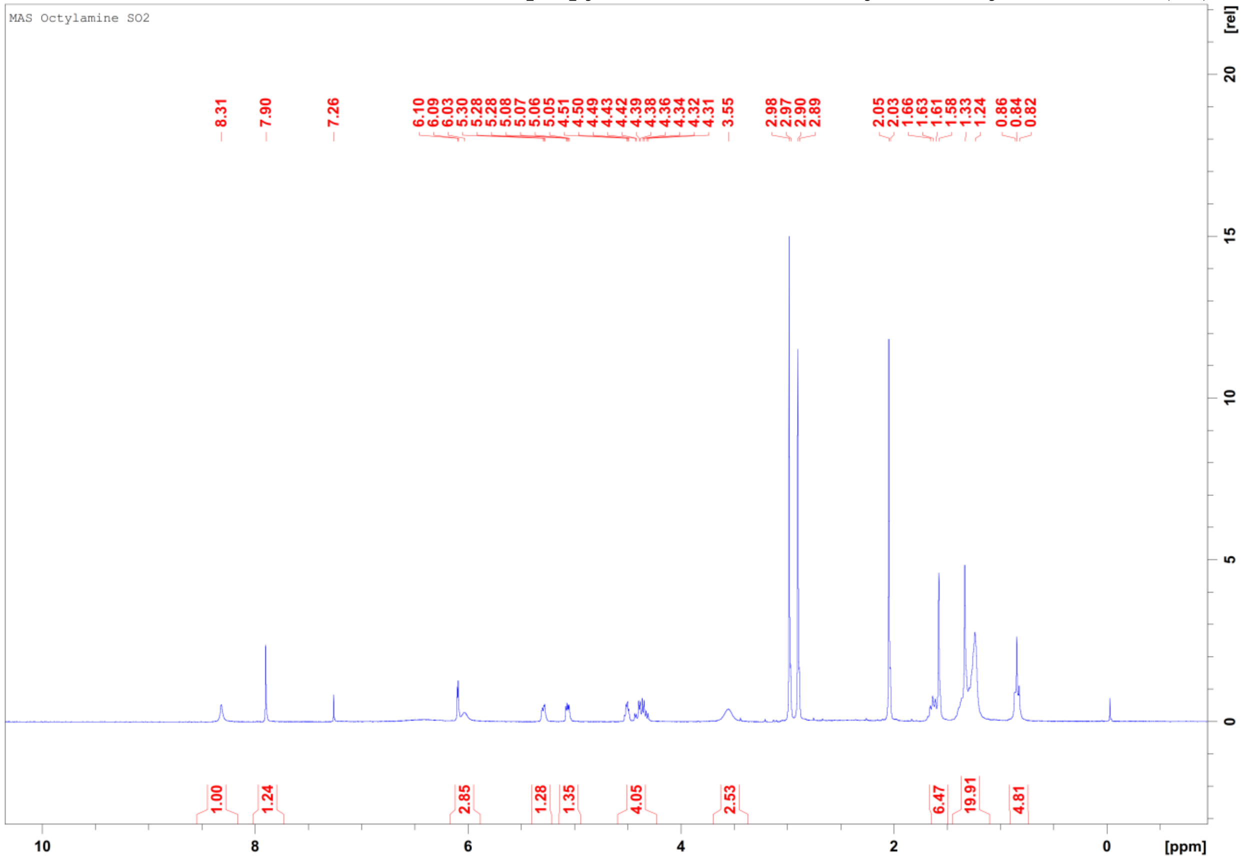

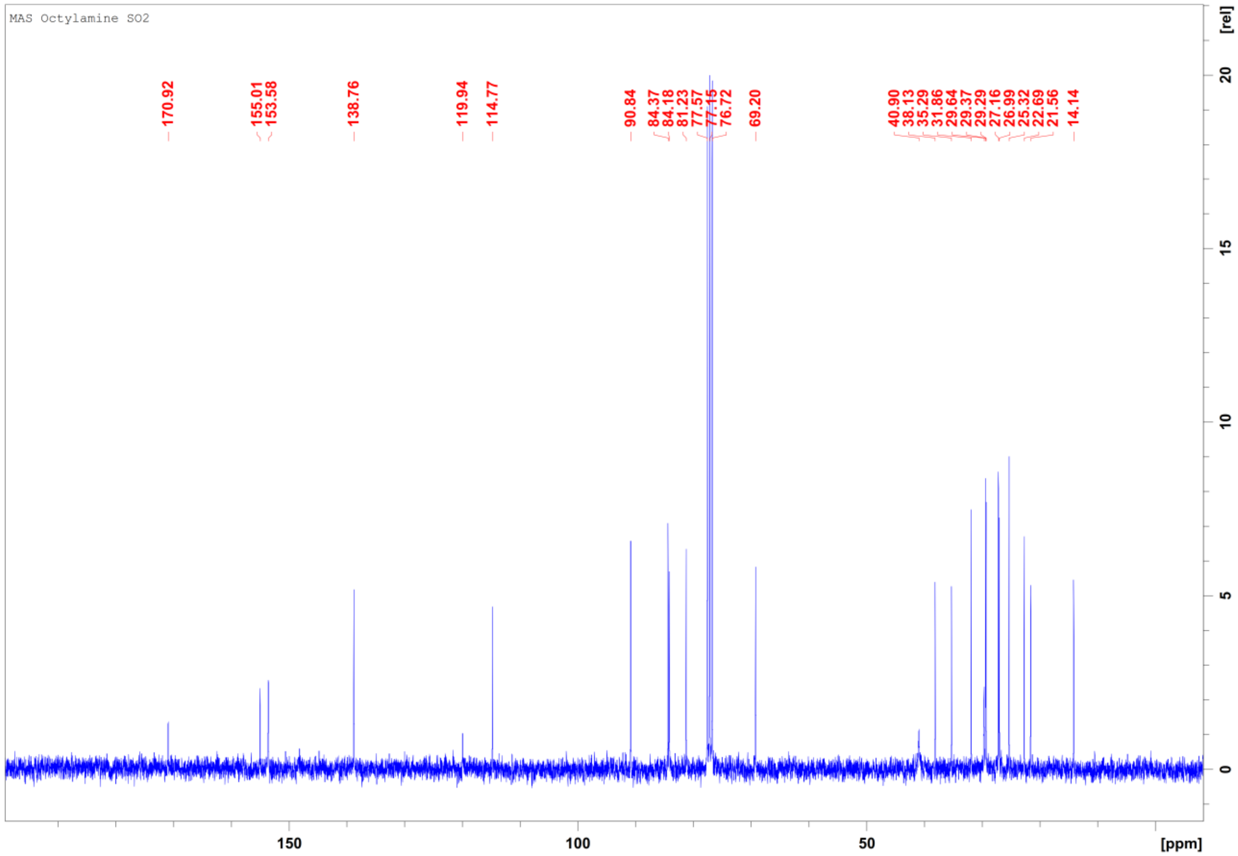
MAS Octylamine SO2
170.92
155.01
153.58
138.76
119.94
114.77
90.84
84.37
84.18
81.23
77.57
77.15
76.72
69.20
40.90
38.13
35.29
31.86
29.64
29.37
29.29
27.16
26.99
25.32
22.69
21.56
14.14
[rel]
[ppm]

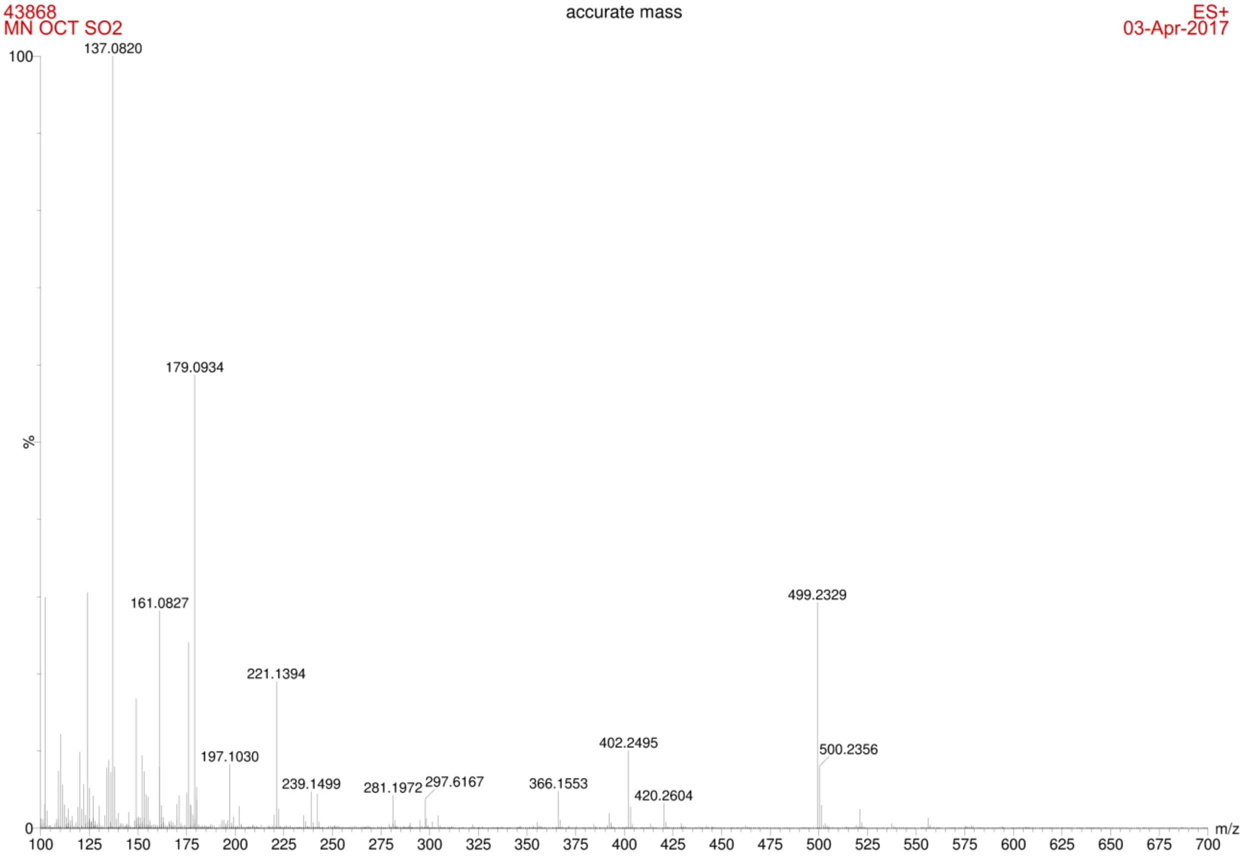
43868
MN OCT SO2
accurate mass
ES+
03-Apr-2017
137.0820
179.0934
161.0827
499.2329
221.1394
197.1030
402.2495
500.2356
239.1499
281.1972
297.6167
366.1553
420.2604
%
m/z

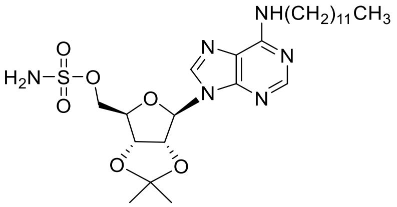

Exact Mass: 554.2887

2’,3’-isopropylidene-5’-O-sulfamoyl-N6-dodecyl-adenosine (4c)

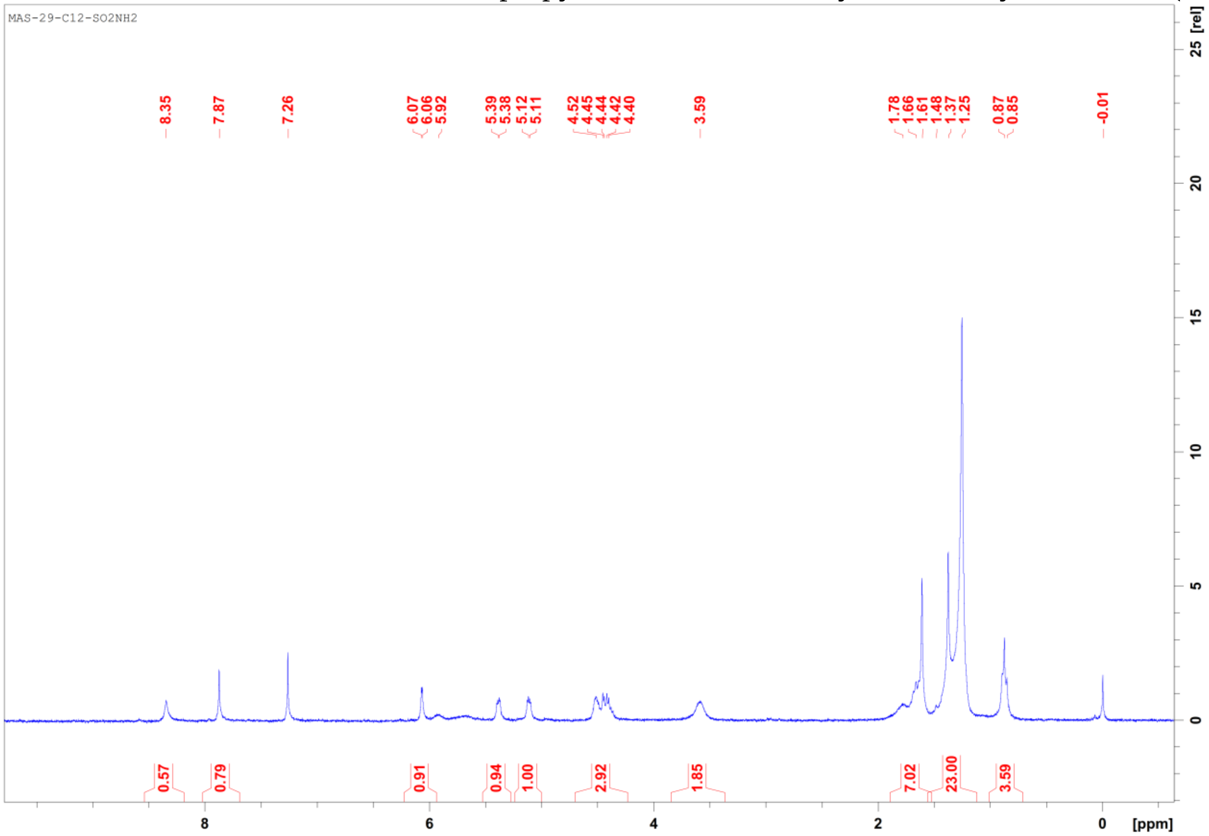

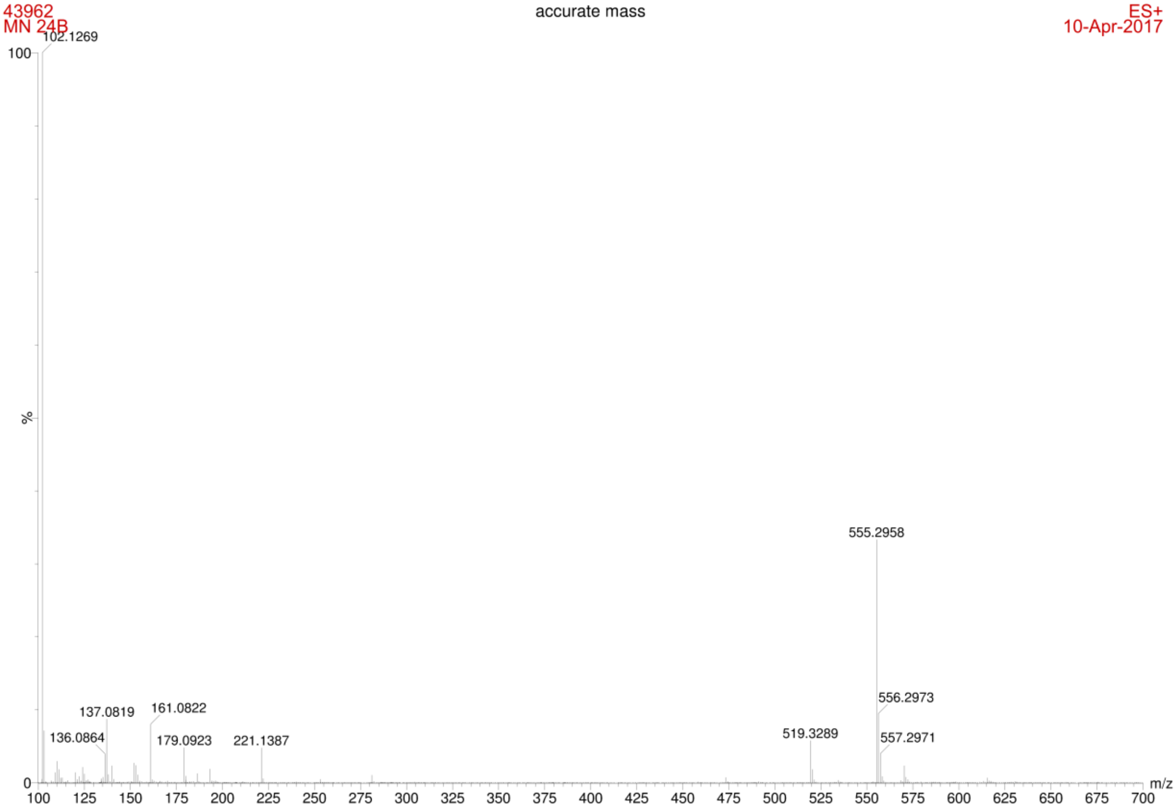

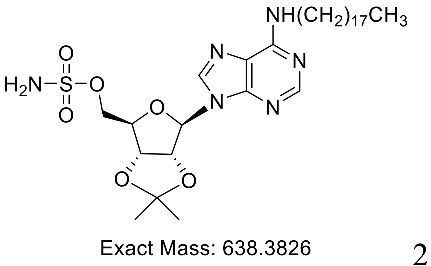

2’,3’-isopropylidene-5’-O-sulfamoyl-N6-octadecyl-adenosine (4d)

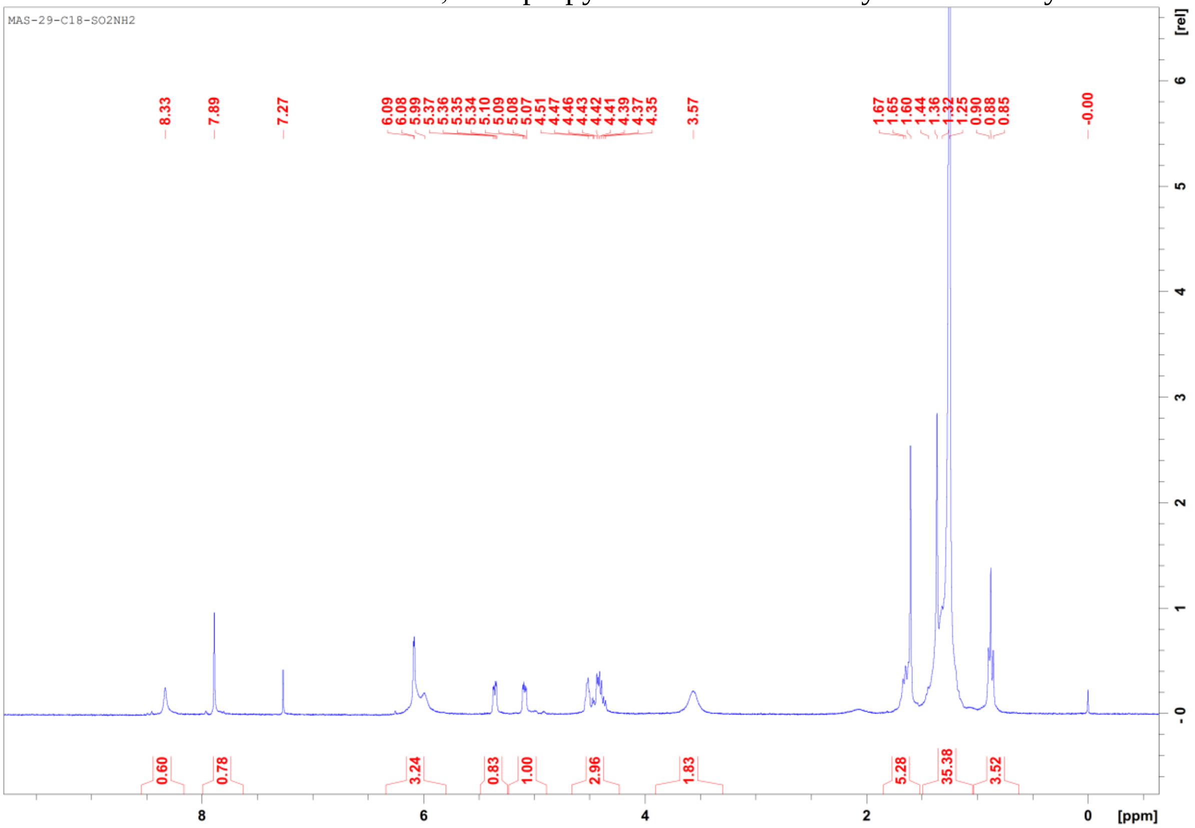

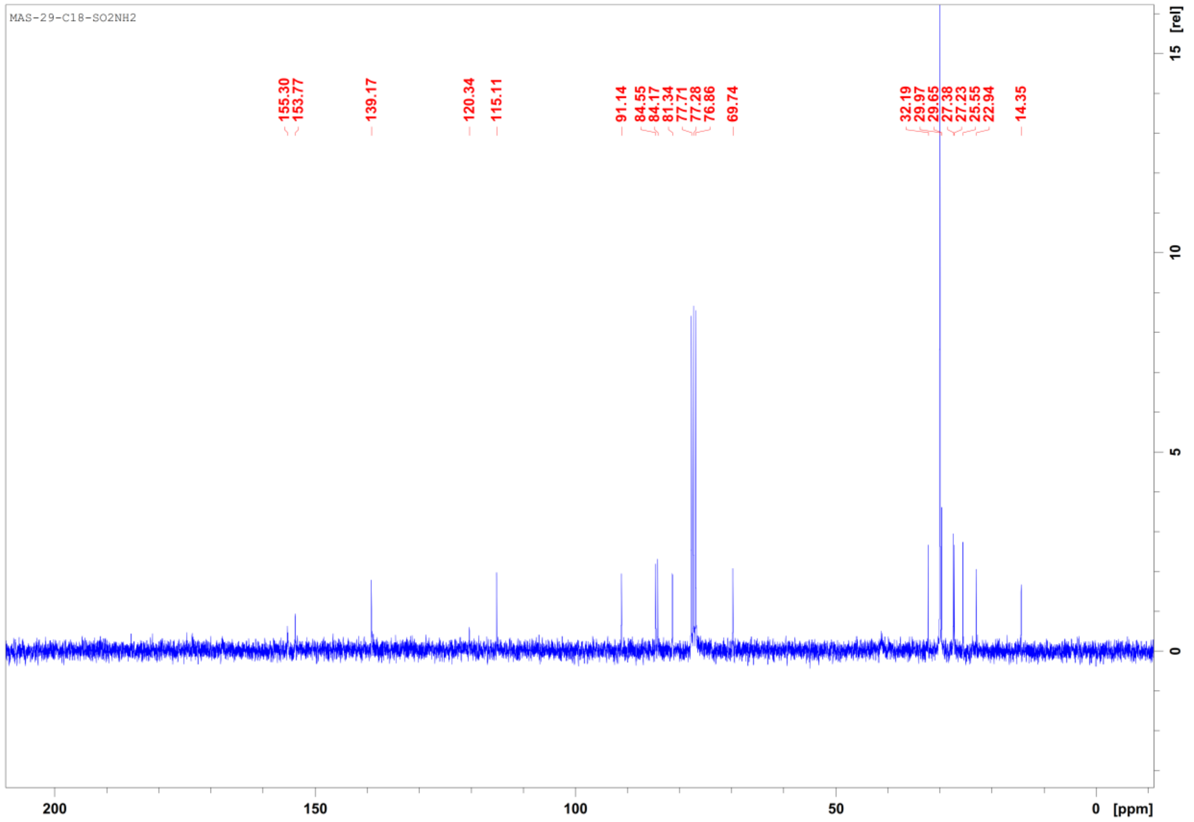

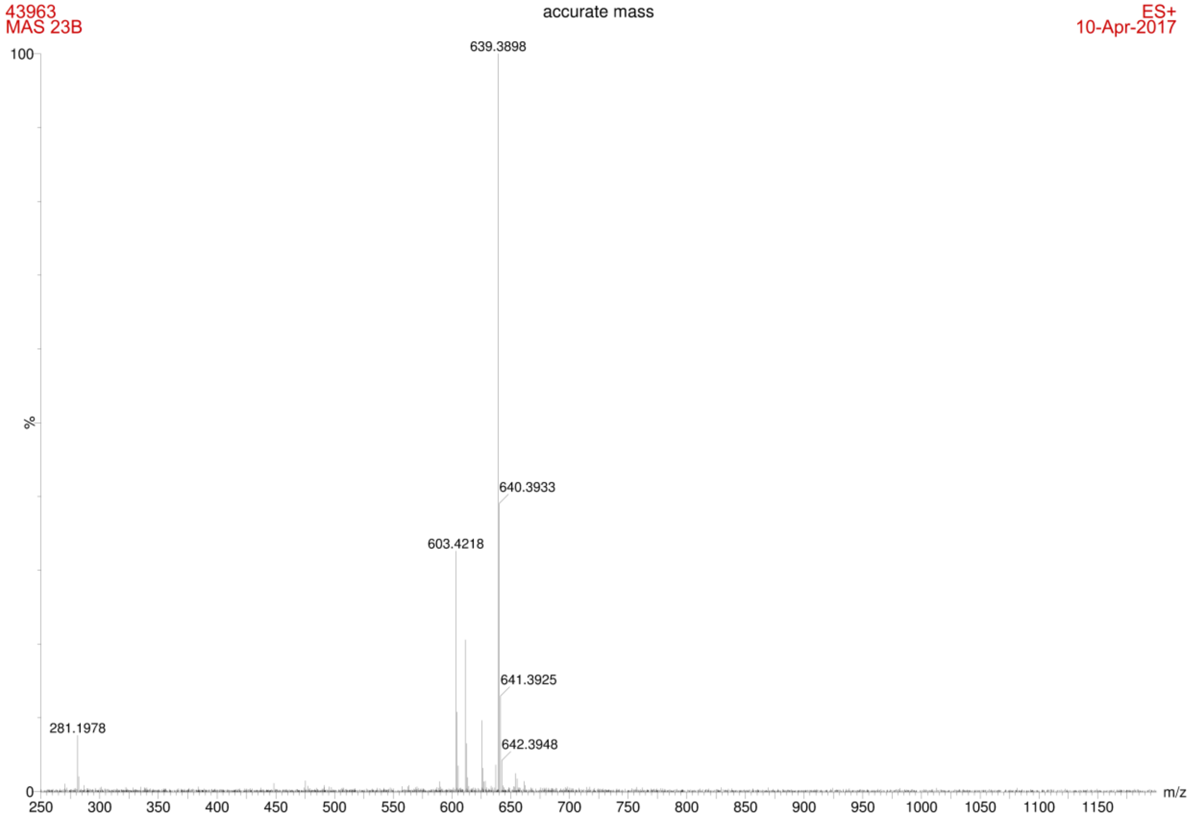

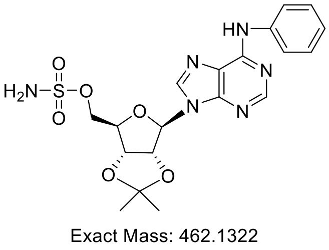

2’,3’-isopropylidene-5’-O-sulfamoyl-N6-phenyl-adenosine (4e)

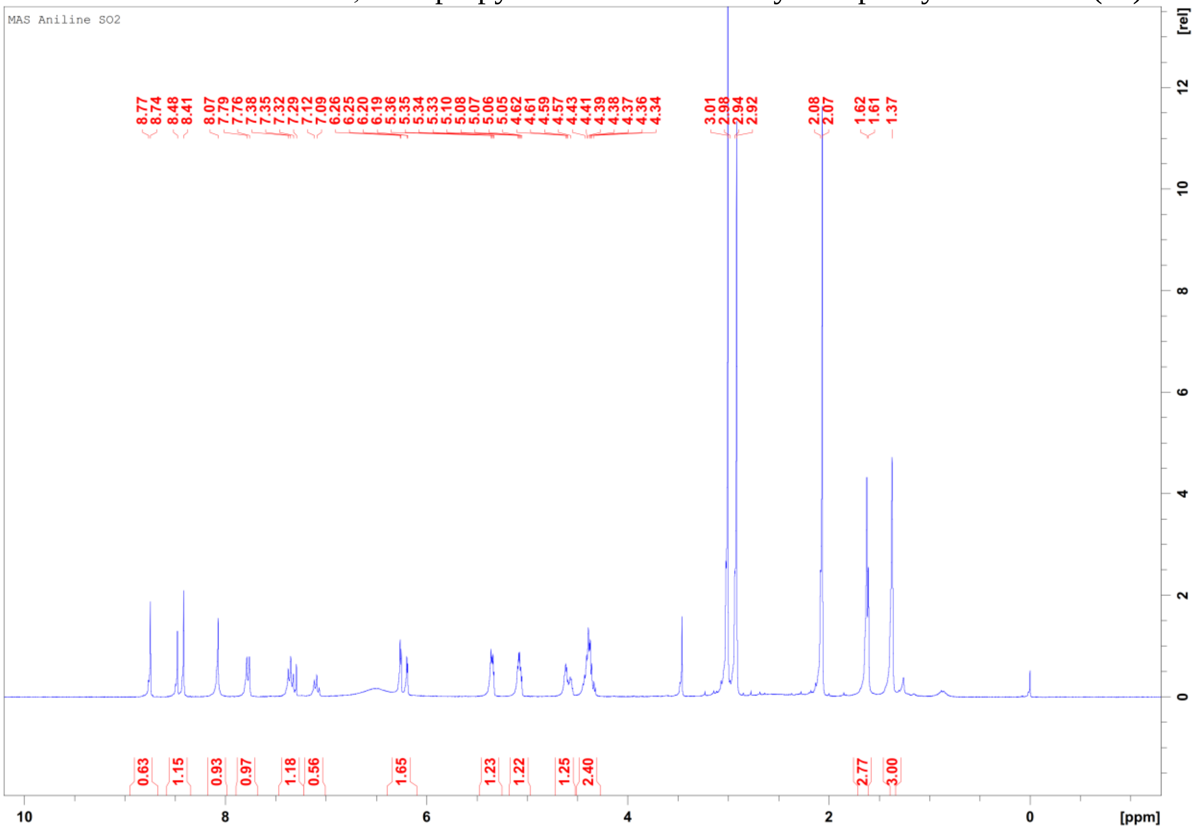

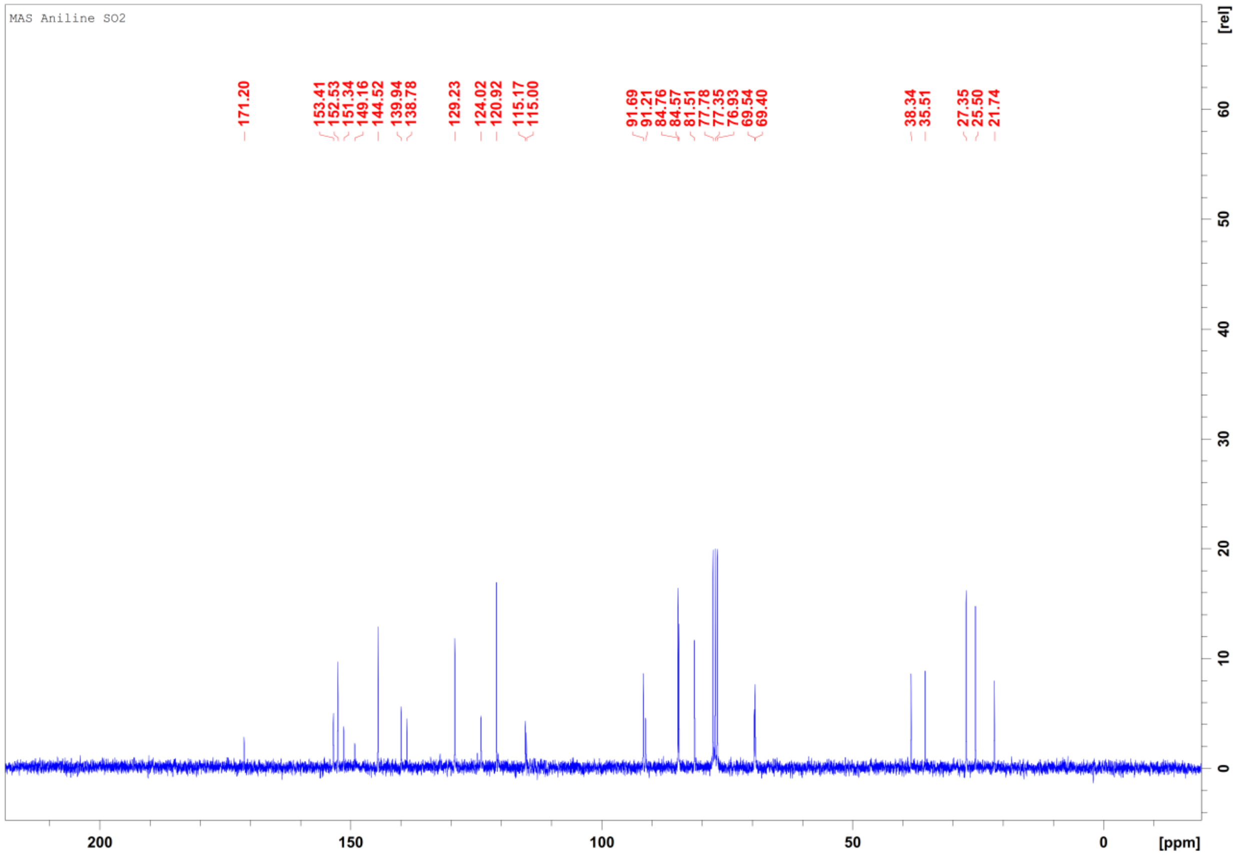
MAS Aniline SO2
171.20
153.41
152.53
151.34
149.16
144.52
139.94
138.78
129.23
124.02
120.92
115.17
115.00
91.69
91.21
84.76
84.57
81.51
77.78
77.35
76.93
69.54
69.40
38.34
35.51
27.35
25.50
21.74
[rel]
[ppm]

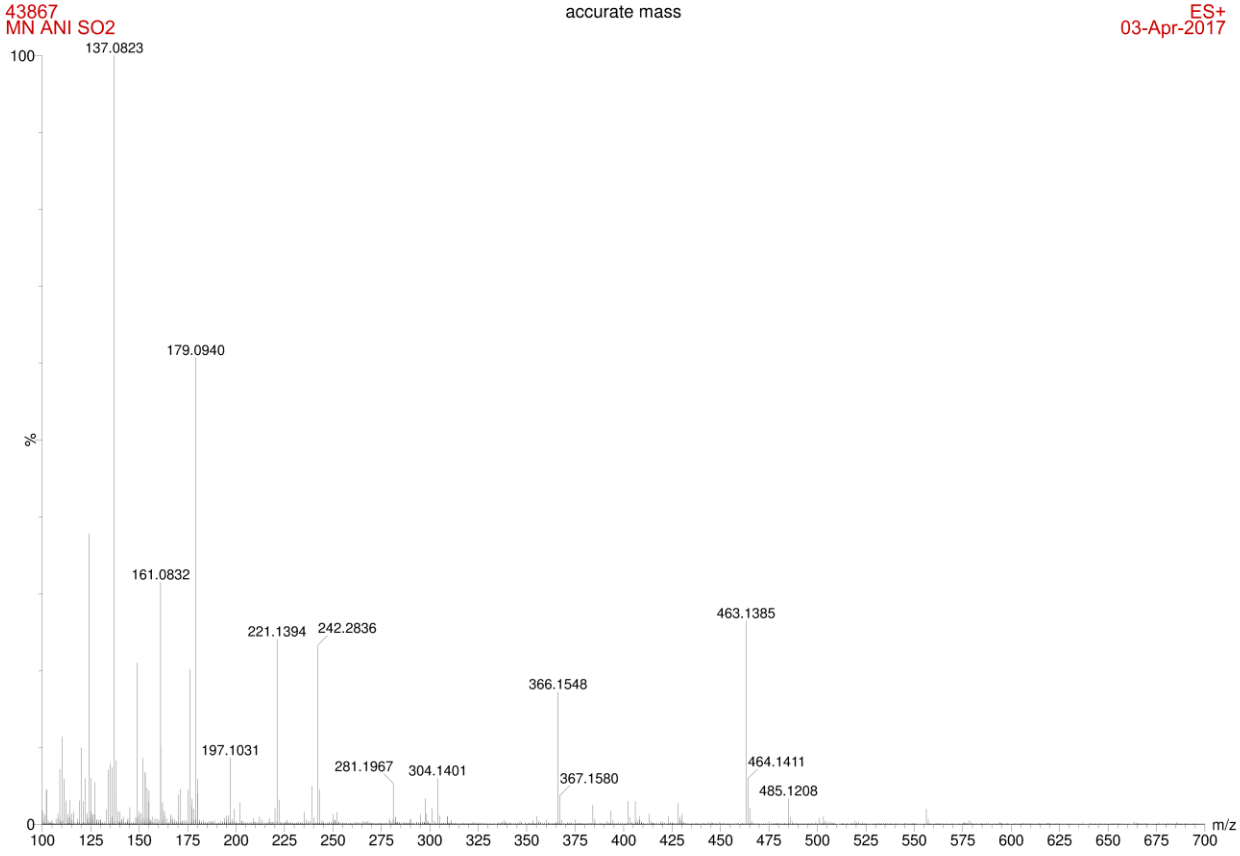
43867
MN ANI SO2
accurate mass
ES+
03-Apr-2017
137.0823
179.0940
161.0832
221.1394
242.2836
463.1385
366.1548
197.1031
281.1967
304.1401
367.1580
464.1411
485.1208
%
m/z

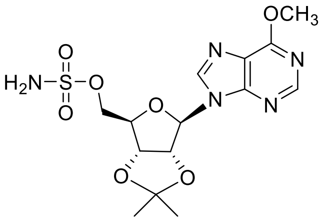

Exact Mass: 401.1005

2’, 3’-isopropylidene-5’-O-sulfamoyl-6-O-methyl-purine riboside (4f)

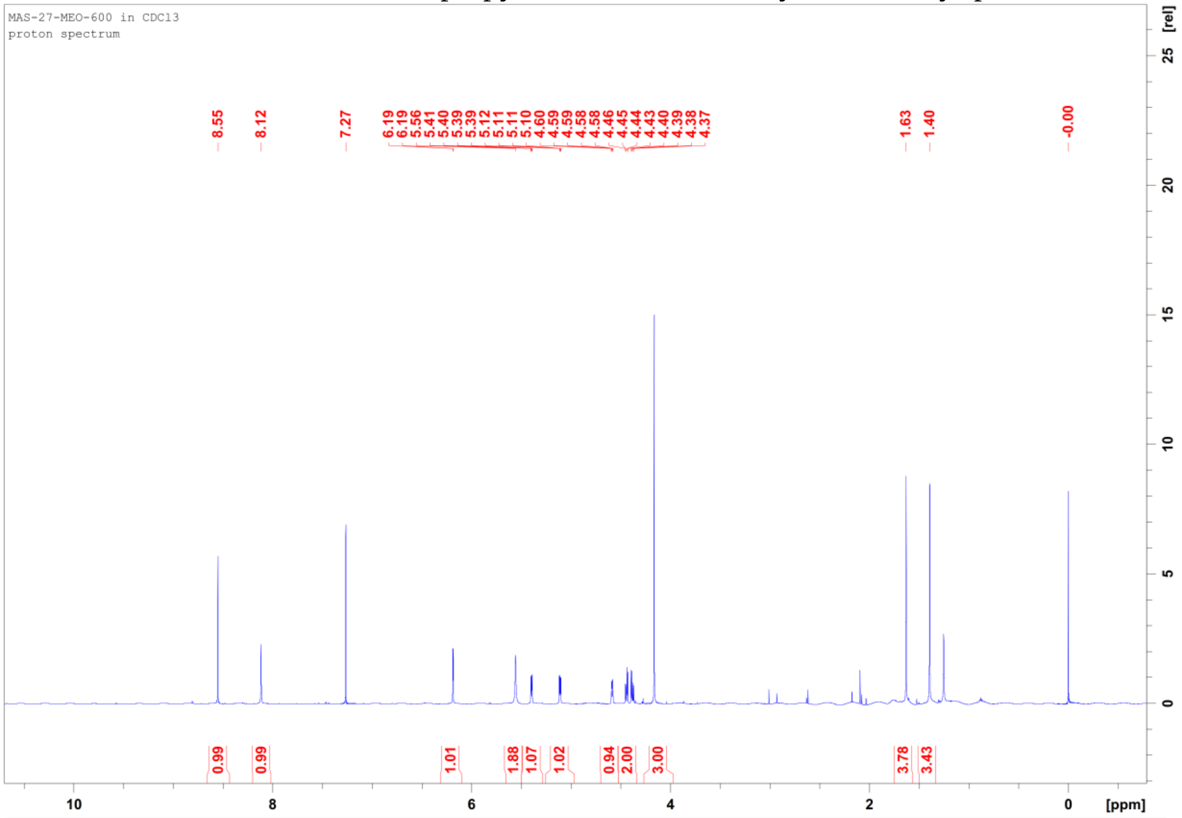

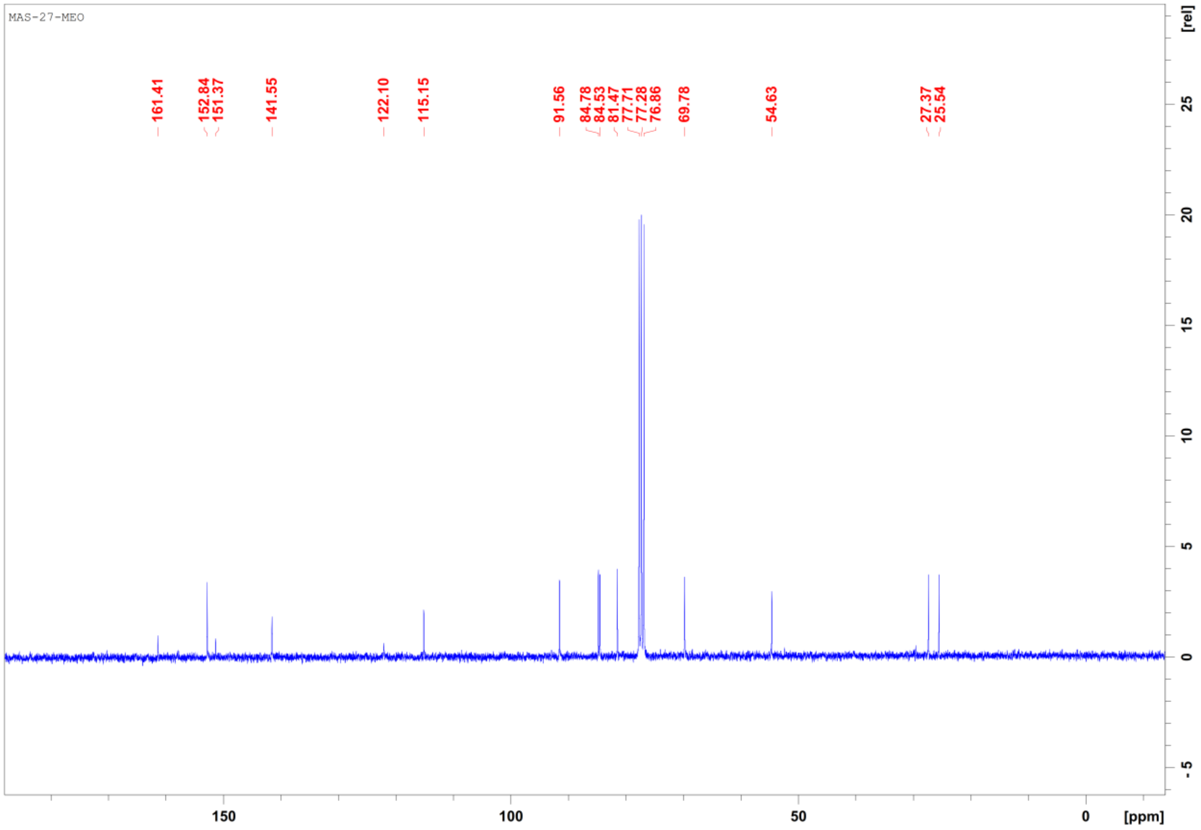

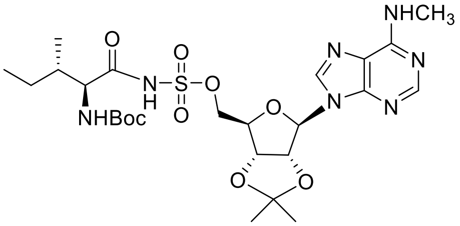

Exact Mass: 613.2530

2’,3’-isopropylidene-5’-O-(N-(Nα-Boc-L-isoleucyl))-sulfamoyl-N6-methyl-adenosine (5a)

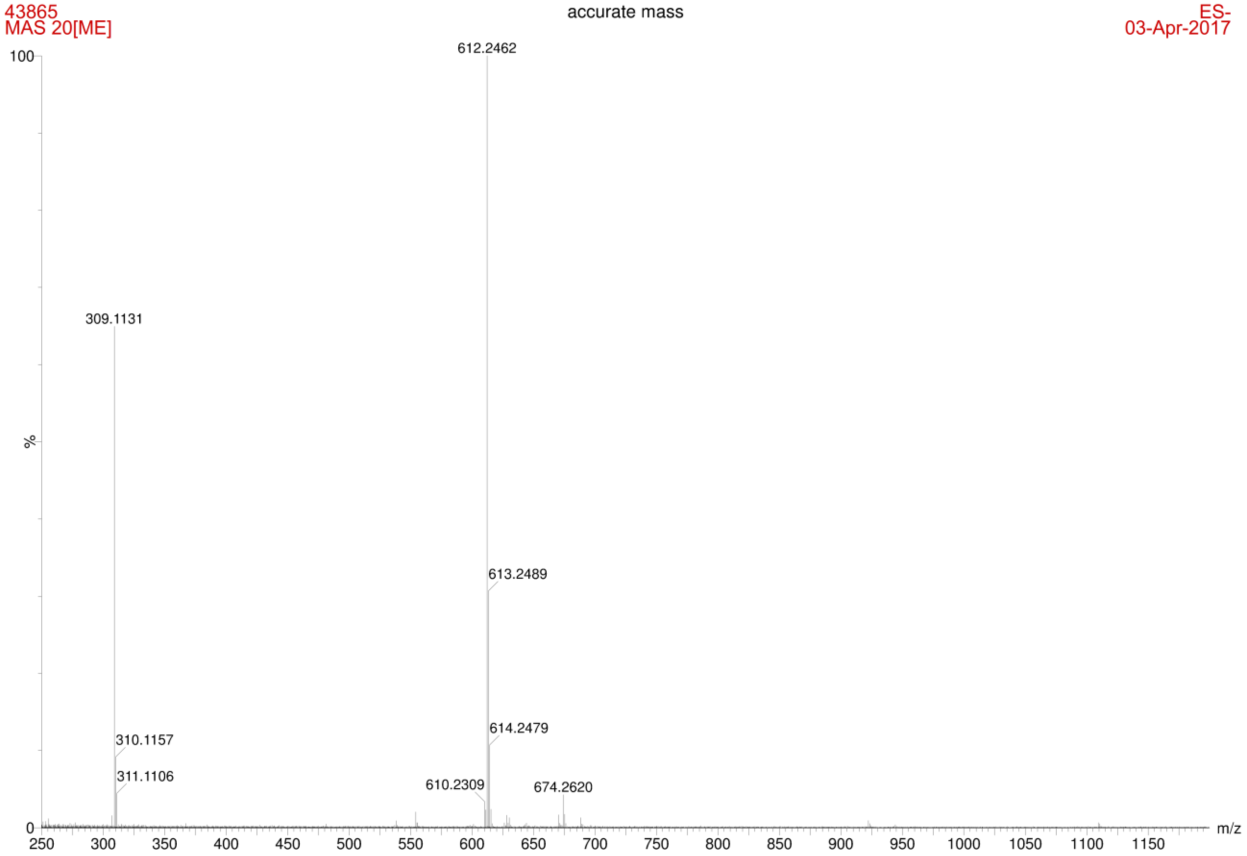

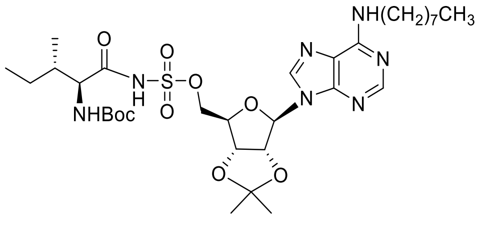

2’,3’-isopropylidene-5’-O-(N-(Nα-Boc-L-isoleucyl))-sulfamoyl-N6-octyl-adenosine (5b)

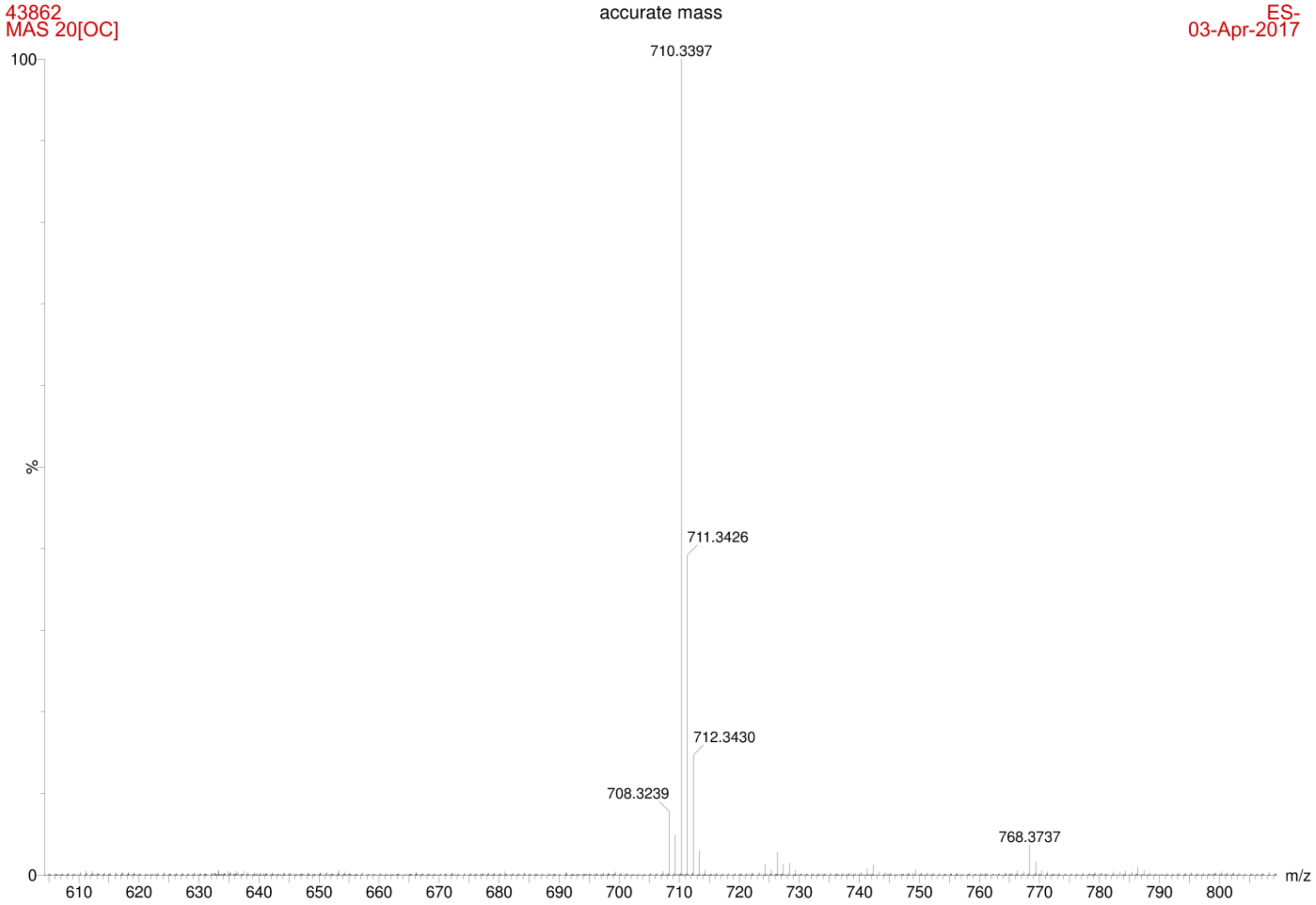

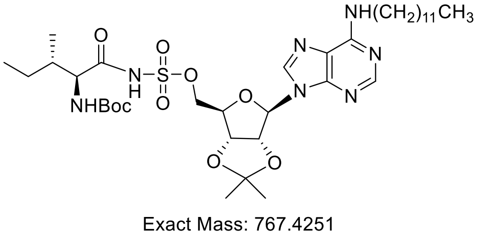

2’,3’-isopropylidene-5’-O-(N-(Nα-Boc-L-isoleucyl))-sulfamoyl-N6-dodecyl-adenosine (5c)

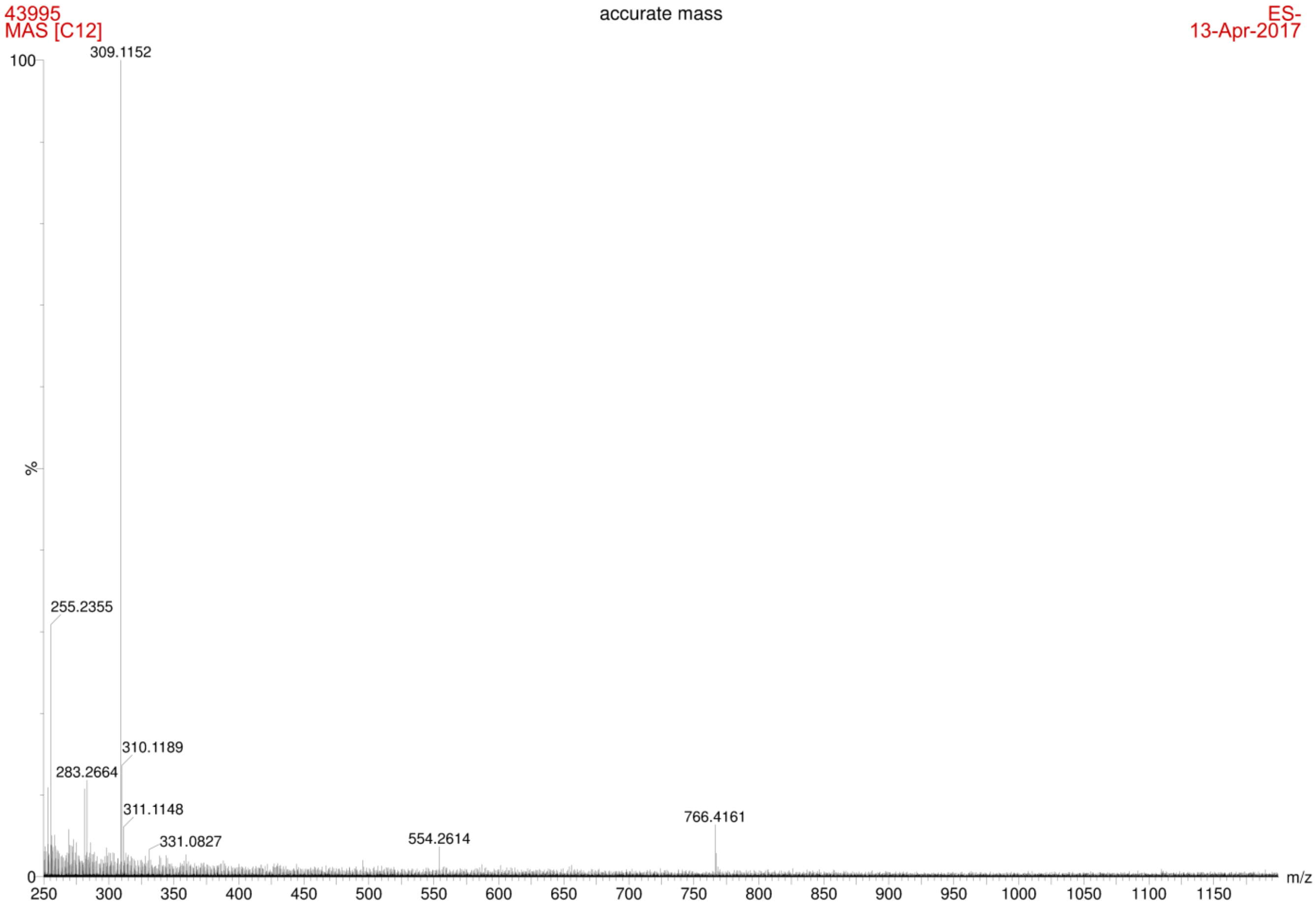

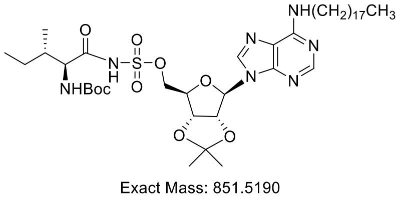

2’,3’-isopropylidene-5’-O-(N-(Nα-Boc-L-isoleucyl))-sulfamoyl-N6-octadecyl-adenosine (5d)

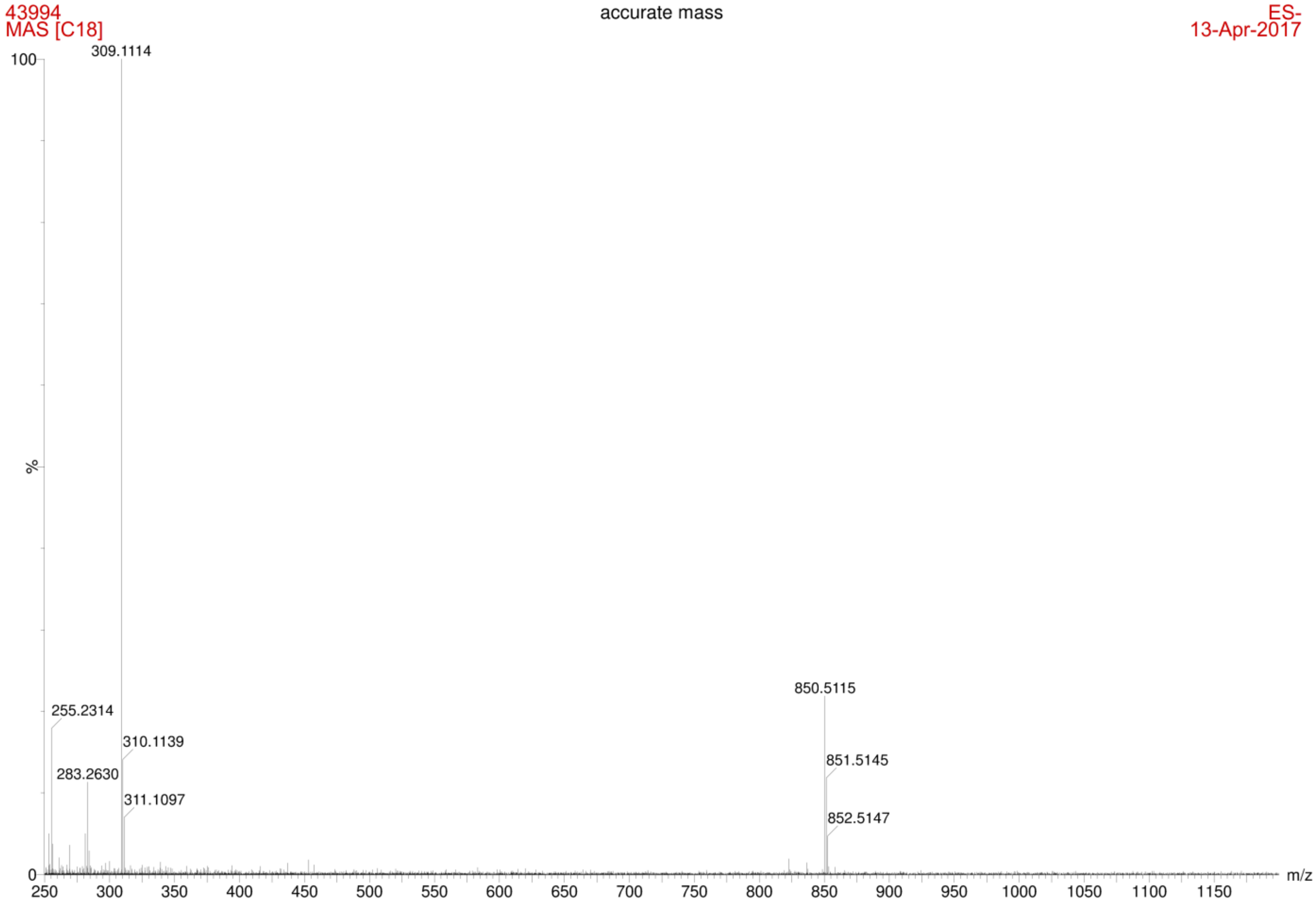

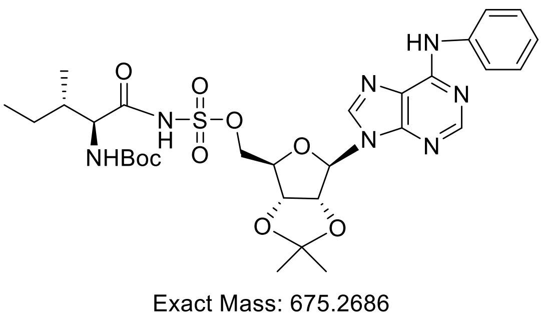

2’,3’-isopropylidene-5’-O-(N-(Nα-Boc-L-isoleucyl))-sulfamoyl-N6-phenyl-adenosine (5e)

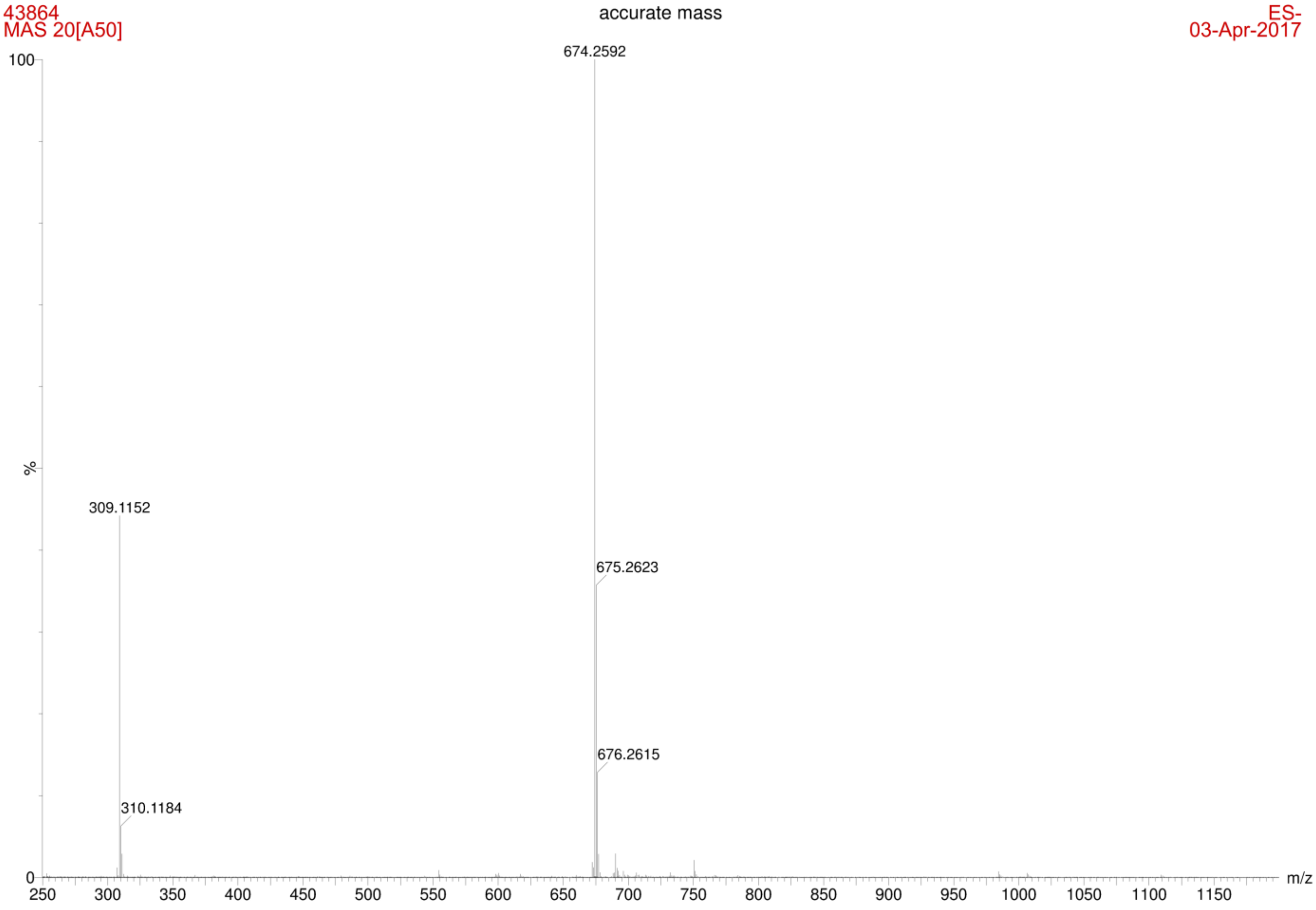

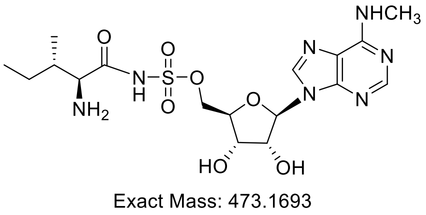

5’-*O*-(*N*-L-isoleucyl)-sulfamoyl-*N*^{6}-methyl-adenosine (6a)

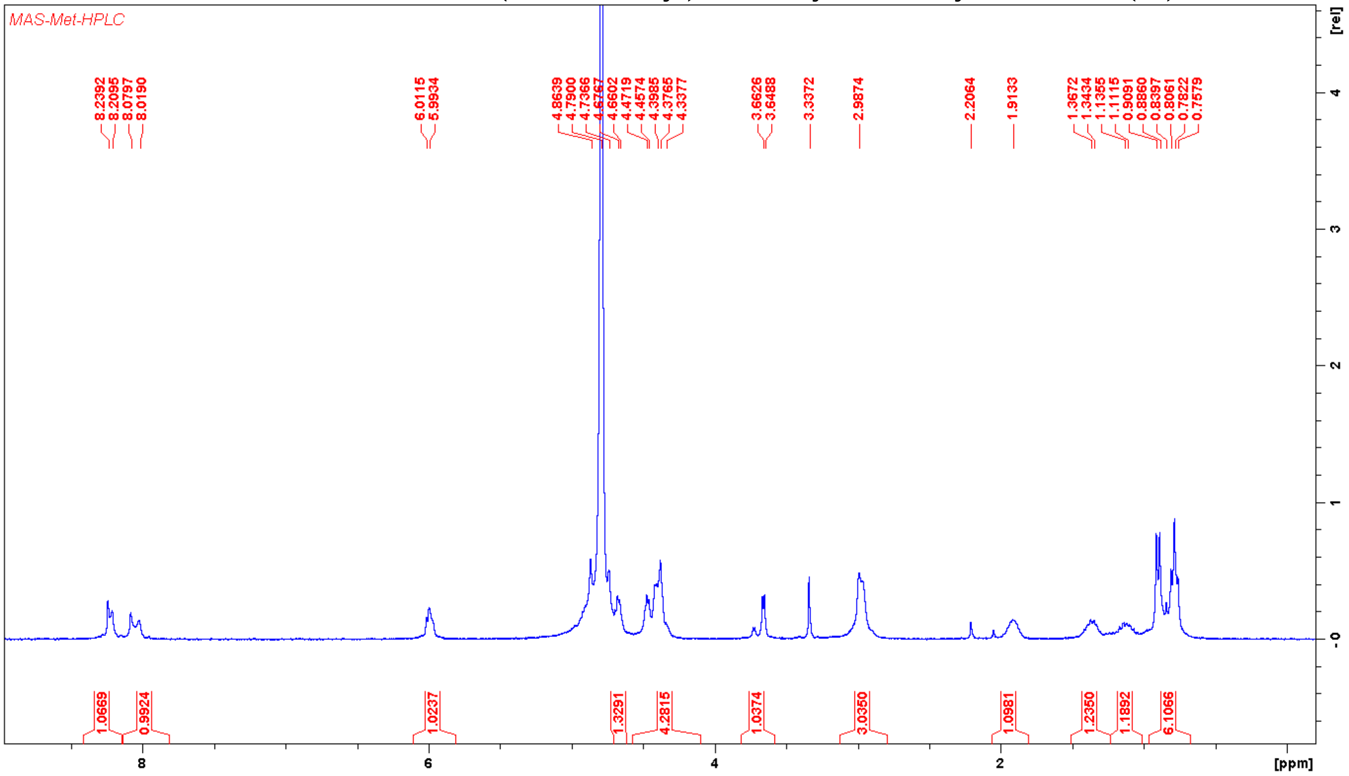

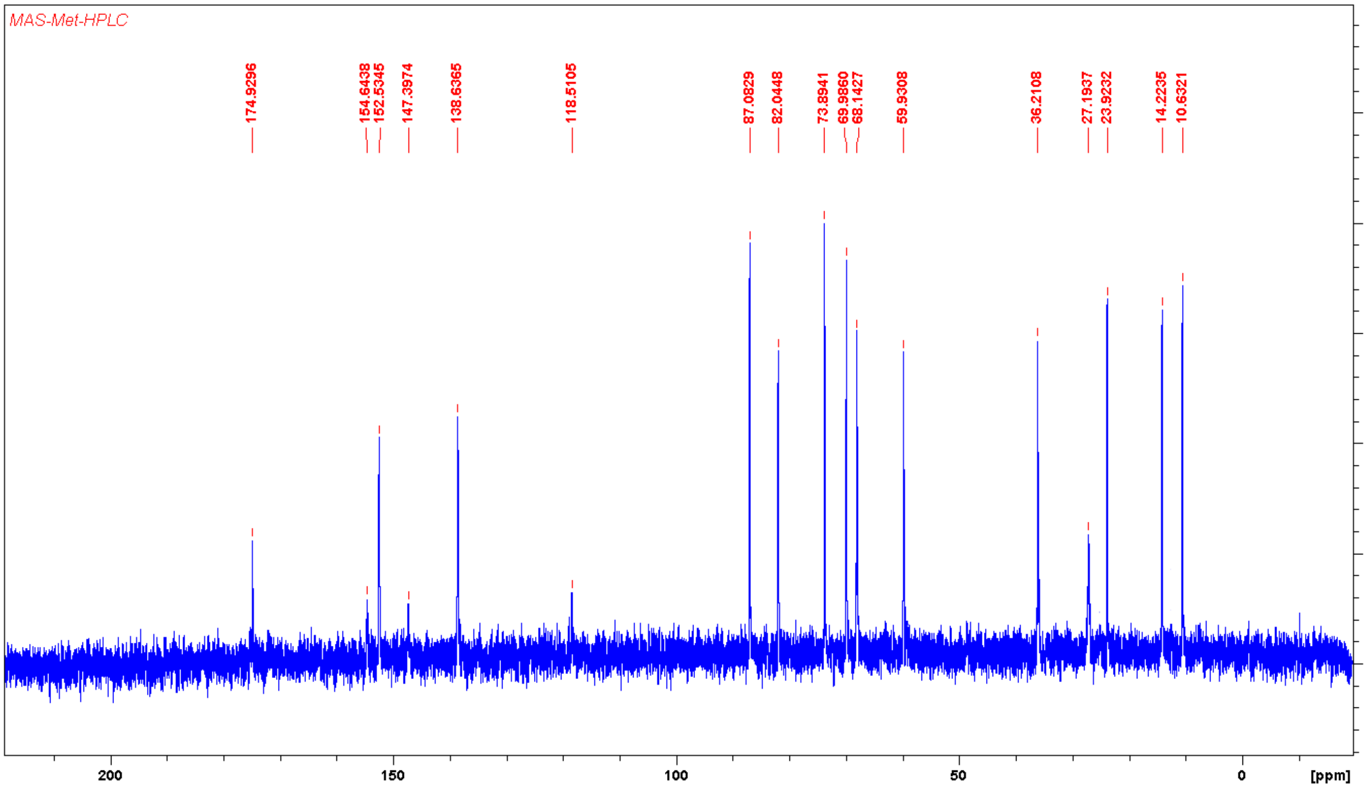

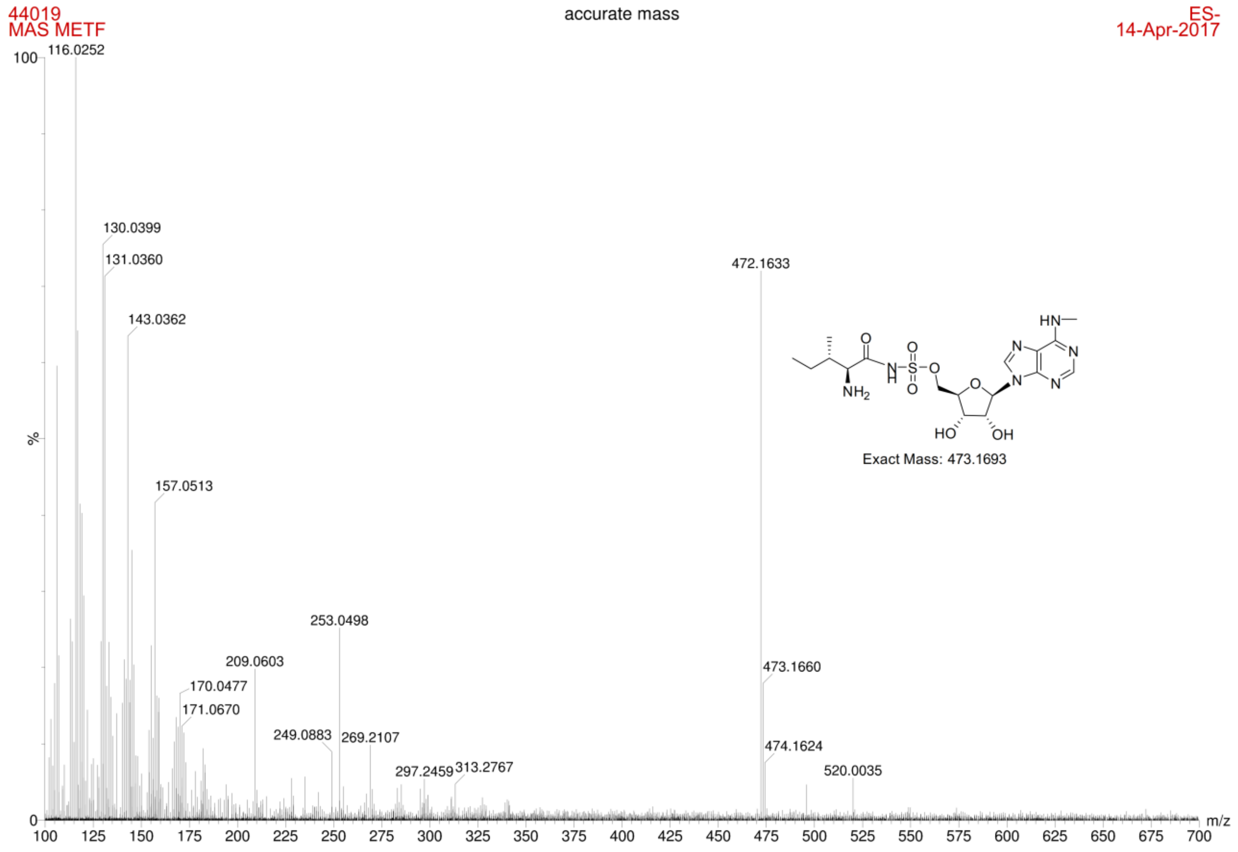

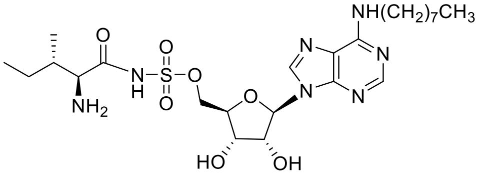

Exact Mass: 571.2788

5’-O-(N-L-isoleucyl)-sulfamoyl-N6-octyl-adenosine (6b)

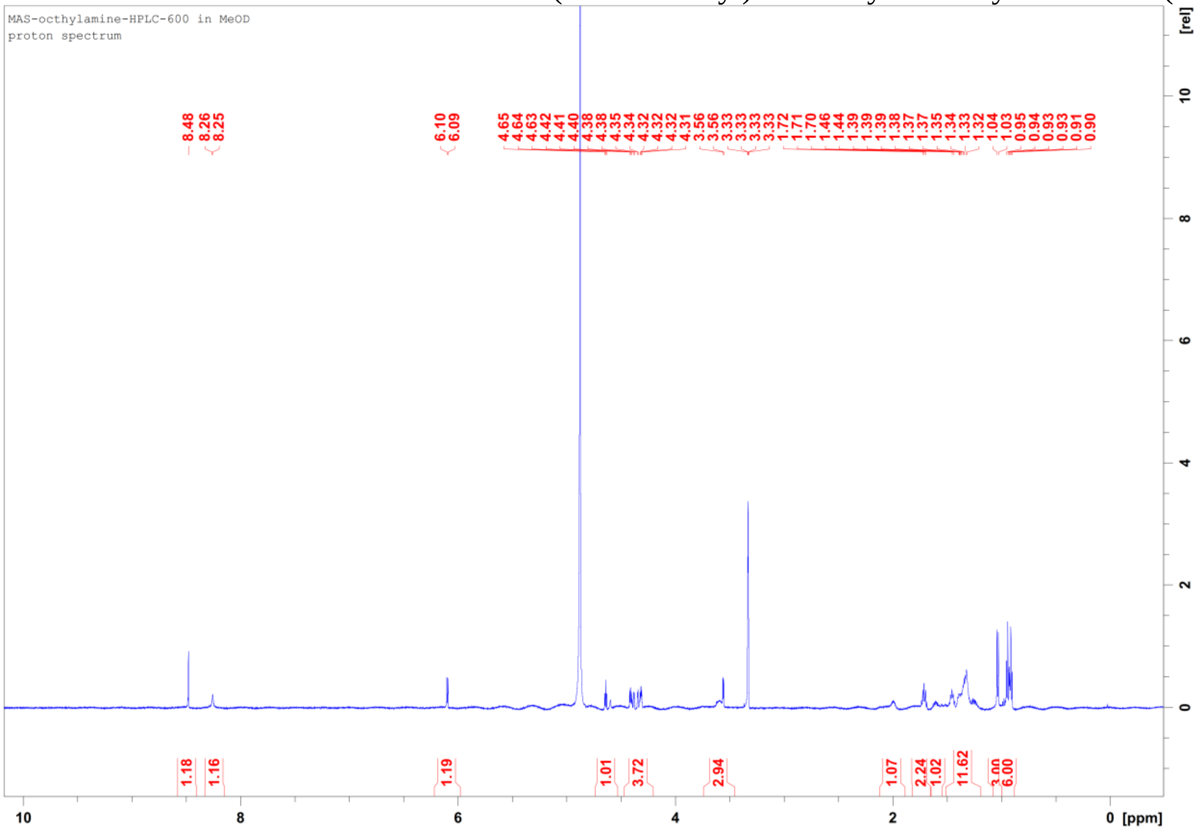

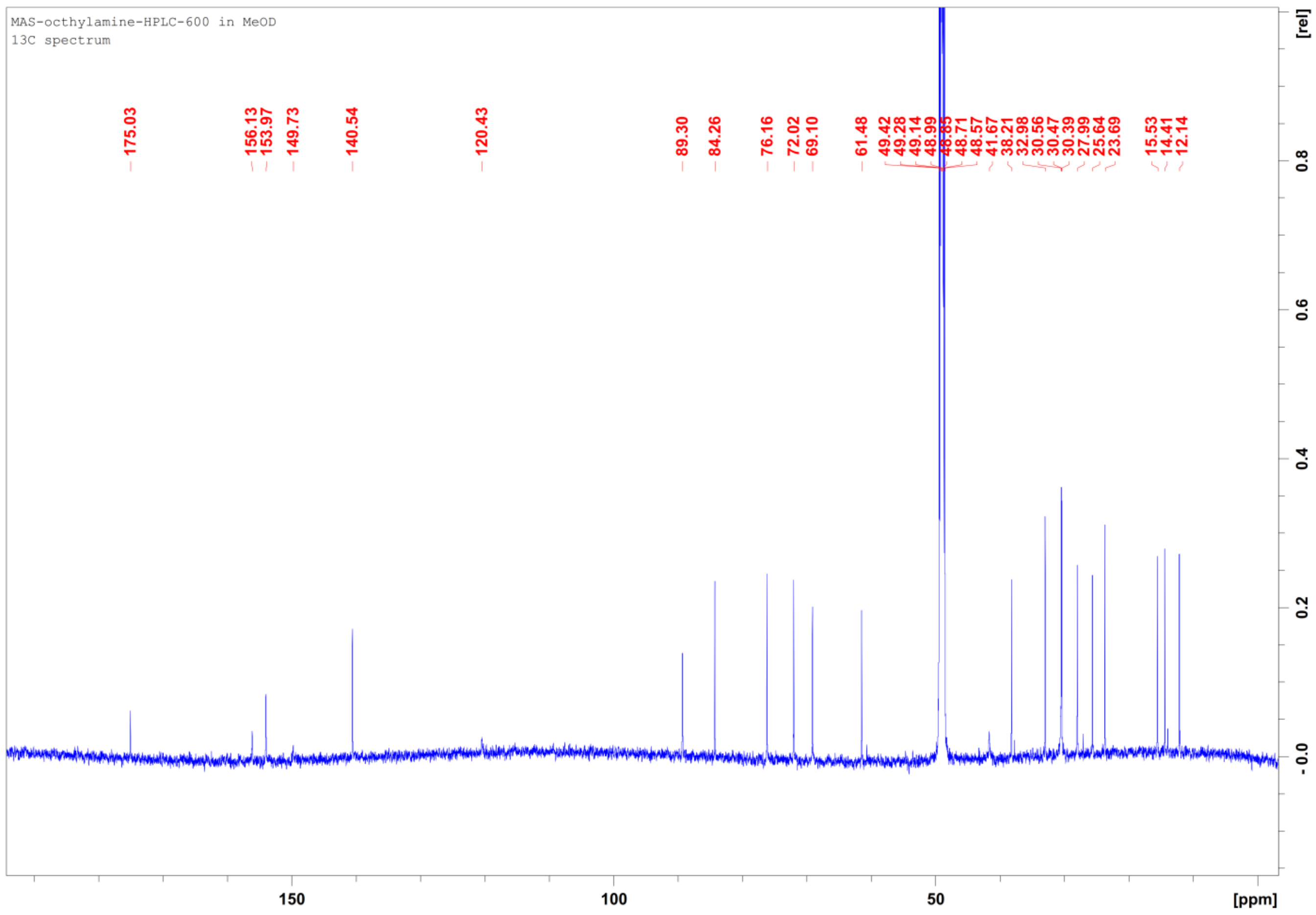
MAS-octhylamine-HPLC-600 in MeOD
13C spectrum
175.03
156.13
153.97
149.73
140.54
120.43
89.30
84.26
76.16
72.02
69.10
61.48
49.42
49.28
49.14
48.99
48.85
48.71
48.57
41.67
38.21
32.98
30.56
30.47
30.39
27.99
25.64
23.69
15.53
14.41
12.14
[rel]
0.8
0.6
0.4
0.2
-0.0
150
100
50
[ppm]

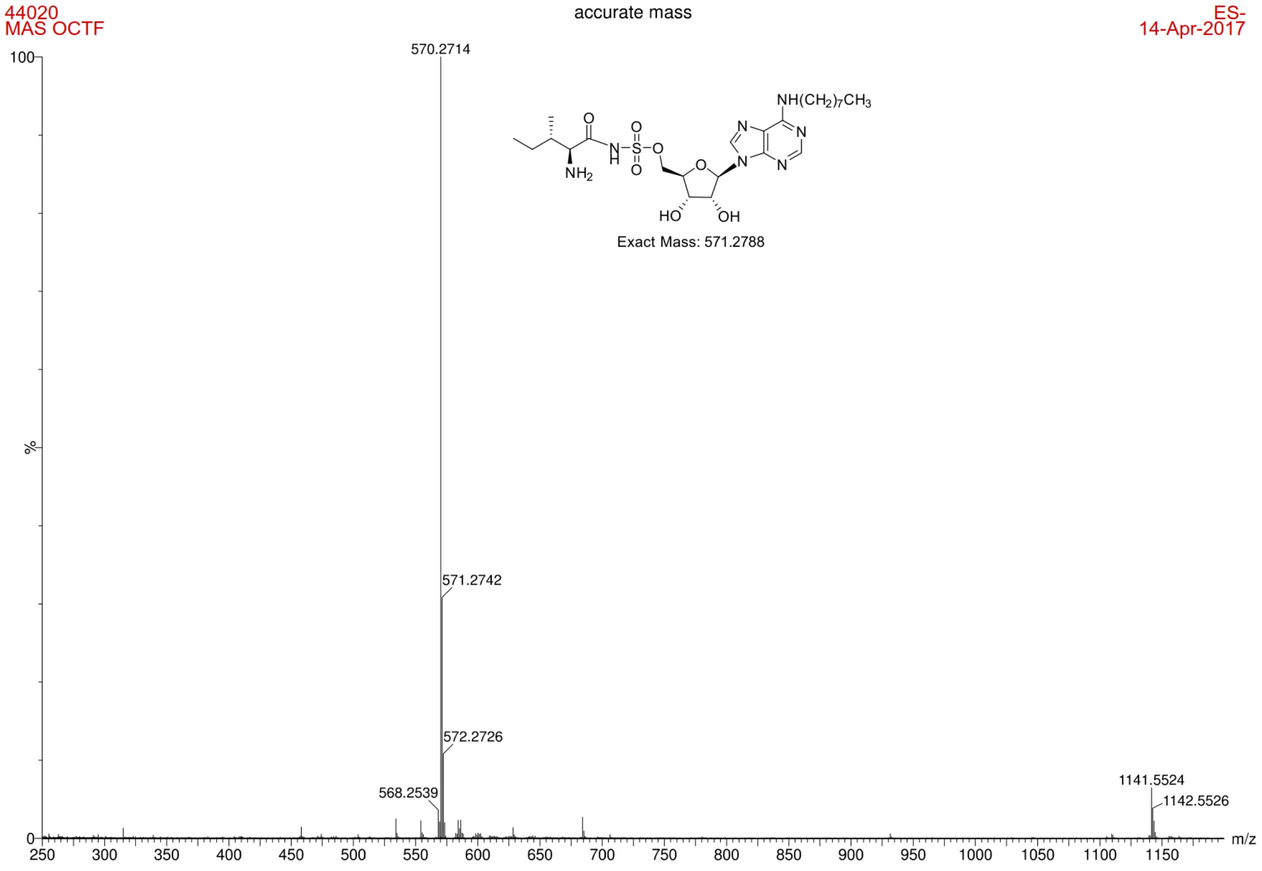
44020
MAS OCTF
accurate mass
ES-
14-Apr-2017
100
%
0
570.2714
571.2742
572.2726
568.2539
1141.5524
1142.5526
NH(CH2)7CH3
NH2
HO
OH
Exact Mass: 571.2788
m/z
250
300
350
400
450
500
550
600
650
700
750
800
850
900
950
1000
1050
1100
1150

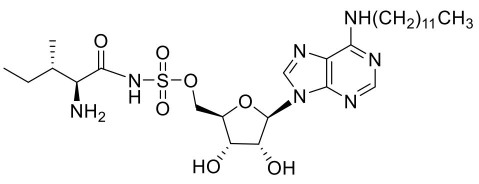

Exact Mass: 627.3414

5'-O-(N-L-isoleucyl)-sulfamoyl-N6-dodecyl-adenosine (6c)

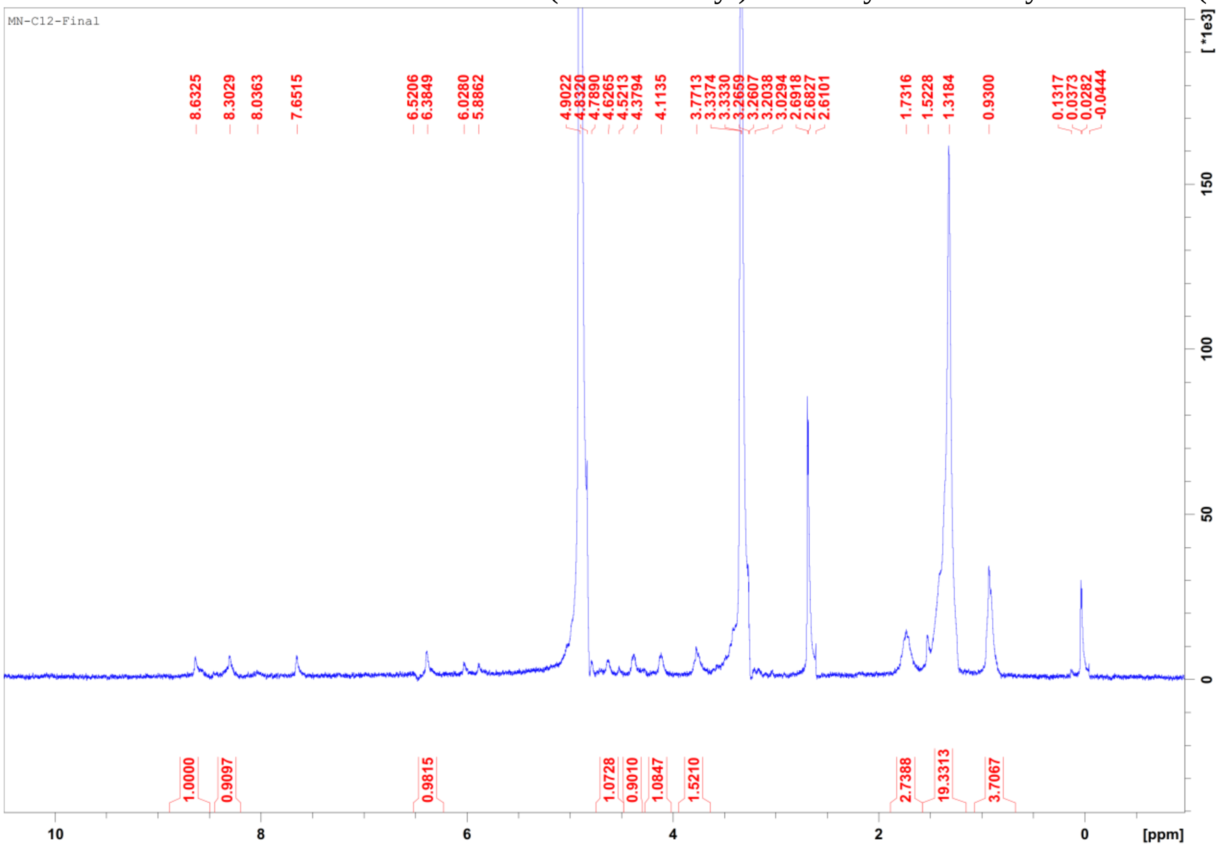

accurate mass

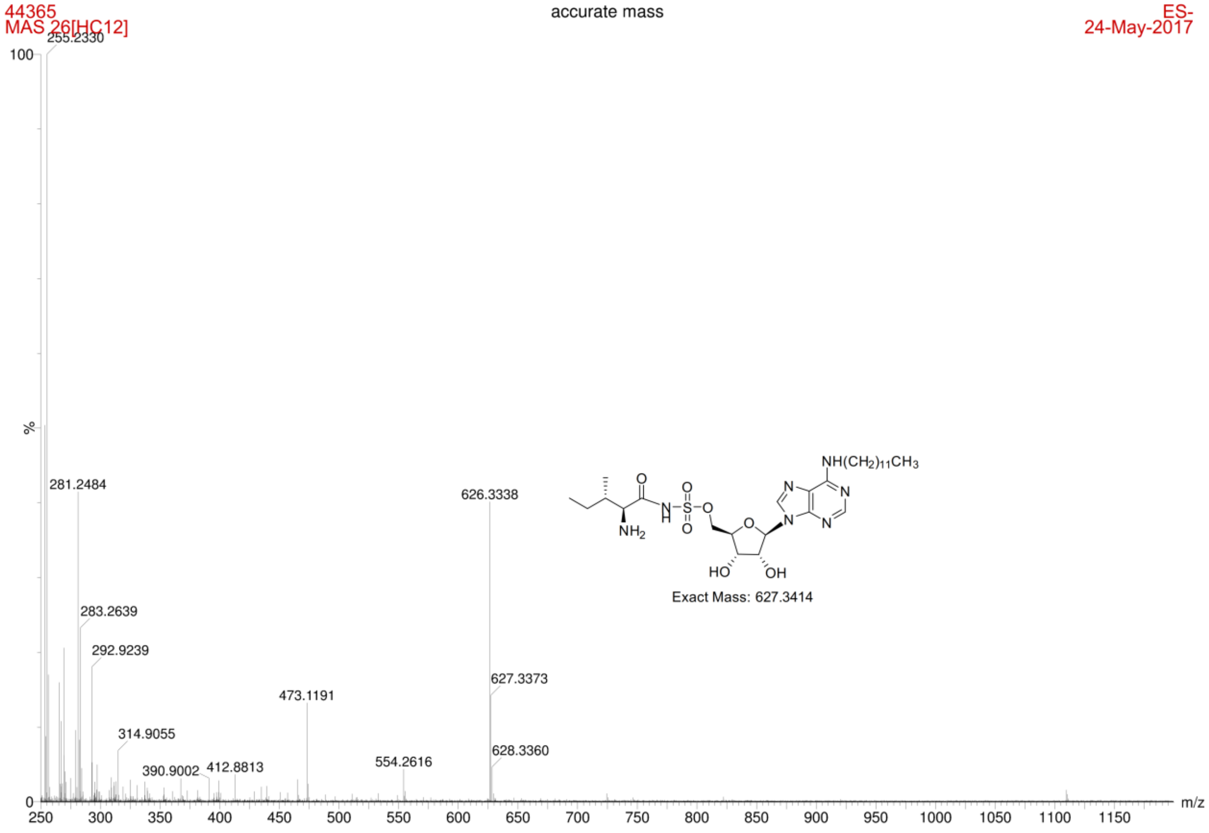

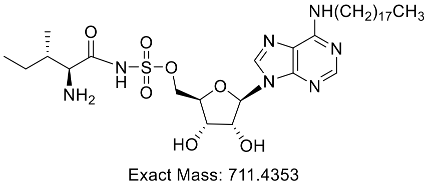

5’-O-(N-L-isoleucyl)-sulfamoyl-N6-octadecyl-adenosine (6d)

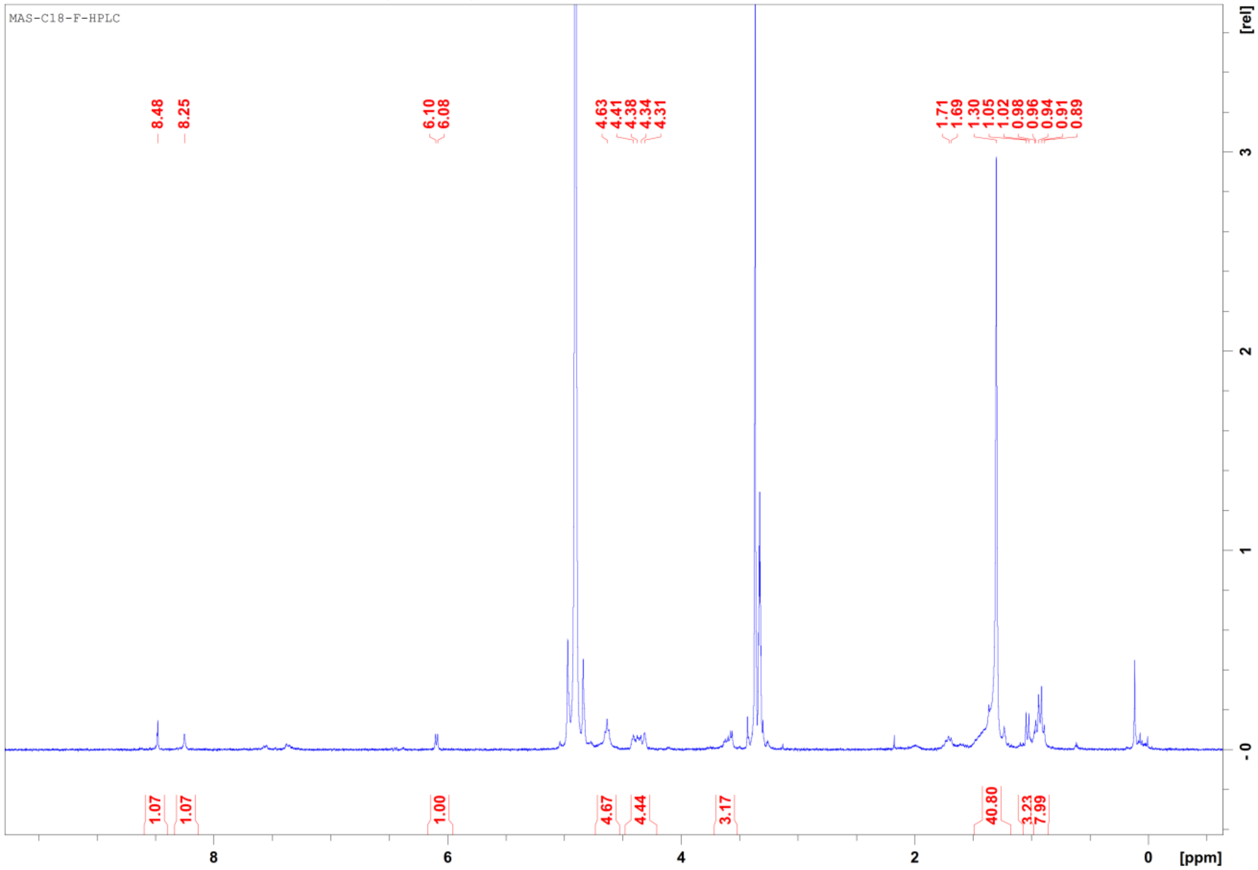

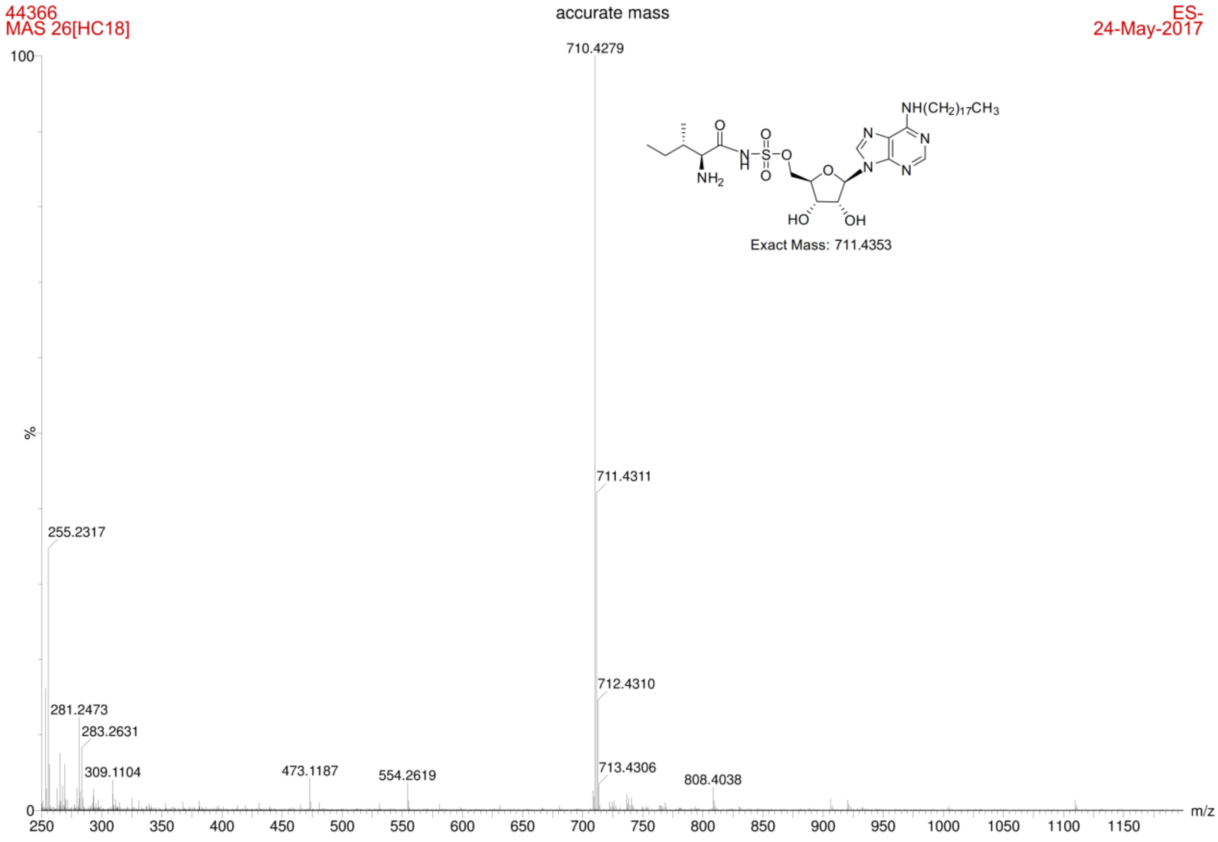

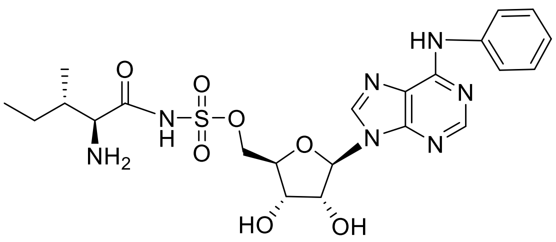

Exact Mass: 535.1849

5’-*O*-(*N*-L-isoleucyl)-sulfamoyl-N^6-phenyl-adenosine (6e)

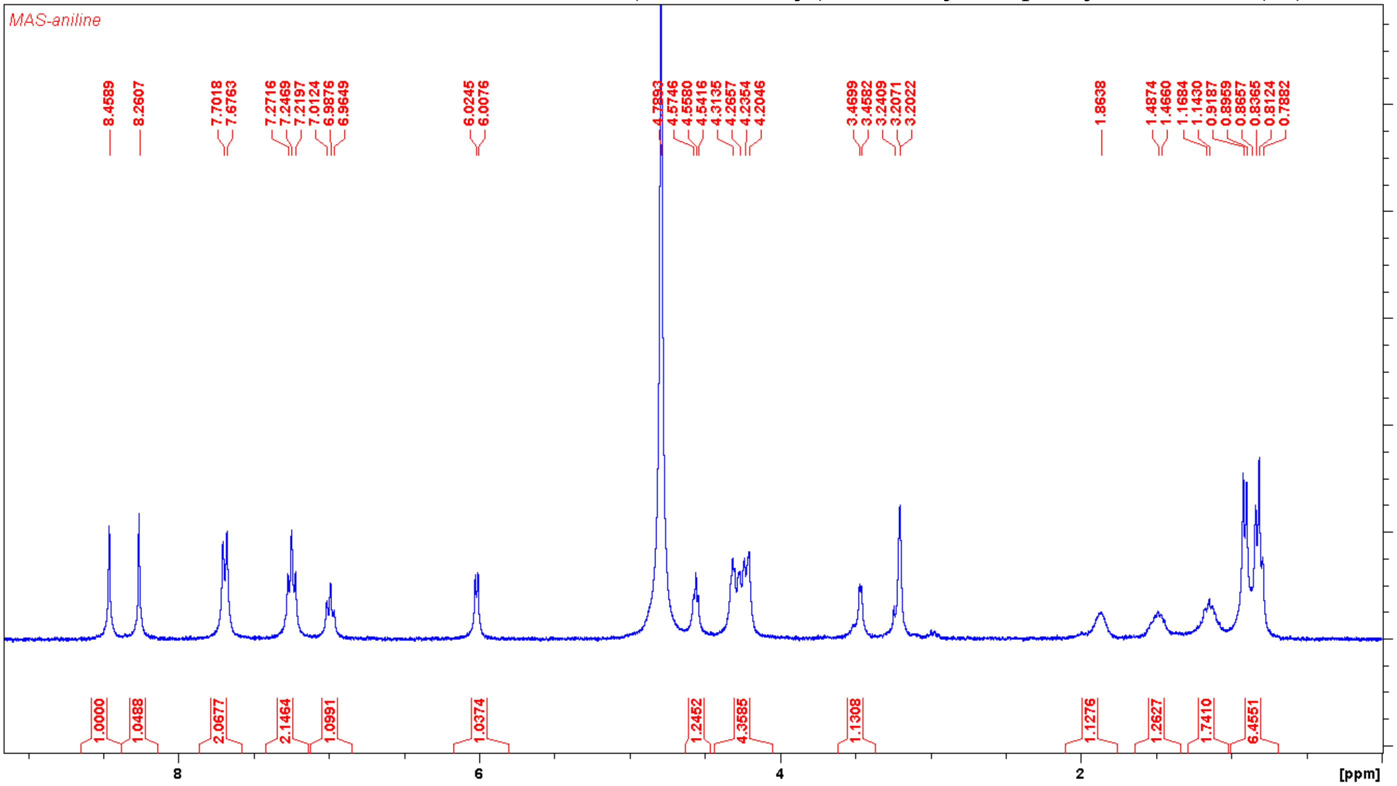

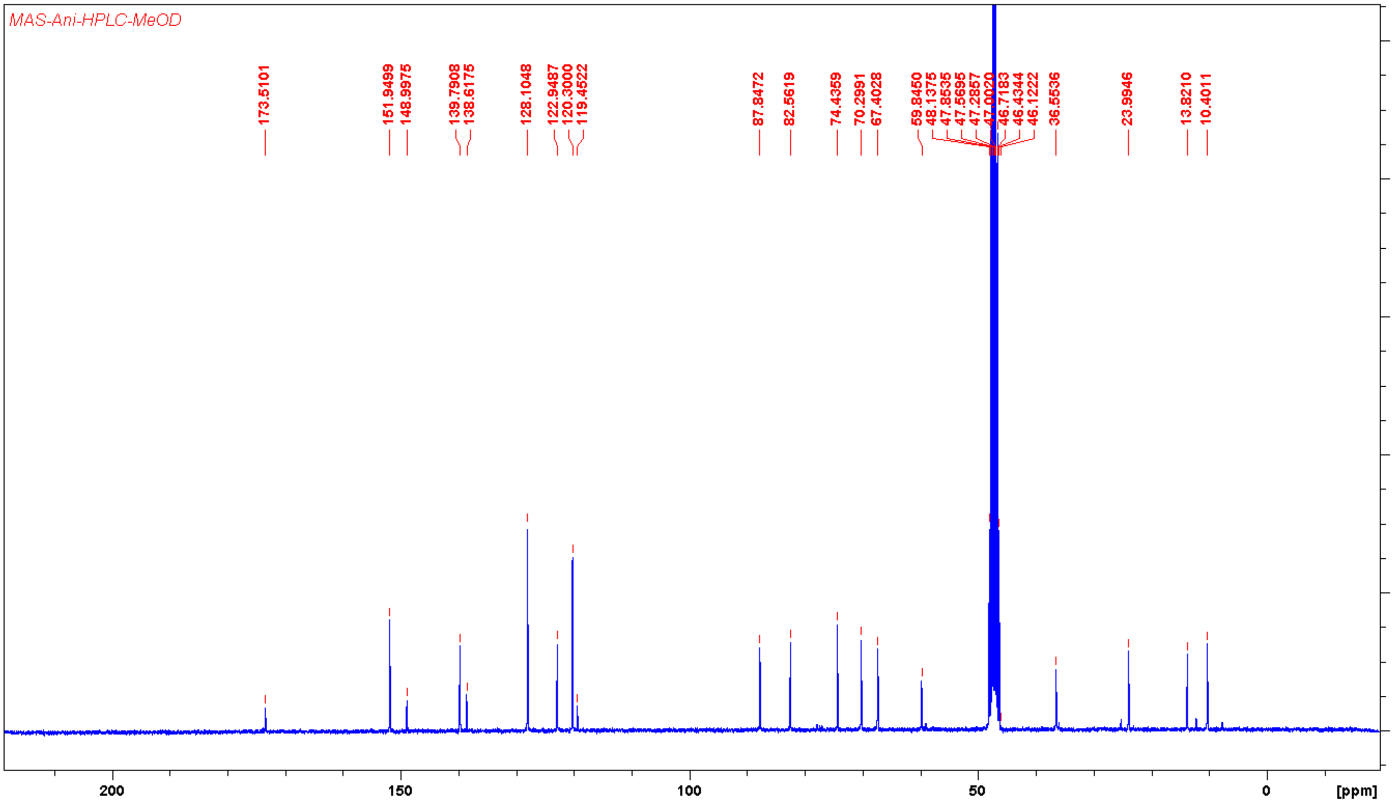

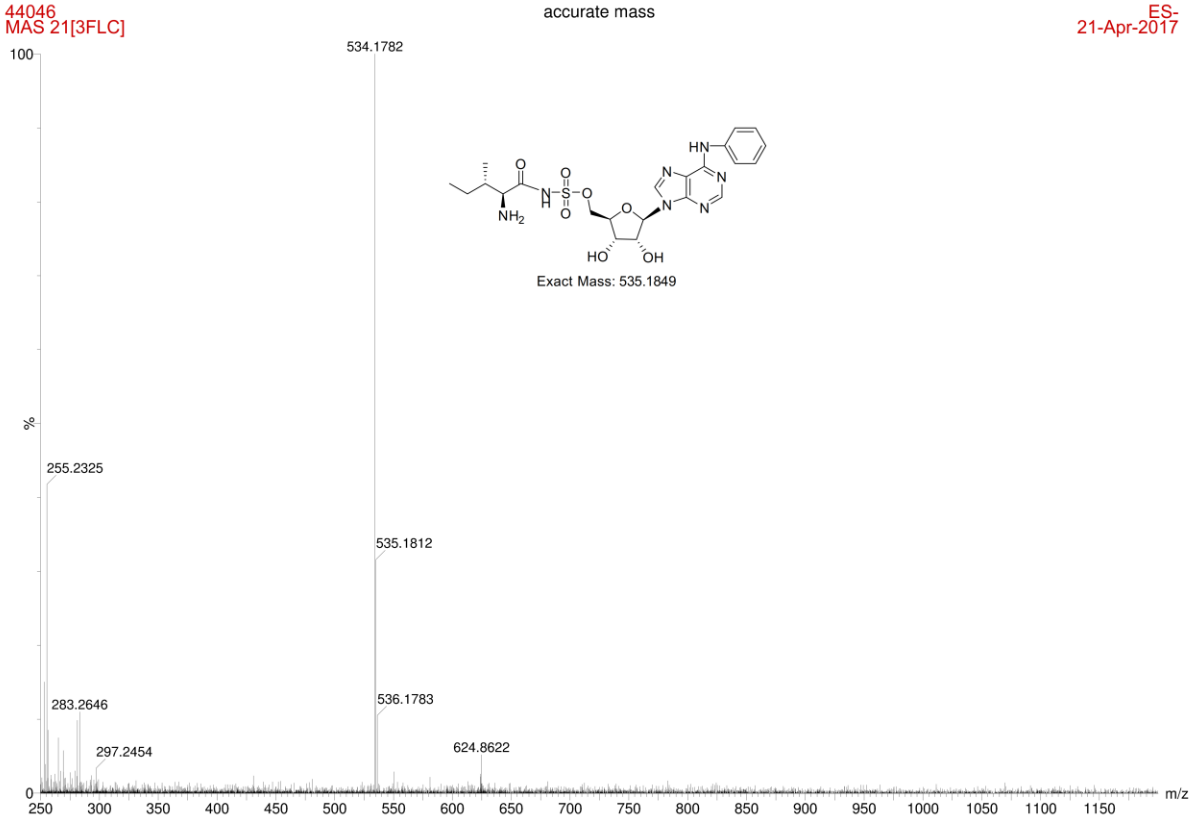

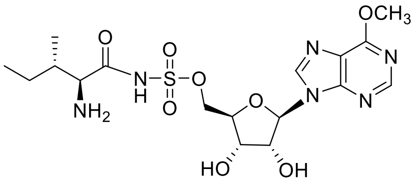

Exact Mass: 474.1533

5’-O-(N-L-isoleucyl)-sulfamoyl-6-O-methyl-purine riboside (6f)

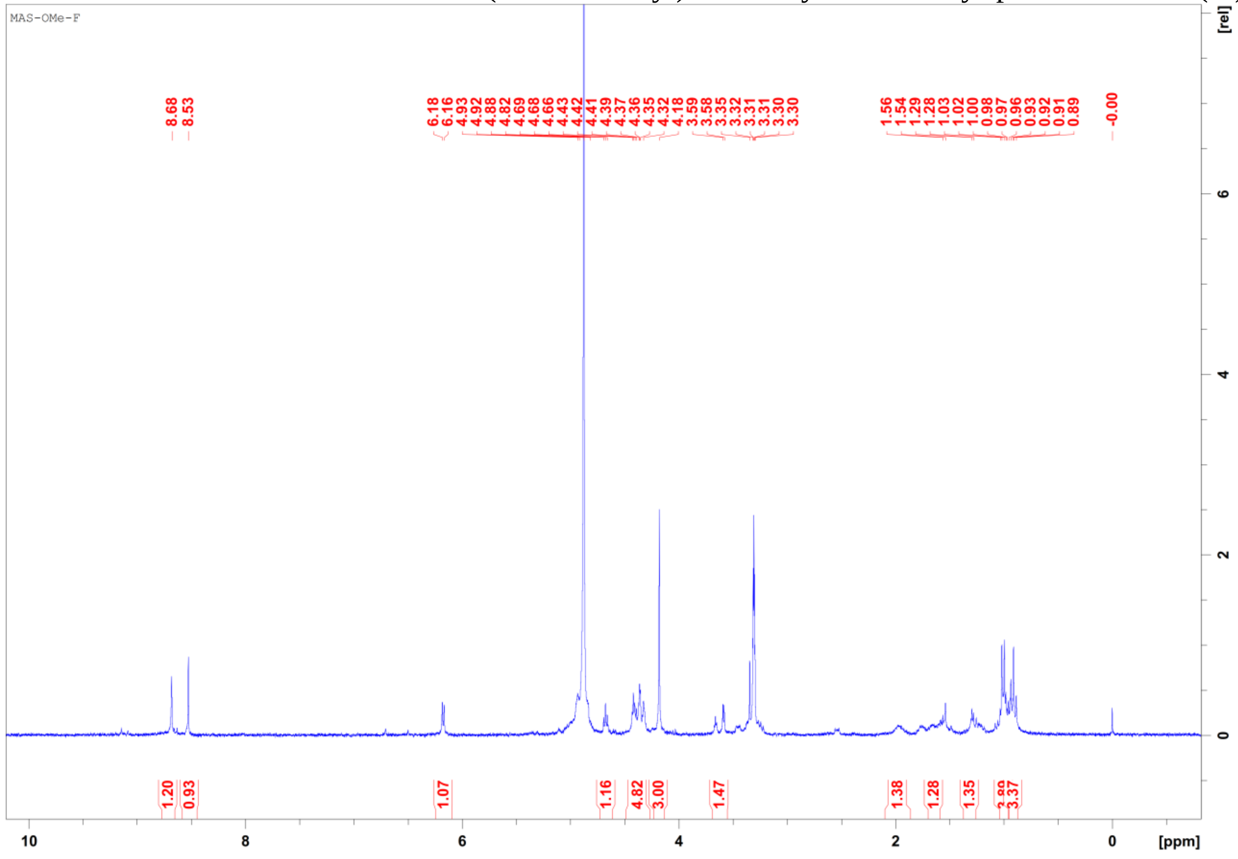

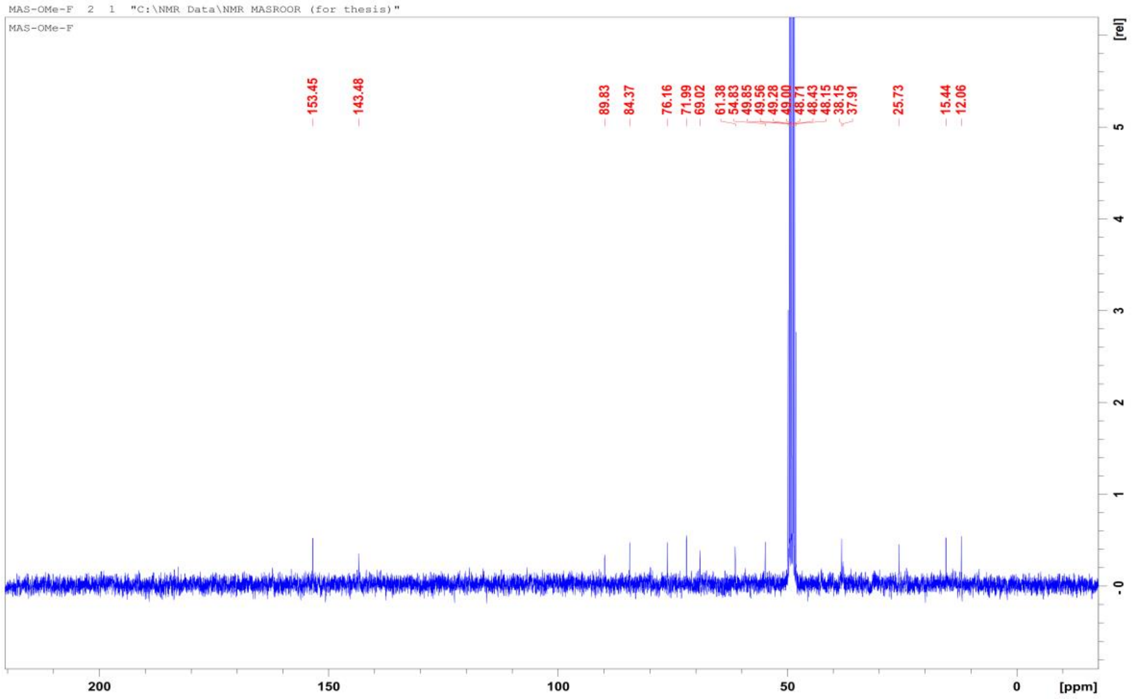
MAS-OMe-F 2 1 "C:\NMR Data\NMR MASROOR (for thesis)"
MAS-OMe-F
153.45
143.48
89.83
84.37
76.16
71.99
69.02
61.38
54.83
49.85
49.56
49.28
49.00
48.71
48.43
48.15
38.15
37.91
25.73
15.44
12.06
[rel]
200
150
100
50
0
[ppm]

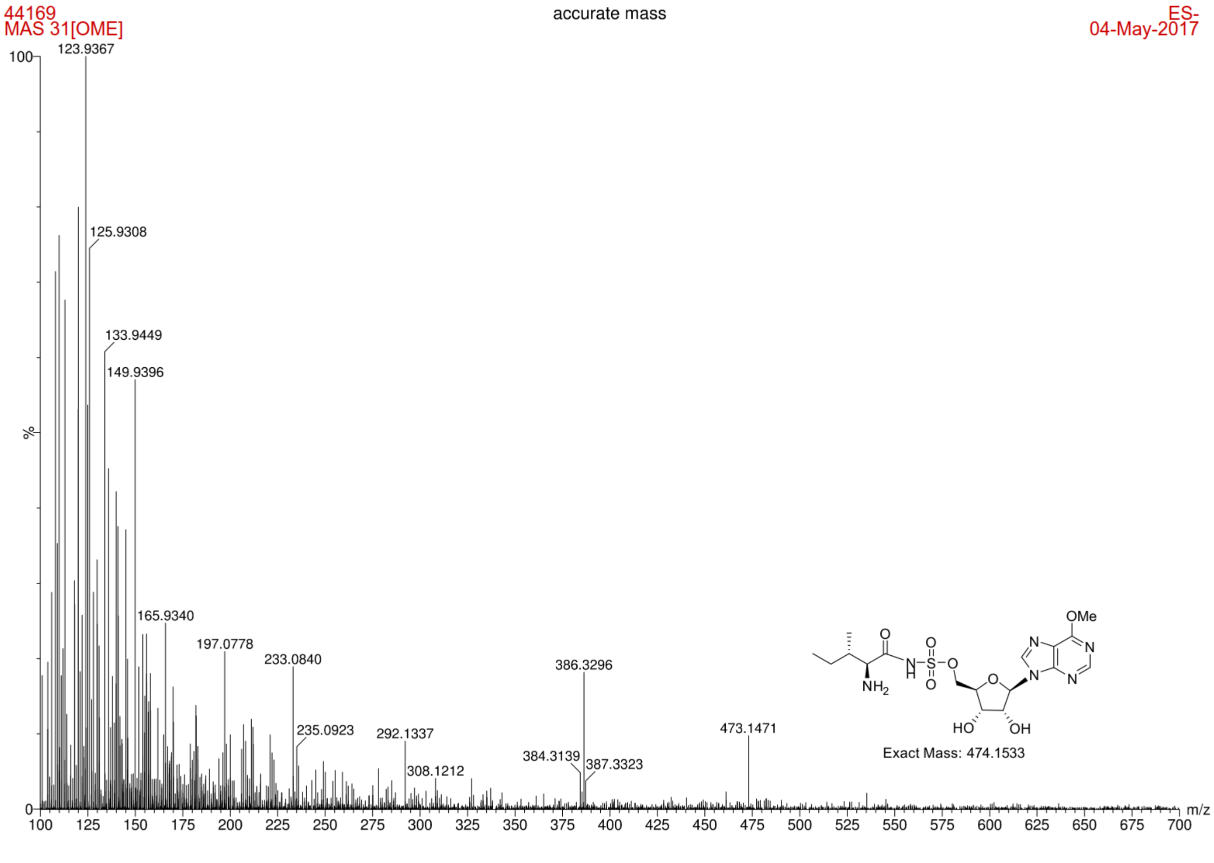
44169
MAS 31[OME]
accurate mass
ES-
04-May-2017
123.9367
125.9308
133.9449
149.9396
165.9340
197.0778
233.0840
235.0923
292.1337
308.1212
384.3139
386.3296
387.3323
473.1471
%
m/z
OMe
Exact Mass: 474.1533

Prodrugs intermediate & final compounds - NMR & MS

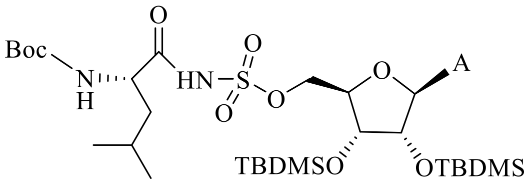

2',3'-di-*O*-TBDMS 5'-*O*-[*N*-(*N*-Boc)leucyl]sulfamoyl adenosine (11)

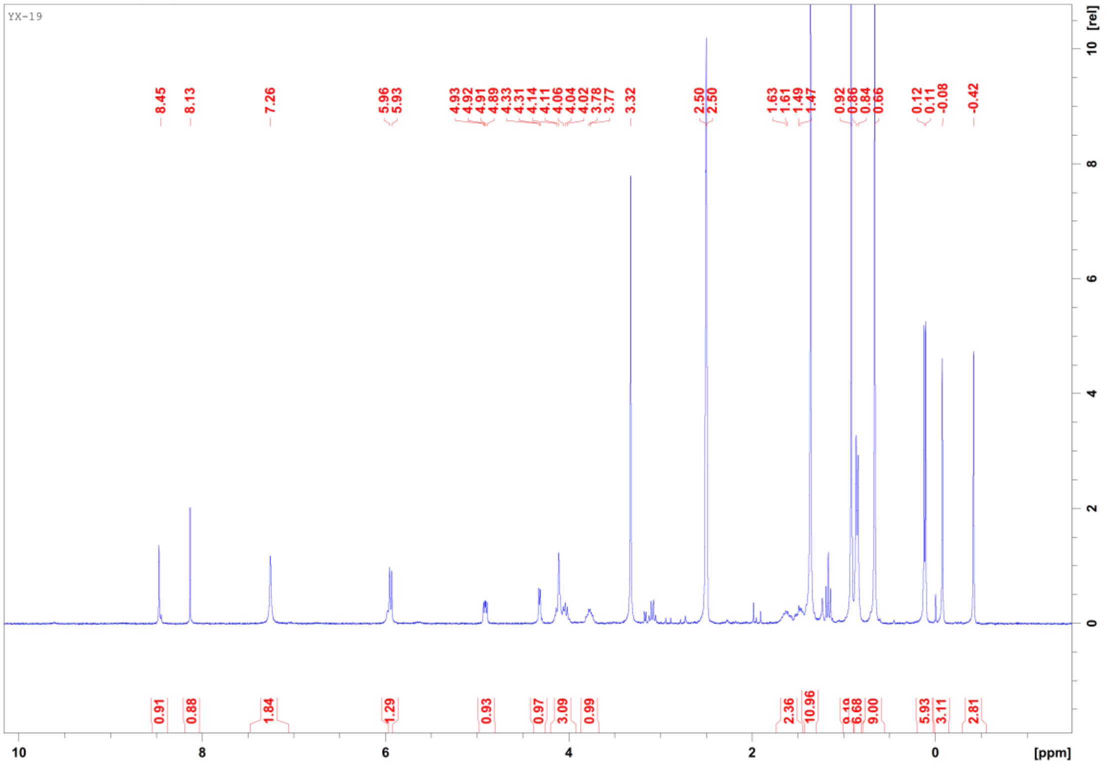

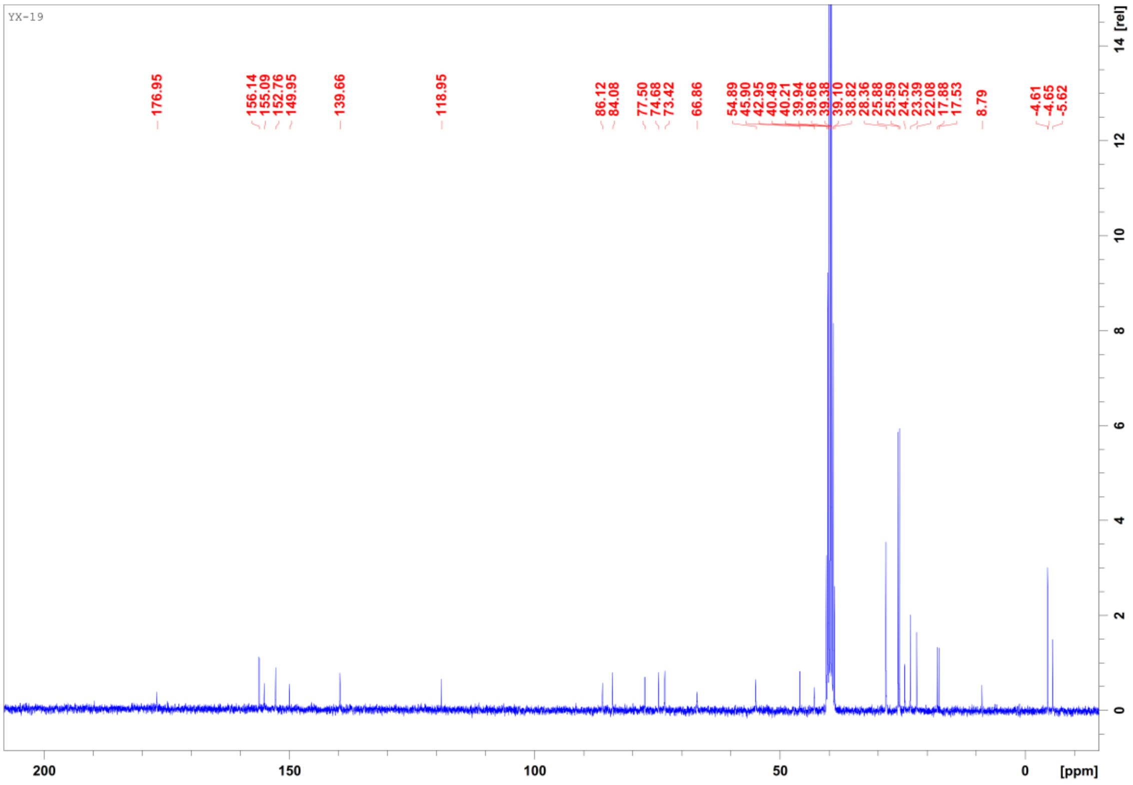

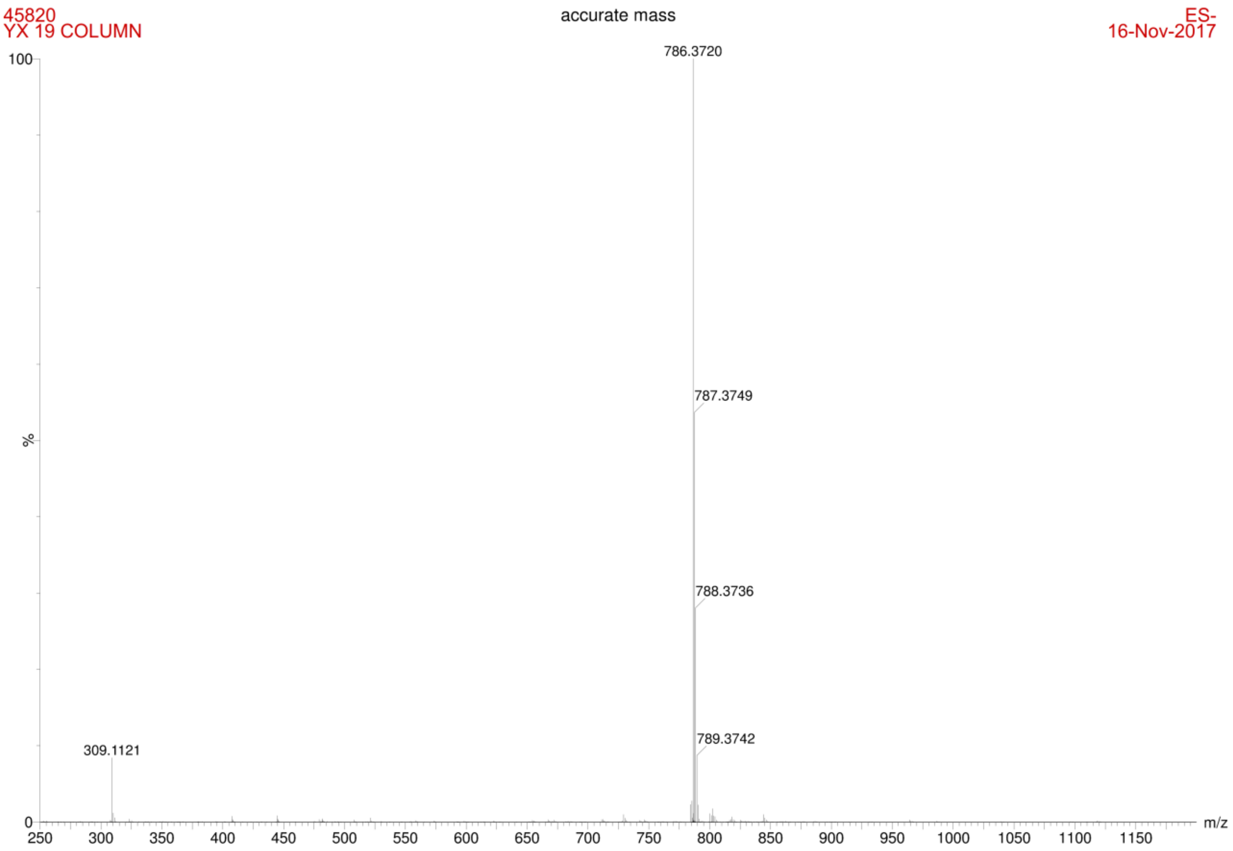

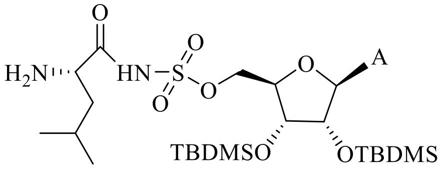

2',3'-di-O-TBDMS-5'-O-(N-leucyl)sulfamoyl adenosine (12)

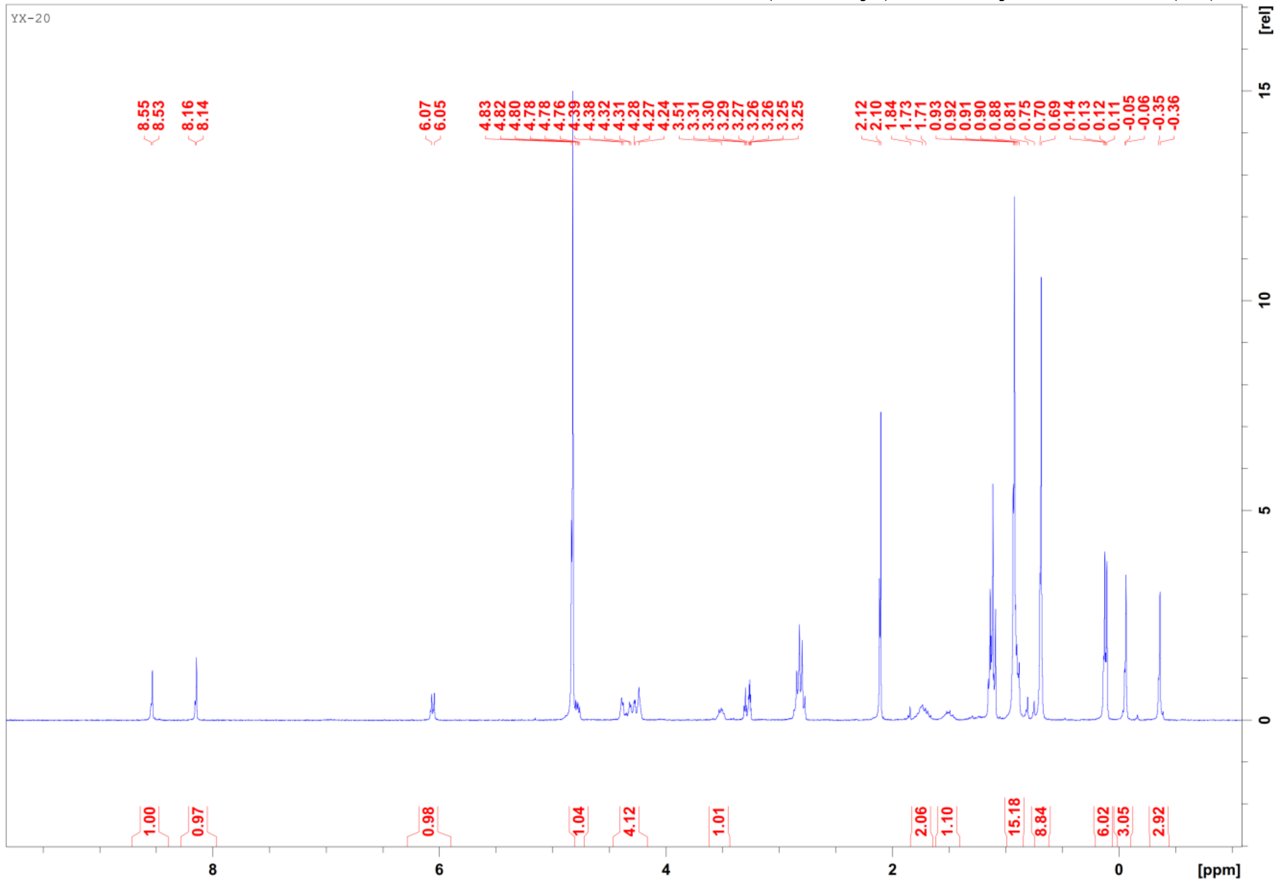

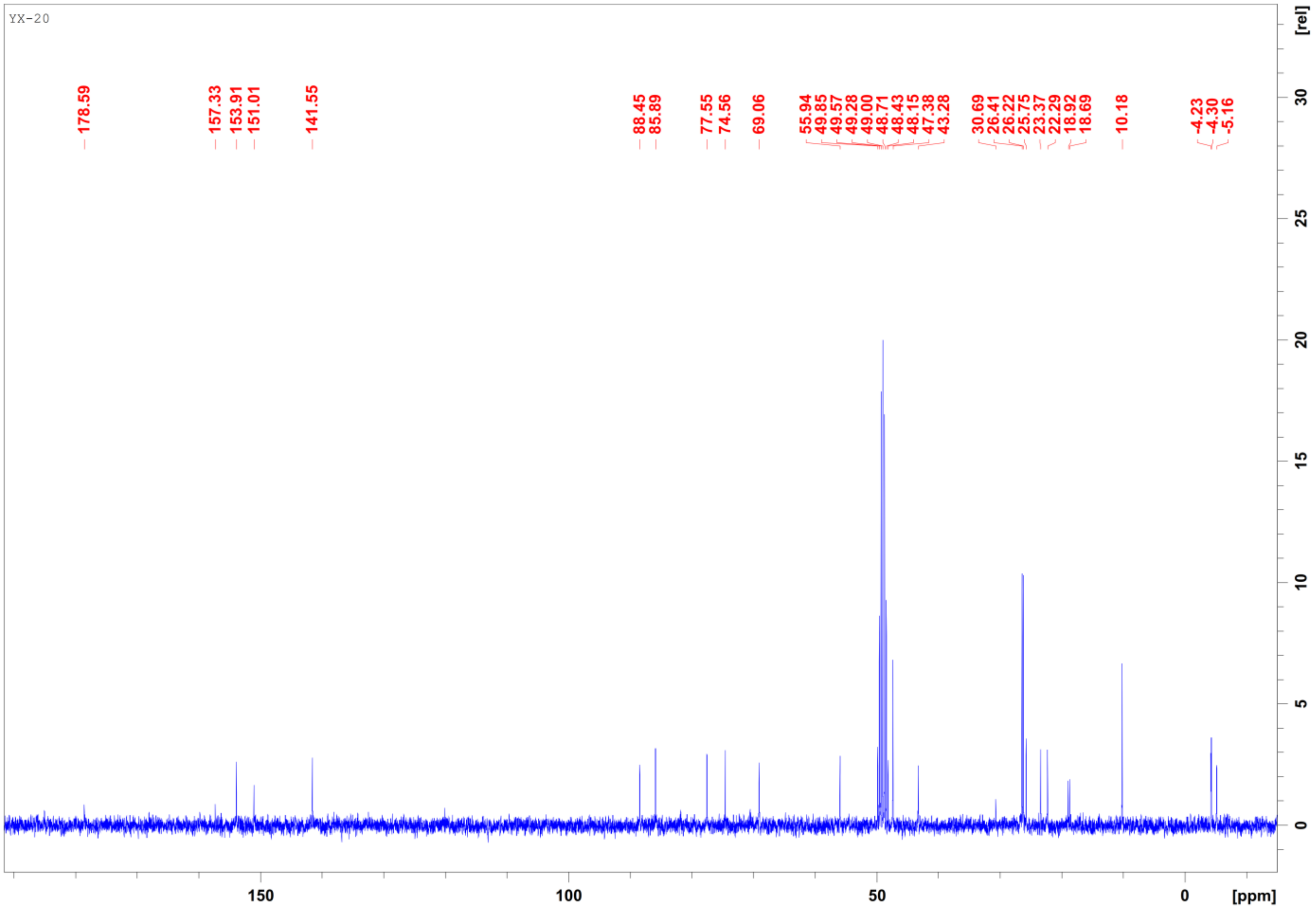
YX-20
178.59
157.33
153.91
151.01
141.55
88.45
85.89
77.55
74.56
69.06
55.94
49.85
49.57
49.28
49.00
48.71
48.43
48.15
47.38
43.28
30.69
26.41
26.22
25.75
23.37
22.29
18.92
18.69
10.18
-4.23
-4.30
-5.16
[rel]
[ppm]

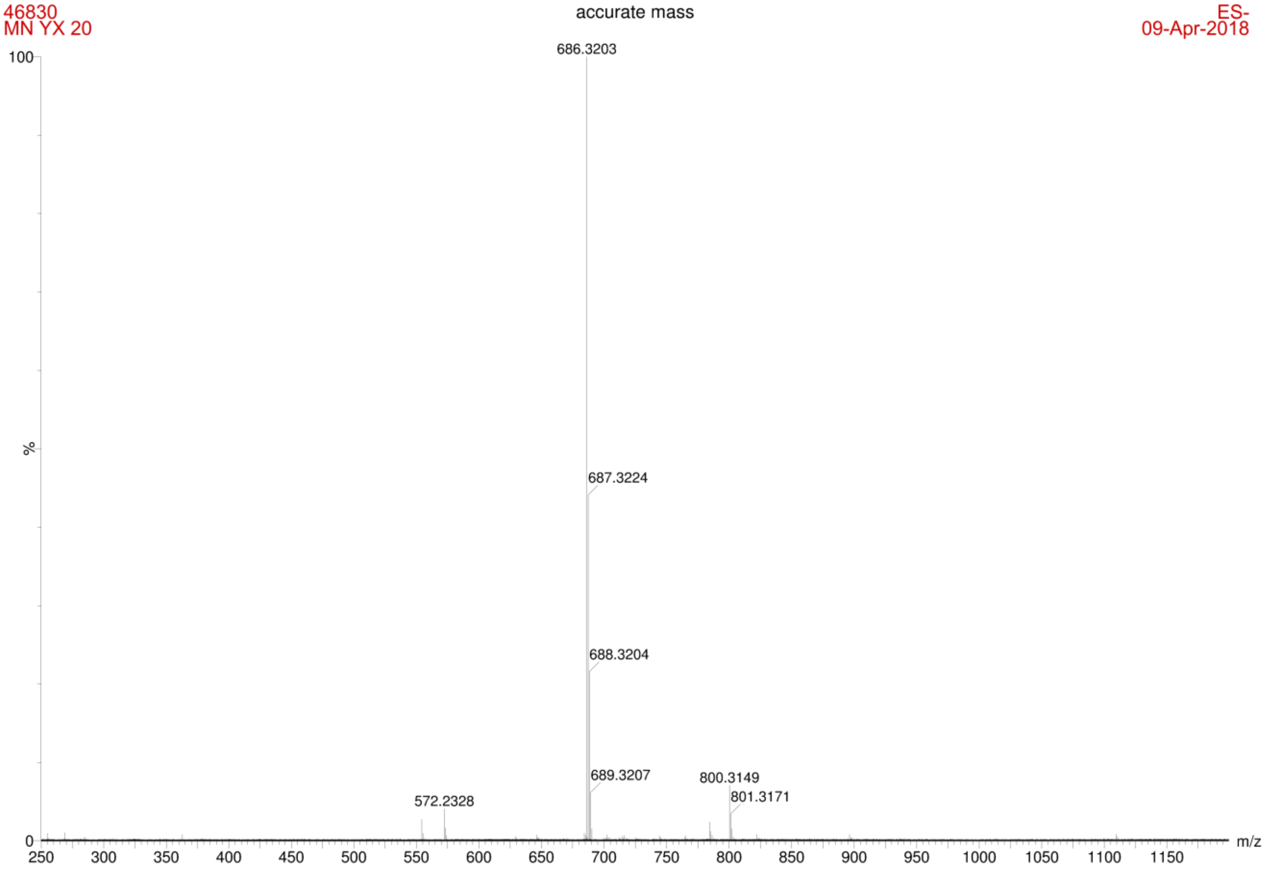
46830
MN YX 20
accurate mass
ES-
09-Apr-2018
686.3203
687.3224
688.3204
689.3207
572.2328
800.3149
801.3171
%
m/z

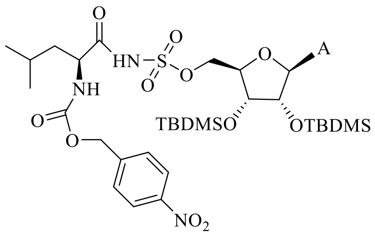

2',3'-di-O-TBDMS-5'-O-[Nα-(p-nitrobenzyloxycarbonyl)leucyl]sulfamoyl adenosine (13)

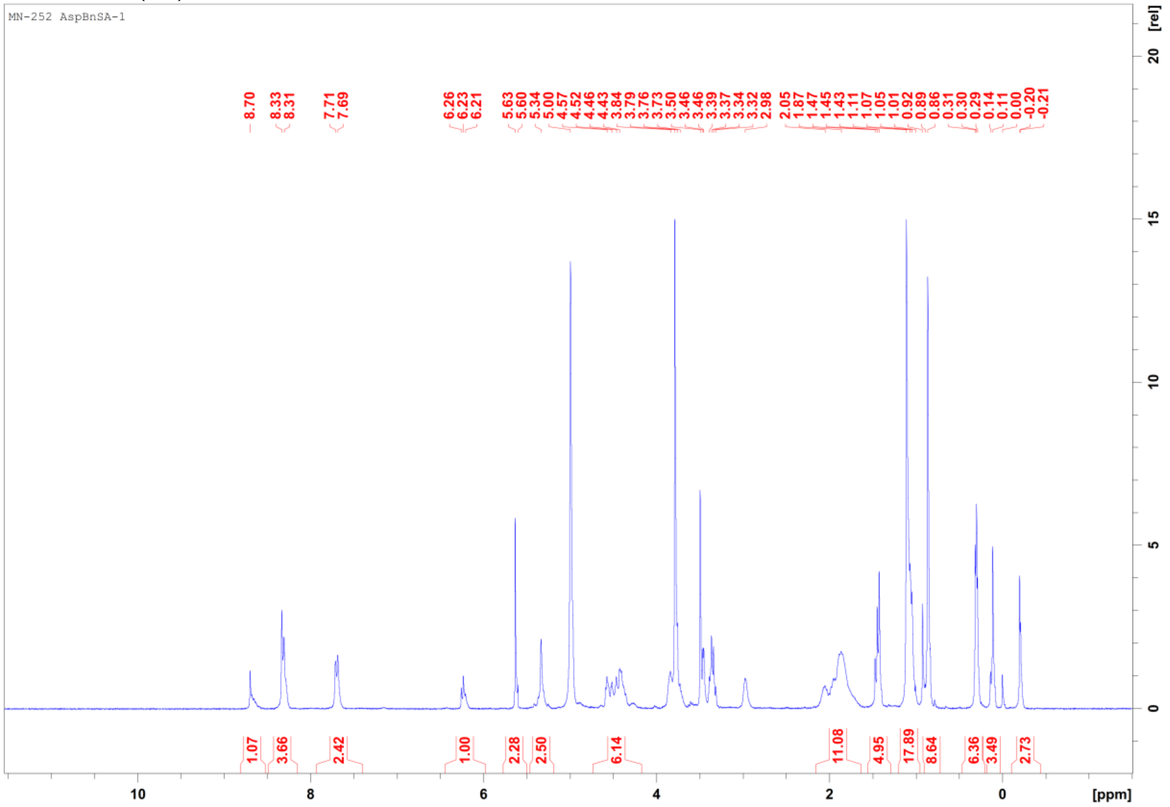

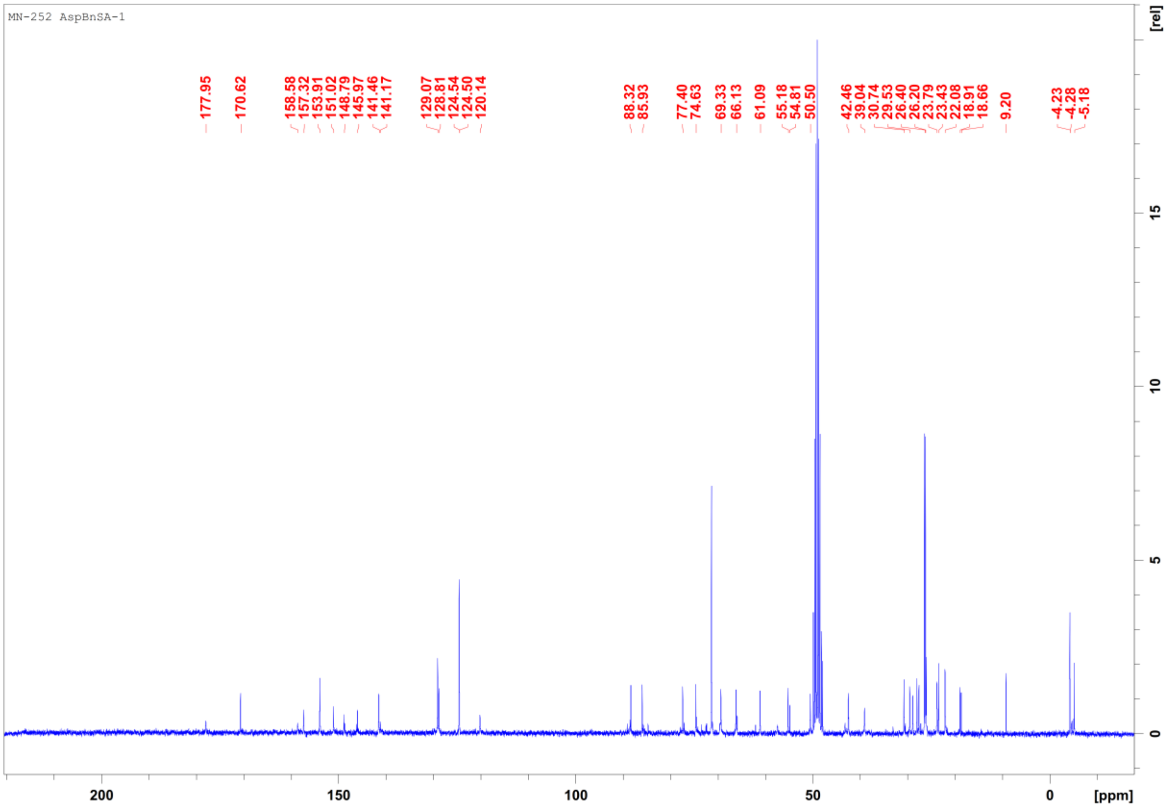

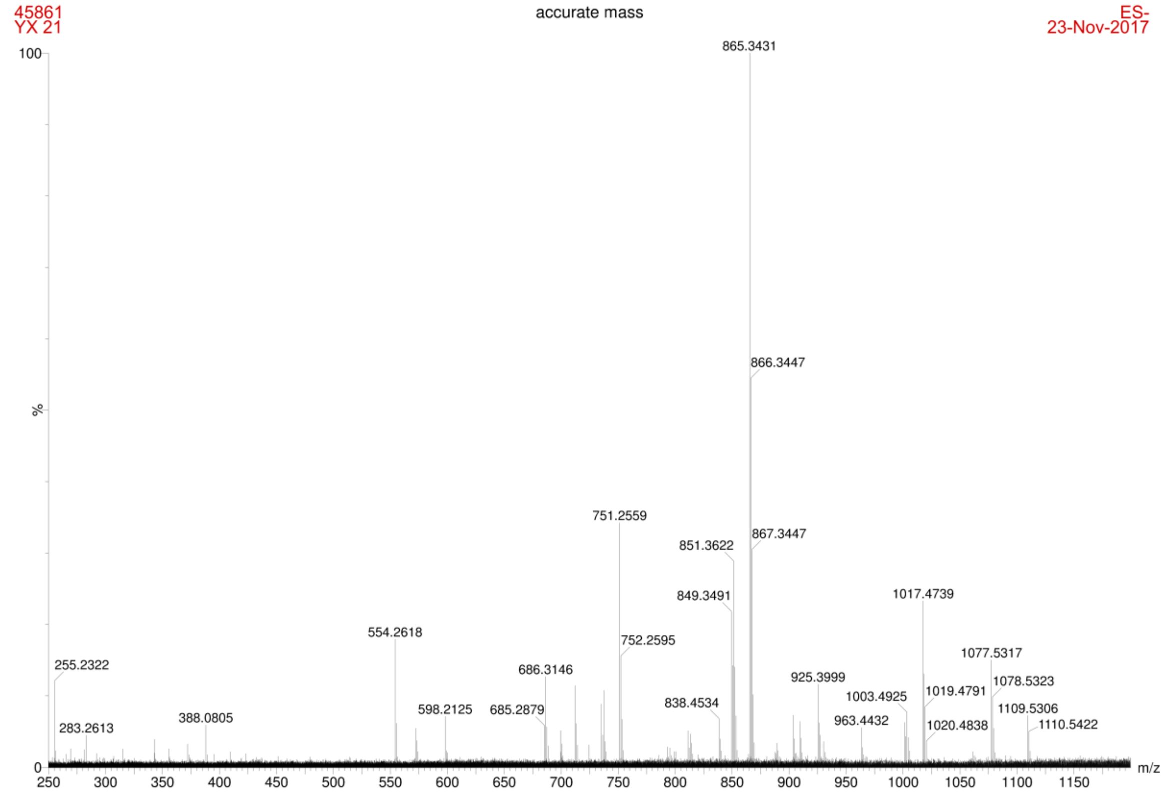

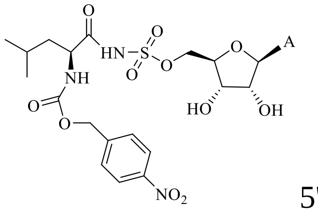

5'-O-[Nα-(p-nitrobenzyloxycarbonyl)leucyl]sulfamoyl adenosine (14)

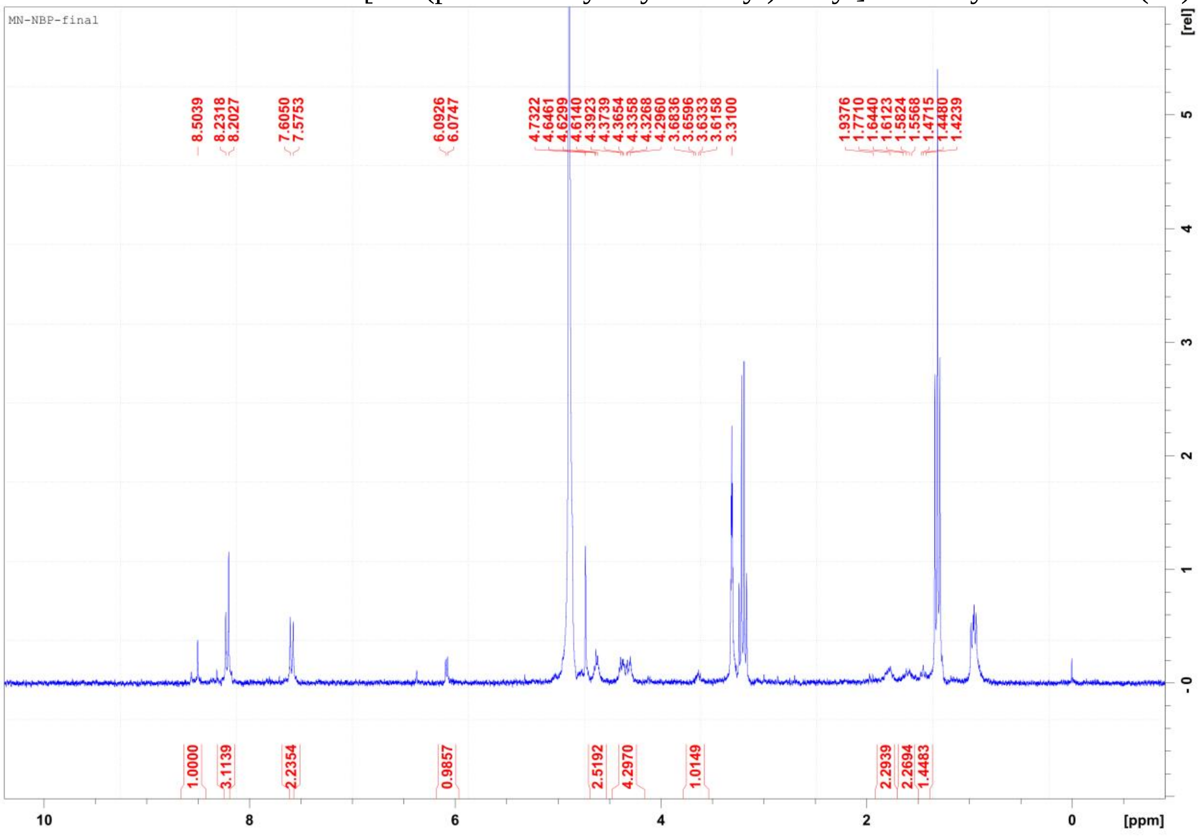

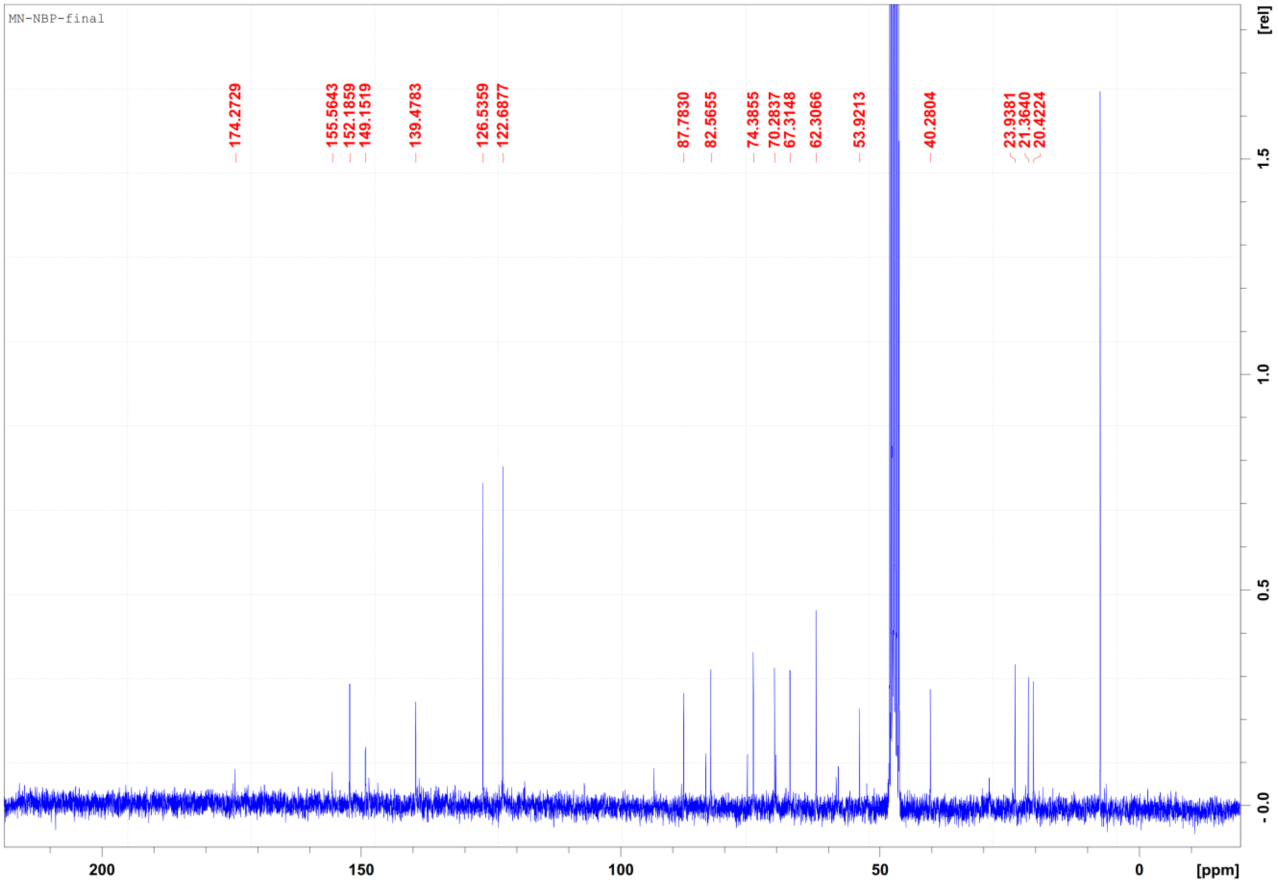
MN-NBP-final
174.2729
155.5643
152.1859
149.1519
139.4783
126.5359
122.6877
87.7830
82.5655
74.3855
70.2837
67.3148
62.3066
53.9213
40.2804
23.9381
21.3640
20.4224
[rel]
1.5
1.0
0.5
-0.0
200
150
100
50
0
[ppm]

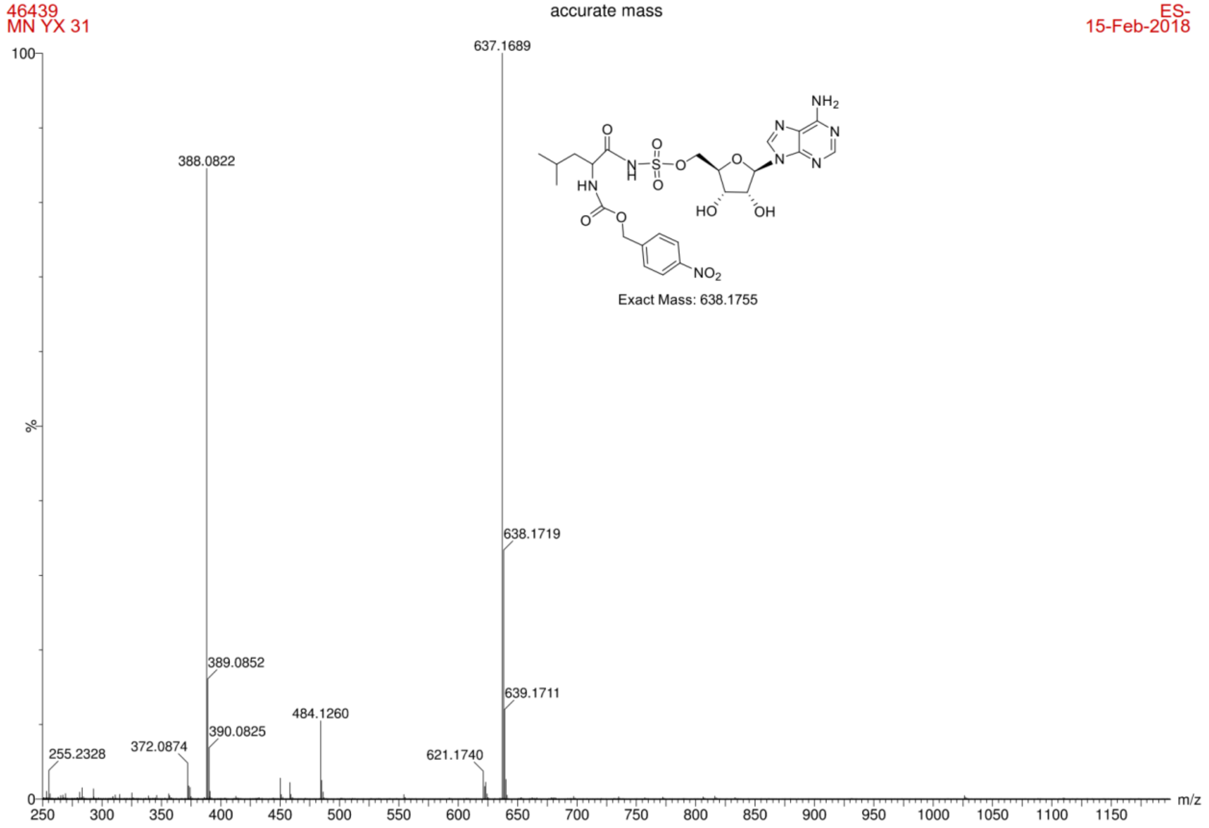
46439
MN YX 31
accurate mass
ES-
15-Feb-2018
100
%
0
637.1689
388.0822
638.1719
389.0852
639.1711
484.1260
390.0825
372.0874
255.2328
621.1740
m/z
NH2
N
O
HN
NO2
HO
OH
Exact Mass: 638.1755

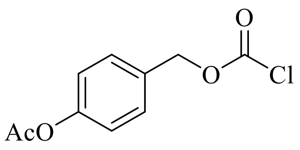

p-acetoxybenzyoxycarbonylchloride (16)

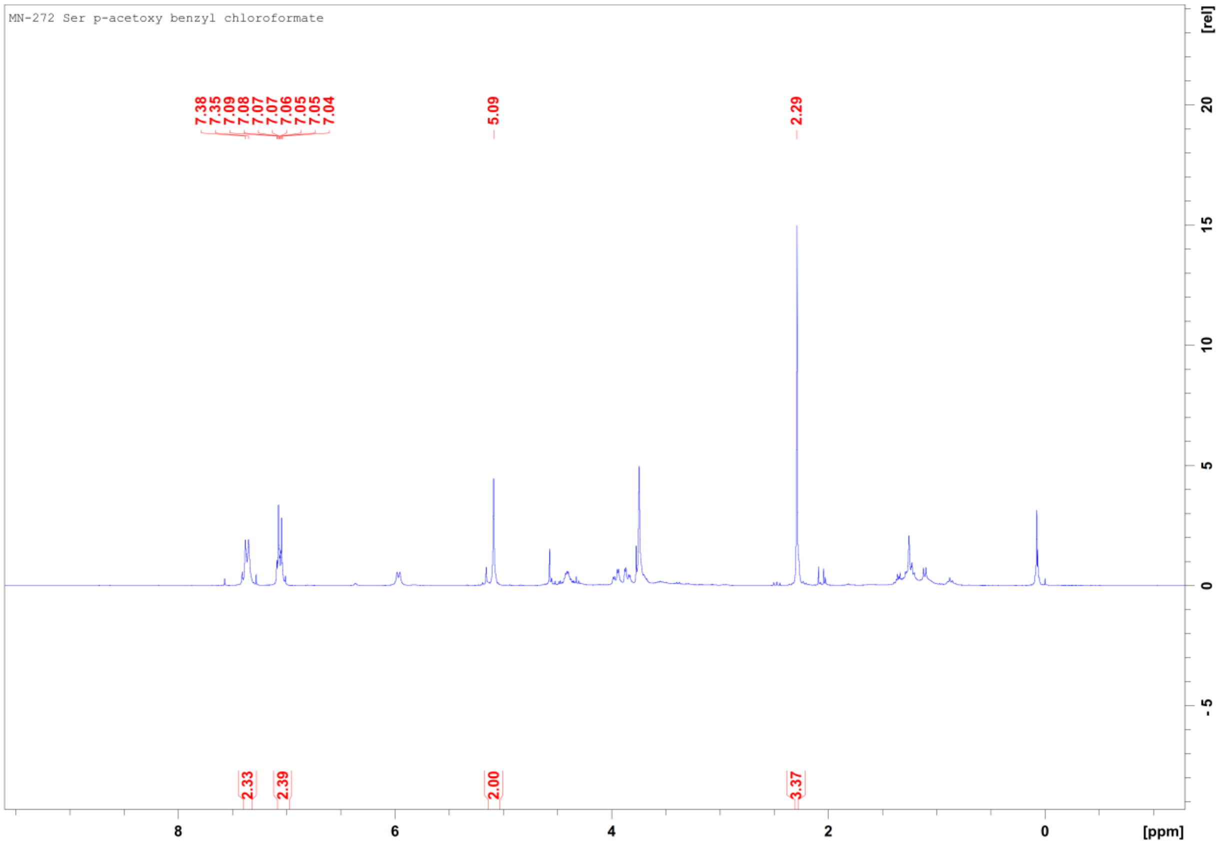

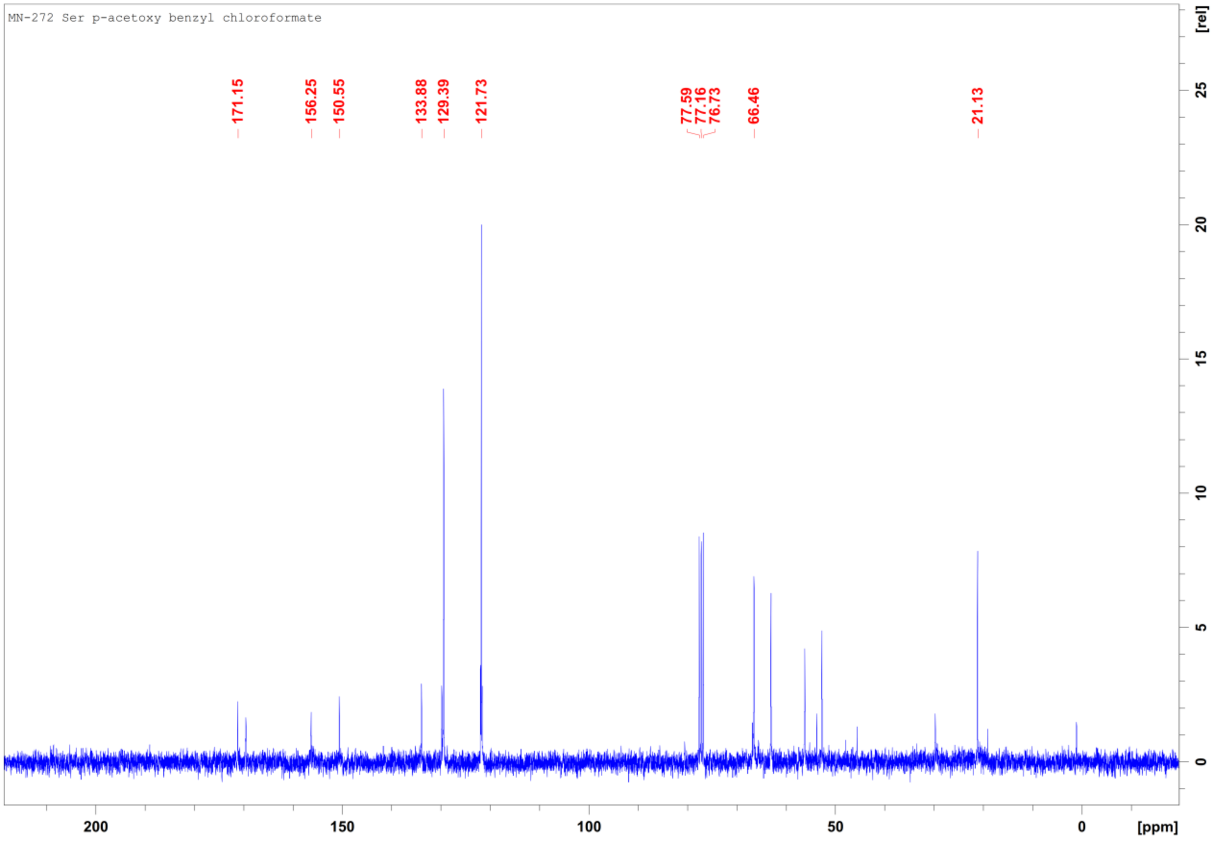

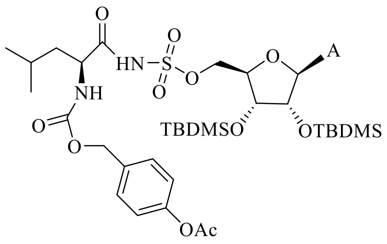

2',3'-di-*O*-TBDMS-5'-*O*-[N^{α}-(*p*-acetoxybenzyloxycarbonyl)leucyl] sulfamoyl adenosine (17)

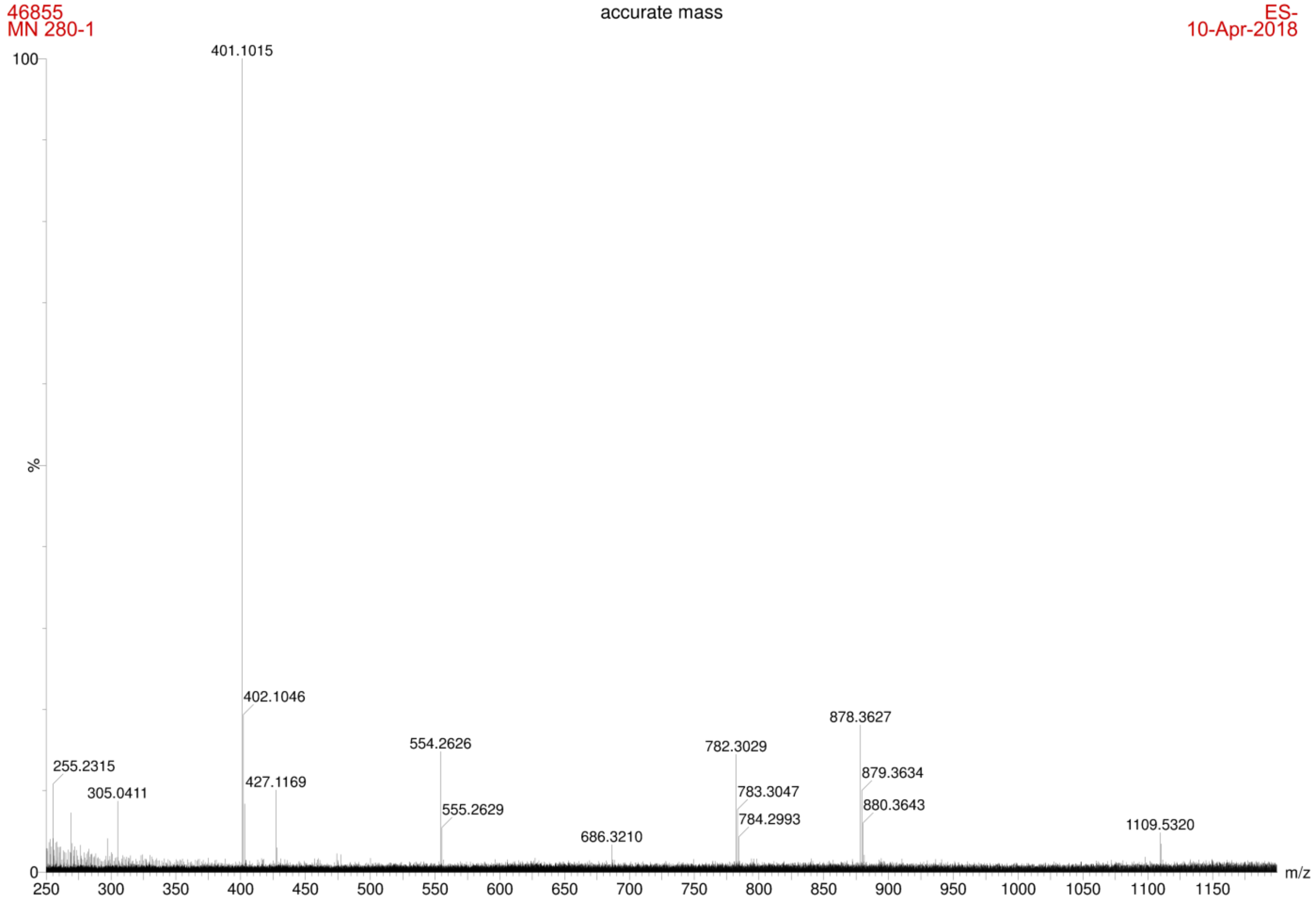

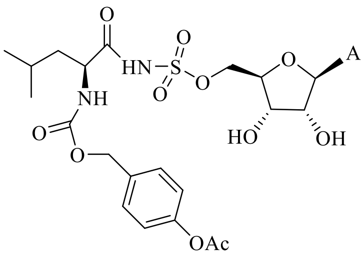

5'-O-[Nα-(p-acetyloxybenzyloxycarbonyl)leucyl]sulfamoyl adenosine (18).

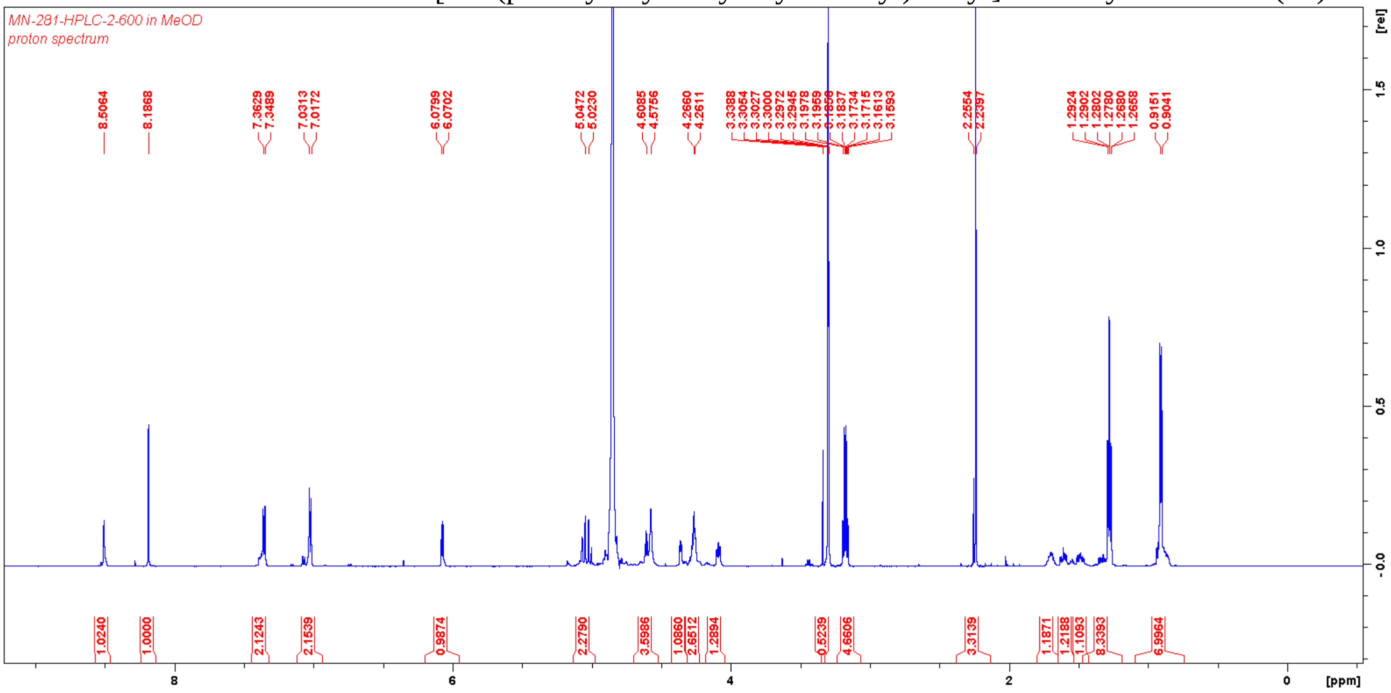

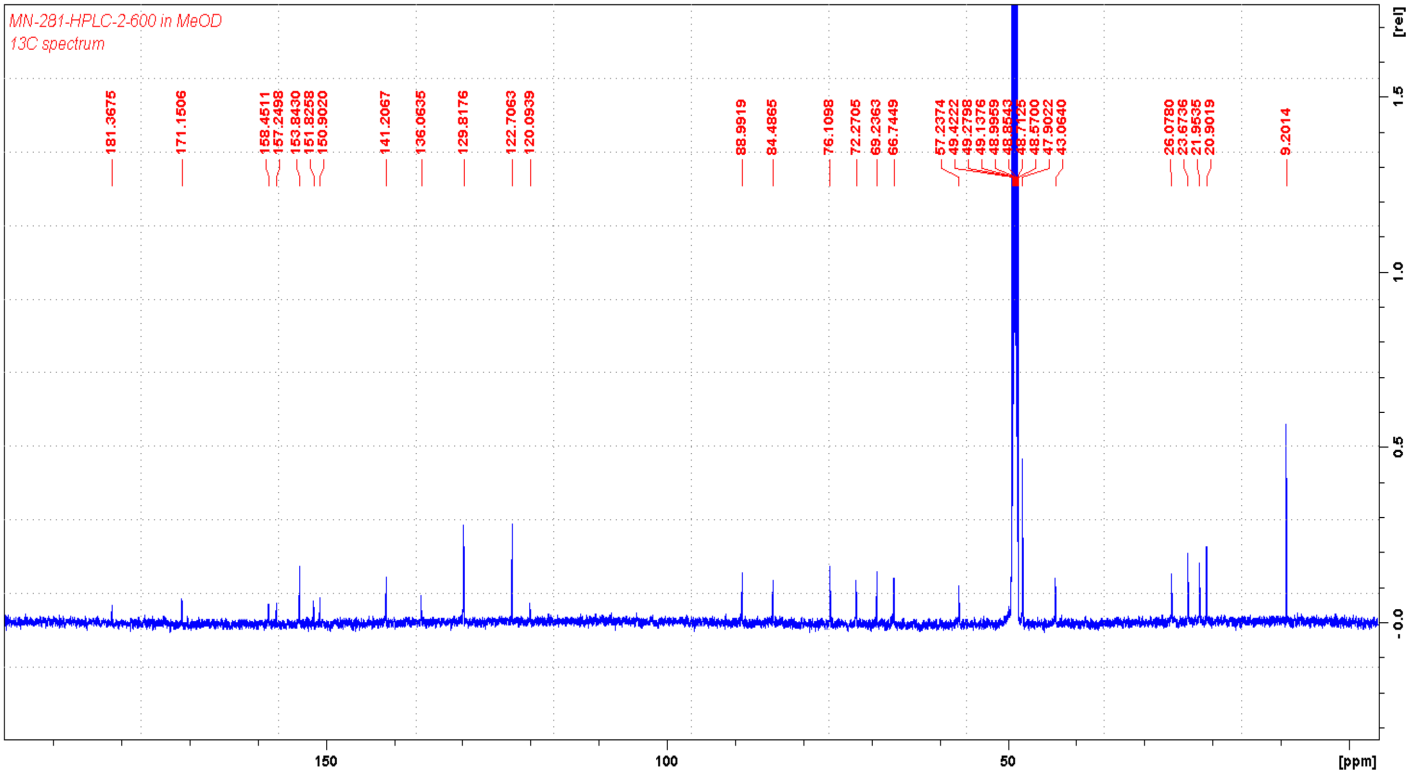

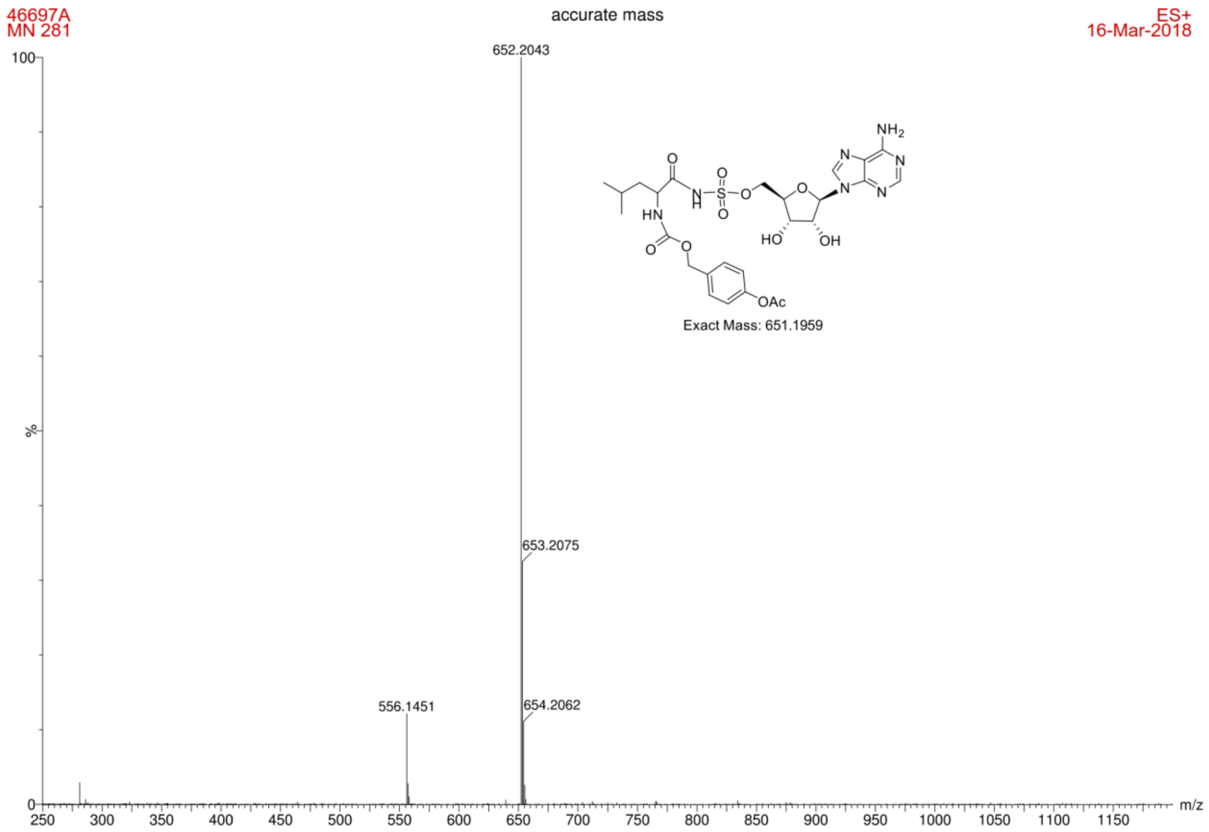
46697A
MN 281
accurate mass
ES+
16-Mar-2018
652.2043
653.2075
654.2062
556.1451
NH2
O
N
N
N
N
N
O
O
S
O
O
H
HN
O
O
HO
OH
OAc
Exact Mass: 651.1959
100
%
0
250
300
350
400
450
500
550
600
650
700
750
800
850
900
950
1000
1050
1100
1150
m/z
